# Detection of Copy‐Number Variations in CNS Tumours From Off‐Target Reads of Hybrid‐Capture Sequencing

**DOI:** 10.1111/nan.70070

**Published:** 2026-03-16

**Authors:** Jan Schnorrenberg, Yannis Luca Adrian, Judith Schlathölter, Christian Ruckert, Judit Horvath, Werner Paulus, Martin Hasselblatt, Christian Thomas

**Affiliations:** ^1^ Institute of Neuropathology University Hospital Münster Münster Germany; ^2^ Department of Medical Genetics, Centre of Medical Genetics (CMG) University and University Hospital Münster Münster Germany

**Keywords:** copy‐number variations, molecular diagnostics, next‐generation sequencing

## Abstract

Copy number variations (CNVs) play a central role in the classification, grading and prognostication of central nervous system (CNS) tumours. While genome‐wide methylation arrays are widely used for CNV profiling, next‐generation sequencing (NGS) panels are increasingly implemented in routine diagnostics. We hypothesised that off‐target sequencing reads from small hybrid‐capture panels not specifically designed for copy‐number detection can yield clinically actionable genome‐wide CNV profiles. We analysed 60 CNS tumour samples, including glioblastomas, oligodendrogliomas, ependymal tumours, medulloblastomas and choroid plexus tumours using a small‐scale custom hybrid‐capture panel (< 0,2 Mb) and compared CNV profiles inferred from sequencing reads to those obtained with methylation arrays. Additionally, 58 meningiomas and 6 pilocytic astrocytomas with *BRAF* fusions were profiled with the same NGS panel. Across 527 chromosomal arm‐level alterations, concordance between NGS‐ and methylation‐derived profiles was 95%. All 19 focal amplifications (e.g., *EGFR*, *MDM4* and *MYCN*) and the majority of homozygous *CDKN2A/B* deletions were correctly detected. In meningiomas, genome‐wide CNV profiling from off‐target reads identified WHO‐relevant alterations, including *CDKN2A/B* deletions and 1p/22q co‐deletions, supporting molecular upgrading in 9/58 (16%) of histologically lower grade tumours. Focal copy‐number variations on Chr7q suggestive of *BRAF* fusions were observed in 5/6 fusion‐positive pilocytic astrocytomas. These findings demonstrate that off‐target reads from minimal targeted NGS panels can generate genome‐wide CNV profiles, comparable to methylation array data, without the need for additional assays or specialised probe designs.

## Summary

- Off‐target reads from small hybrid‐capture NGS panels can be leveraged to generate genome‐wide copy‐number profiles.
- Clinically relevant chromosomal arm‐level changes and focal amplifications can be reliably detected using off‐target sequencing reads.
- This approach can be incorporated into existing diagnostic workflows without requiring additional assays or specialised panel designs.

## Graphical abstract

Off‐target sequencing reads from small hybrid‐capture NGS panels can be leveraged to reconstruct genome‐wide copy number variation (CNV) profiles in CNS tumours. Across diverse tumour types, CNV profiles derived from off‐target reads show high concordance with methylation array data and reliably detect clinically relevant arm‐level alterations, homozygous deletions and even nontargeted focal amplifications. This strategy enables comprehensive molecular classification and prognostication without additional assays, maximising diagnostic yield from routine targeted sequencing.

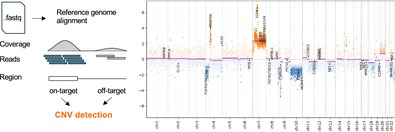

## 1 Introduction

Copy number variations (CNVs) have emerged as critical genomic alterations in the neuropathological evaluation of central nervous system (CNS) tumours, with essential roles in diagnostic classification, prognostic assessment and therapeutic decision‐making [1]. These alterations encompass a spectrum from large, chromosome‐wide changes to focal amplifications or deletions involving single genes [2]. Among the most clinically significant and historically recognised CNVs are the co‐deletion of chromosomal arms 1p and 19q [3], a defining feature of oligodendroglioma, IDH‐mutant and the +Chr7/−Chr10 signature characteristic of glioblastoma, IDH‐wildtype [4]. More recently, loss of Chr1p and *MYCN* amplification have been incorporated into the WHO classification as essential diagnostic markers for diffuse leptomeningeal glioneuronal tumour (DLGNT) and *MYCN*‐amplified spinal ependymoma, respectively, highlighting the broad diagnostic relevance of CNVs across tumour types [5, 6]. Furthermore, focal CNVs such as *EGFR* amplification in glioblastoma [2] and *MYCN* amplification in medulloblastoma [7], and *CDKN2A/B* homozygous deletion in IDH‐mutant astrocytoma [8] and meningioma [9] are particularly important for determining tumour grade and prognosis.

Copy‐number profiling is commonly performed using DNA methylation arrays or chromosomal microarrays; however, next‐generation sequencing (NGS) panels have increasingly been employed to extract CNV information alongside mutational data in a single assay [10, 11, 12, 13, 14]. In the context of CNS tumours, several studies have demonstrated the feasibility of detecting diagnostically relevant alterations such as 1p/19q co‐deletion, *EGFR* amplification and *CDKN2A/B* homozygous deletion from targeted panels that were specifically designed to capture copy‐number information, either through gene‐centric coverage or additional probes targeting broader genomic regions [12, 14]. While some assays incorporate tiling probes or genome‐wide SNP coverage to explicitly enable CNV detection [11, 15], an alternative approach leverages off‐target sequencing reads, that is, those that map outside of the intended capture regions, as a ‘side‐product’ of hybrid‐capture protocols [16, 17]. Although bioinformatic tools such as CNVkit [17] allow inference of CNVs from off‐target reads, this approach has not yet been systematically applied to CNS tumours or compared against established methods such as DNA methylation arrays. Thus, the diagnostic value and reliability of off‐target‐derived CNV profiles in neuro‐oncology remain largely unexplored. Nonetheless, the ability to extract copy‐number information from off‐target reads may significantly enhance the diagnostic yield of small‐scale targeted panels, particularly in settings where material is limited or the panel was not initially designed for CNV detection.

In this study, we explored the feasibility of generating genome‐wide copy‐number profiles from off‐target reads obtained with a small, custom‐designed 31‐gene hybrid‐capture gene panel lacking a specific CNV‐enabling probe design. We evaluated whether these profiles could reliably capture diagnostically relevant CNVs in CNS tumours and compared them to CNV profiles obtained from DNA methylation arrays, a current reference standard in CNS tumour diagnostics [2]. We aimed to determine whether off‐target read analysis offers a reliable, low‐cost complement to existing sequencing workflows.

## 2 Materials and Methods

### 2.1 Materials

Formalin‐fixed paraffin‐embedded (FFPE) samples from 60 CNS tumours across different entities (Table S1), plus 58 meningiomas (Table S2) and 6 pilocytic astrocytomas (Table S3), were retrieved from the archive of the Institute of Neuropathology in Münster. The use of biopsy specimens for research upon anonymisation was in accordance with local regulations of the University Hospital Münster and approved by the Münster ethics committee (2007‐420‐f‐S and 2017‐707‐f‐S).

### 2.2 DNA Isolation

Genomic DNA was extracted from FFPE samples using the Maxwell RSC FFPE Plus DNA Kit (AS1720, Promega, Madison, USA). DNA was eluted in 50 μL of 1 × TE buffer (pH 7.5; Promega) and quantified using the QuantiFluor ONE dsDNA System (Promega). DNA integrity (DIN) values and concentrations were assessed with the Genomic DNA ScreenTape Assay on the TapeStation platform (Agilent Technologies, Santa Clara, USA).

### 2.3 Library Preparation and Next‐Generation Sequencing

Genomic DNA integrity (DIN) and concentration were assessed using the Genomic DNA ScreenTape Assay on the TapeStation system (Agilent Technologies, Santa Clara, USA), with the region of interest set from 50 bp to > 60,000 bp. Libraries were prepared using different versions of custom hybrid‐capture panels (Twist Bioscience, San Francisco, USA), targeting genes selected exons, the *TERT* promoter and intronic regions for gene fusion detection in CNS tumour diagnostics (Table S4). Across the cohort, *n* = 10 samples were hybridised with panel version 2 (total size: 137 kb), *n* = 11 samples were hybridised with panel version 3 (total size: 161 kb) and *n* = 39 samples were hybridised with panel version 4 (total size: 171 kb). Fragmentation times and the number of PCR cycles were adjusted according to DIN values, following the manufacturer's recommendations. Final libraries were sequenced on an Illumina NextSeq platform (Illumina, San Diego, USA).

### 2.4 Bioinformatic Analysis of NGS Data

Copy‐number profiling was conducted using CNVkit, which uses both on‐target and off‐target reads from hybrid‐capture sequencing to infer genome‐wide copy‐number alterations [17]. On‐target read depth provides high resolution where probes are designed, while off‐target read depth is binned across the remainder of the genome to improve coverage uniformity and enable detection of large‐scale events beyond targeted regions. Coverage profiles for both target and antitarget bins are normalised and bias‐corrected using a reference constructed from a pooled cohort of normal samples or tumour samples with flat CNV profiles (*n* = 8) processed with the same panel and protocol, which mitigates systematic biases such as GC content and capture efficiency. After adapter trimming with fastp and alignment against the hg19 reference genome using BWA mem, CNVkit was run with default settings, and the reference coverage was used to calculate log2 copy‐ratio estimates for each bin across the genome.

For gene fusion analysis, paired‐end reads were aligned to the hg19 reference genome using STAR with chimeric read detection enabled. Fusion calling was subsequently performed with Arriba using the GENCODE v19 annotation together with curated databases of known fusions and blacklisted events. Standard filtering settings were applied. Fusion candidates were visualised using the Arriba plotting scripts and manually reviewed.

### 2.5 DNA Methylation Profiling

Following purification and bisulfite conversion using standard protocols, DNA methylation profiling was performed with the Infinium MethylationEPIC BeadChip array (Illumina, San Diego, USA), with 21 samples analysed on the EPIC (850 K) array and 39 on the EPICv2 (935 K) array. Tumour classification was carried out using the Heidelberg Brain Tumour Classifier (v12b8), and copy‐number alterations were inferred with the conumee2.0 R package [18].

### 2.6 Bioinformatic Analysis for Comparative CNV Analysis

Copy‐number segmentation (.seg) and bin (.igv) files were generated from DNA methylation data using the conumee2.0 R package, and copy‐number ratio files (.cnr) were derived from hybrid‐capture sequencing data using CNVkit. Comparative analyses were performed by calculating the correlation of copy‐number profiles both genome‐wide and on a per‐chromosome arm basis. Sex chromosomes were excluded from all correlation analyses. Data processing and statistical analyses were conducted in Python 3.13 using the pandas and numpy libraries, and visualisations were generated using matplotlib and seaborn. All analysis scripts are available on GitHub (https://github.com/yansch/cnv‐from‐ngs/). For the final genome‐wide CNV visualisation, the following genes or gene regions were highlighted: *MDM4*, *MYCN*, *GLI2*, *FGFR3/TACC3*, *PDGFRA*, *TERT*, *MYB*, *EGFR*, *CDK6*, *MET*, *BRAF/KIAA1549*, *FGFR1/TACC1*, *MYBL1*, *MYC*, *CDKN2A*, *PTCH1*, *PTEN*, *MGMT*, *CCND1*, *CCND2*, *CDK4*, *MDM2*, *RB1*, *TP53*, *NF1*, *PPM1D*, *C19MC*, *SMARCB1* and *NF2*. For manual review, copy‐number profiles were visually inspected to identify chromosome arm–level gains or losses (defined as affecting ≥ 50% of the chromosomal arm) and focal amplifications or deletions.

## 3 Results

### 3.1 Study Cohort

**FIGURE 1:**
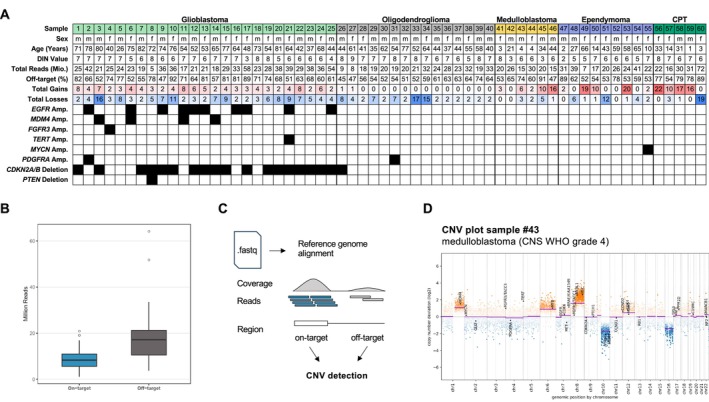
Overview of the sample cohort and CNV detection strategy from NGS data. (A) Summary of the sample cohort, including clinical and genetic information. Black boxes indicate the presence of genetic alterations. DIN, DNA integrity number. (B) Box plots showing the distribution of on‐target and off‐target reads across all samples. (C) Schematic illustration of the CNV detection pipeline integrating both on‐target and off‐target sequencing reads. (D) Example CNV profile from sample #43 (medulloblastoma, CNS WHO grade 4), highlighting chromosomal gains and losses identified by the pipeline.

Our cohort comprised 60 CNS tumours from 51 adult and 9 paediatric patients (27 female and 33 male), including IDH‐wildtype glioblastomas (*n* = 25), oligodendrogliomas (*n* = 15), ependymal tumours (*n* = 9), medulloblastomas (*n* = 6), and choroid plexus tumours (*n* = 5) (Figure 1A, Table S1). The average tumour cell content, as estimated from H&E slides, was 72% (range: 20%–95%, Table S1). Seven of 60 samples (12%) had a tumour cell content < 50%, all of which were from the infiltration zones of glioblastomas (samples #7, #11, #15, #16, #18, #21 and #25). All samples were analysed using the Illumina EPIC methylation array and classified with the v12.8 version of the Heidelberg CNS tumour classifier (median calibrated score: 0.99, Table S1).

Genome‐wide copy‐number profiles generated with conumee 2.0 [18] identified 527 chromosomal arm‐wide changes with 253 chromosomal arm gains and 274 arm losses. Only one medulloblastoma (sample #42) and one posterior fossa subependymoma (sample #52) exhibited balanced CNV profiles without copy‐number variations. Glioblastomas largely displayed the expected signature of whole‐chromosome 7 gain with monosomy 10; only two cases harboured isolated 7q gains and one case an isolated 10q loss (Table S1). Among oligodendrogliomas, in addition to the canonical 1p/19q codeletion, 6 of 15 cases showed a total of 46 additional chromosomal arm losses, and 2 cases exhibited a single chromosomal arm gain. Across entities, choroid plexus tumours demonstrated the highest CNV burden (mean 17.6 chromosomal arm alterations per sample), followed by glioblastomas (9.5 per sample), ependymomas (8.9 per sample) and medulloblastomas (8 per sample), whereas oligodendrogliomas carried the lowest average number of CNVs (5.2 per sample).

Focal CNVs were most frequent in glioblastomas, with 17 amplifications detected across 13 cases. These included *EGFR* (*n* = 10), *MDM4* (*n* = 4), *FGFR3* (*n* = 1), *TERT* (*n* = 1) and *PDGFRA* (*n* = 1). The case with *FGFR3* amplification (sample #4) harboured a *FGFR3::MYH6* fusion detected by the NGS panel (Figure S1). Homozygous *CDKN2A/B* deletions were observed in 17 of 25 glioblastomas, and one case (sample #8) additionally showed homozygous *PTEN* loss on chromosome 10. In the remainder of the cohort, focal events were rare: one oligodendroglioma (sample #31) harboured a *PDGFRA* amplification, and another oligodendroglioma (sample #26) carried a homozygous *CDKN2A/B* deletion. Of diagnostic relevance, one spinal ependymoma (sample #55) exhibited a high‐level *MYCN* amplification.

### 3.2 Copy‐Number Profiling From Targeted Next‐Generation Sequencing Data

All samples were sequenced using a custom hybrid‐capture panel designed for neuropathology diagnostics (see Methods), yielding a median of 23.99 million reads (range: 5–72 million, Table S1). The majority of reads mapped off‐target (median: 17.18 million), whereas a smaller fraction represented on‐target reads (median: 8.32 million; Figure 1B). Mean on‐target coverage was 1304×, and only four of 60 samples had a mean target coverage below 500×. In all cases, more than 99% of targeted bases were covered at ≥ 50×. Notably, higher DNA quality, as reflected by increased DIN values, was significantly associated with lower off‐target rates (Pearson *r* = −0.44, *p* = 4.8 × 10^−4^; Figure S2). Leveraging both on‐ and off‐target reads (Figure 1C), we subsequently generated chromosome‐wide copy‐number profiles and incorporated custom gene annotations to highlight focal genomic alterations (Figure 1D).

**FIGURE 2:**
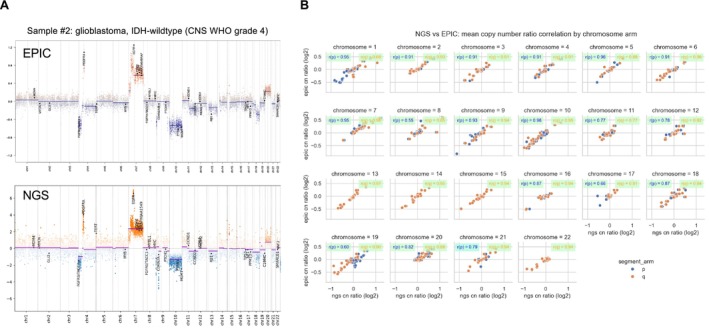
Concordance of CNV detection between targeted NGS and EPIC array platforms. (A) Representative genome‐wide comparison of copy‐number profiles generated from EPIC methylation array data (top) and targeted plus off‐target NGS reads (bottom) for sample #2 (IDH‐wildtype glioblastoma, CNS WHO grade 4). (B) Genome‐wide correlation of log_2_ copy‐number ratios obtained from EPIC and NGS data across 60 CNS tumour samples, demonstrating high concordance for both chromosomal arm‐level and focal copy‐number alterations. All correlations were statistically significant (*p* < 0.01).

We next compared copy‐number profiles generated from EPIC and NGS data across our cohort of 60 tumour samples (Figure 2). On visual inspection, chromosomal arm‐level copy‐number ratios showed high concordance (Figure 2A). Using length‐weighted mean log_2_ ratios per chromosomal arm (p and q) derived independently from CNVkit (.cnr) and EPIC/conumee (.igv) data, and classifying each arm as gain, loss or neutral based on ±0.5 log_2_ thresholds (excluding sex chromosomes), we observed a mean per‐sample arm‐level concordance of 0.95 (95%) between the two methods across all comparable arms. Similarly, correlation analysis of the log_2_ ratios derived from each method demonstrated high and significant concordance across all chromosomal arms (Figure 2B). Manual inspection confirmed a high agreement for each of the 253 chromosomal arm gains and 274 arm losses (Table S1, Figure S3). Focal events included both genes that were targeted by the panel (*EGFR*, *FGFR3*, *PDGFRA*, *CDKN2A/B*, *PTEN*, *TERT*) but also genes that were not specifically targeted by the panel (*MYCN*, *MDM4*). Across the cohort, all 19/19 focal amplifications were also detected by NGS, including 4 cases with *MDM4* amplification and one spinal ependymoma with *MYCN* amplification (sample #55). In contrast, the interpretation of homozygous deletions proved more challenging. While several cases showed clear *CDKN2A/B* homozygous deletions (samples #1, #3, #8, #9, #10, #12, #13, #17, #20, #21, #22, #23 and #26), others (samples #6, #7, #14, #15, #24 and #25) were more ambiguous with respect to their homozygous versus heterozygous status on visual inspection (Figure S3). In addition, one glioblastoma with a *PTEN* homozygous deletion detected by EPIC was not identified by NGS (sample #8; Figure S3).

### 3.3 NGS‐Based Copy‐Number Profiling for Meningioma Samples

According to the latest WHO classification of CNS tumours, *TERT* promoter mutations and homozygous *CDKN2A/B* deletions are recognised as independent criteria for upgrading meningiomas to CNS WHO grade 3, regardless of their histological features [1]. More recently, the Consortium to Inform Molecular and Practical Approaches to CNS Tumour Taxonomy (cIMPACT‐NOW) has proposed additional chromosomal arm copy‐number alterations as prognostic and/or grading markers. Specifically, co‐deletion of Chr 1p and 22q has been suggested as a molecular criterion for CNS WHO grade 2 classification, also independent of histopathological findings [9].

**FIGURE 3:**
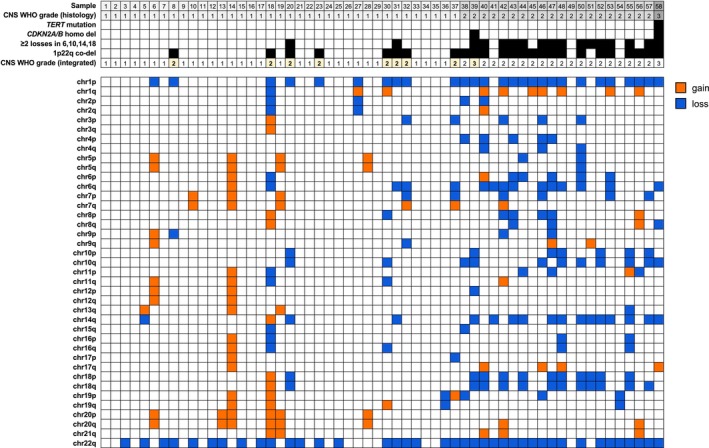
NGS‐based copy‐number analysis in meningioma samples. Genome‐wide chromosomal arm‐level copy‐number profiles of 58 meningioma samples stratified by histological CNS WHO grade. Tumours of higher grade exhibited an increased CNV burden. Co‐deletion of chromosomes 1p and 22q was observed in a subset of CNS WHO grade 1 tumours, resulting in an upgrade of the integrated CNS WHO grade (yellow boxes), and in the majority of CNS WHO grade 2 tumours. Additional whole‐arm losses involving chromosomes 6, 10, 14 and 18 were enriched in higher‐grade tumours.

To investigate these molecular features, we used off‐target sequencing reads from 58 meningioma samples (37 CNS WHO grade 1, 20 CNS WHO grade 2, and 1 CNS WHO grade 3) using our small‐scale NGS panel to perform genome‐wide copy‐number profiling. Sequencing yielded a median of 24.41 million reads (range, 7.8–110.4 million reads, Table S2). The mean number of chromosomal arm copy‐number alterations was 3.46 for CNS WHO grade 1, 8.05 for CNS WHO grade 2 and 7 for the single CNS WHO grade 3 tumour (Figure 3, Table S2). The CNS WHO grade 3 meningioma harboured a *TERT* promoter mutation (C250T) and homozygous *CDKN2A/B* deletion, whereas all other cases were *TERT* wildtype. Notably, one tumour initially classified as CNS WHO grade 2 by histology demonstrated a homozygous *CDKN2A/B* deletion, resulting in its reclassification as CNS WHO grade 3.

Co‐deletion of chromosomes 1p and 22q was identified in eight of 37 (22%) tumours with CNS WHO grade 1 morphology, supporting their reclassification to CNS WHO grade 2 according to the proposed molecular criteria [9]. Notably, 6 of these 8 meningiomas exhibited focal areas of increased cellular density and proliferative activity, whereas 2 tumours showed no atypical features and had low proliferation indices (1% and 3%, respectively). Sixteen of 20 (80%) CNS WHO grade 2 meningiomas exhibited 1p and 22q deletions. Two tumours with CNS WHO grade 1 morphology and 1p22q co‐deletion additionally showed ≥ 2 whole‐arm chromosomal losses involving chromosomes 6, 10, 14 and 18, a pattern associated with an increased risk of recurrence despite low histological grade [9]. These chromosomal alterations were observed in 14/20 (70%) of grade 2 meningiomas and in the CNS WHO grade 3 meningioma (Figure 3). We validated our NGS‐based copy‐number analysis using EPIC methylation arrays in 11 of 58 cases, including the CNS WHO grade 2 meningioma that was upgraded due to a homozygous *CDKN2A/B* deletion (sample #39); all validated cases showed concordant results (Table S2).

### 3.4 Focal Copy‐Number Alterations as Indicators of Gene Fusions

In our evaluation cohort, focal *FGFR3* amplification served as an indicator of an *FGFR3::MYH6* fusion, which was subsequently confirmed by fusion calling. We further analysed six pilocytic astrocytomas, five of which harboured *KIAA1549::BRAF* fusions and one that exhibited a *FAM131B::BRAF* fusion. *KIAA1549::BRAF* fusions typically result from small duplications on Chr 7q34, whereas *FAM131B::BRAF* fusions are associated with focal deletions on Chr 7q that are also detectable upon CNV calling from DNA methylation array data [2].

**FIGURE 4:**
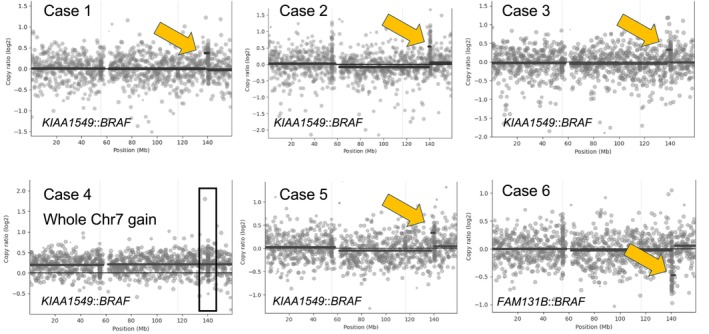
Focal copy‐number alterations in *BRAF*‐fused pilocytic astrocytomas. Copy‐number profiles of chromosome 7 for six pilocytic astrocytoma cases harbouring *BRAF* fusions. Arrows highlight focal copy‐number changes at the *BRAF* fusion locus. Case 4 shows a gain of the entire chromosome 7 rather than a focal alteration. The corresponding fusion partners are indicated below each panel (*KIAA1549::BRAF* or *FAM131B::BRAF*).

The six samples were sequenced at a median depth of 16.85 million reads (Table S3). Four of the five tumours with the characteristic *KIAA1549::BRAF* fusion showed focal copy‐number gains on Chr 7q (Figure 4), whereas Case 4 demonstrated gain of the entire Chr 7 without evidence of focal copy‐number alteration. As expected, the pilocytic astrocytoma harbouring the *FAM131B::BRAF* fusion displayed a focal loss on Chr 7q (Figure 4).

## 4 Discussion

Next‐generation sequencing has become a standard approach for detecting mutations and gene fusions in diagnostic neuropathology [12, 13, 19]. The vast majority of NGS panels are hybrid‐capture‐based, enriching predefined genomic regions using targeted oligonucleotides. However, this approach inevitably generates off‐target reads due to non‐specific probe hybridization, which are typically discarded during standard bioinformatic pipelines. Here, we show that these off‐target reads from a minimal (< 0.2 Mb) custom NGS panel, solely designed for mutation and fusion detection without tiling probes specifically designed for CNV calling [11], can reliably generate genome‐wide CNV profiles in CNS tumour samples. Although bioinformatic tools for copy‐number detection from targeted NGS data initially focused on depth of coverage in on‐target regions [20], methods that incorporate off‐target reads, such as cnvOffSeq [21], CopywriteR [16] and CNVkit [17] have long been available, yet they have not been systematically evaluated for copy‐number profiling in diagnostic (neuro)pathology.

At the chromosomal arm level, these profiles achieved near‐perfect concordance with methylation array‐derived CNVs across key diagnostic entities, such as the +7/−10 signature in IDH‐wildtype glioblastoma and 1p/19q codeletion in oligodendroglioma [1]. However, in tumours with marked deviations from the normal diploid state, particularly choroid plexus tumours, interpretation of relative copy‐number variations may be more challenging, as absolute ploidy changes shift the baseline against which gains and losses are assessed. Focal, high‐level amplifications were fully recapitulated (19/19), encompassing *EGFR*, *MDM4* and *MYCN* alterations with immediate diagnostic or prognostic relevance in glioblastoma and ependymoma [1, 6]. Notably, whereas *EGFR* is intentionally enriched (on‐target) by the panel, *MDM4* and *MYCN* were not specifically targeted, with all reads supporting their amplification detection derived exclusively from off‐target sequences. In contrast, interpretation of homozygous *CDKN2A/B* deletions proved more challenging, with ambiguous deletion status in 6 of 19 cases. This limitation is not unique to our approach, as commonly used tools such as conumee2 applied to DNA methylation array data show a sensitivity of only 60.9% when compared with SNP arrays [18]. Accordingly, additional confirmatory testing may be required for unresolved cases, particularly in samples with low tumour cell content. Another discordant finding was a *PTEN* homozygous deletion detected by EPIC in one glioblastoma that was not identified by NGS, despite complete on‐target coverage of all *PTEN* exons.

Extending this approach to meningiomas further underscores its clinical value: we detected *CDKN2A/B* homozygous deletions and codeletions of 1p and 22q, facilitating molecular upgrading in 16% of histologically lower grade tumours, in line with recent cIMPACT‐NOW recommendations and WHO classification updates [9, 22]. These alterations, along with additional chromosomal losses (e.g., 6, 10, 14 and 18), are associated with increased recurrence risk and poorer prognosis [9], supporting risk stratification beyond histology alone. Our findings align with methylation‐based studies showing that CNV burden correlates with meningioma aggressiveness [23], while showing that off‐target NGS can deliver comparable insights concurrently with on‐target data on diagnostically relevant *TERT* promoter mutations. However, a limitation of the present approach is that it remains unclear to what extent very small or low‐level chromosomal arm alterations (e.g., < 10% of an arm), such as those described for 1p loss in meningiomas, can be detected, particularly in the setting of variable tumour purity and ploidy.

Focal copy‐number events may act as indicative genomic patterns suggestive of underlying gene fusions and should prompt gene fusion analysis. Examples include 7q34 duplications indicative of *BRAF*‐rearranged low‐grade gliomas [2, 24], chromothripsis associated with *ZFTA*‐fused ependymomas [25] and *FGFR3* focal gains implicated in *FGFR3::TACC3* fusions [26].

In summary, our findings show that copy‐number information can be extracted from diagnostic NGS panel sequencing data as an additional, easily implementable tool for genome‐wide CNV assessment in CNS tumours. By leveraging sequencing data that are already generated, this approach can be integrated into existing diagnostic workflows without additional costs and can shorten the time to an integrated diagnosis, particularly when characteristic patterns such as 1p/19q codeletion or +7/−10 are readily apparent.

## Funding

The authors have nothing to report.

## Conflicts of Interest

The authors declare no conflicts of interest.

## Supporting information


**Figure S1:** nan70070‐sup‐0001‐Figure_S1.pdf. ***FGFR3::MYH6* fusion in a glioblastoma with *FGFR3* amplification.** A, CNV profile showing *FGFR3* amplification. B, Fusion analysis with arriba revealed a *FGFR3::MYH6* fusion with 119 split and 87 discordant reads.

**Figure S2:** nan70070‐sup‐0002‐Figure_S2.pdf. **Correlation of DIN values and off‐target reads.** Correlation analysis shows a relationship between higher DIN values and lower number of off‐target reads (r = −0.44, *p* = 4.8 x 10^−4^).

**Figure S3:** Comparative visualisation of genome‐wide copy‐number profiles derived from DNA methylation arrays and next‐generation sequencing for all 60 analysed cases.

**Table S1:** Neuropathological, genetic and epigenetic characteristics of the 60 CNS tumour samples.

**Table S2:** Neuropathological and genetic characteristics from 58 meningioma samples.

**Table S3:** Neuropathological and genetic characteristics of 6 pilocytic astrocytomas.

**Table S4:** Technical specifications and design features of the different versions of the custom hybrid‐capture next‐generation sequencing panel.

## Data Availability

The data that support the findings of this study are available in the Supporting Information of this article.
