# Supplementary material for: Detection of Copy‐Number Variations in CNS Tumours From Off‐Target Reads of Hybrid‐Capture Sequencing
Source: Neuropathol Appl Neurobiol. 2026 Mar 16;52(2):e70070. doi: 10.1111/nan.70070 (PMC12989910; doi:10.1111/nan.70070)
Supplement: Supplementary file 1 — Figure S1: nan70070‐sup‐0001‐Figure_S1.pdf. FGFR3::MYH6 fusion in a glioblastoma with FGFR3 amplification. A, CNV profile showing FGFR3 amplification. B, Fusion analysis with arriba revealed a FGFR3::MYH6 fusion with 119 split and 87 discordant reads. [file NAN-52-e70070-s007.pdf]

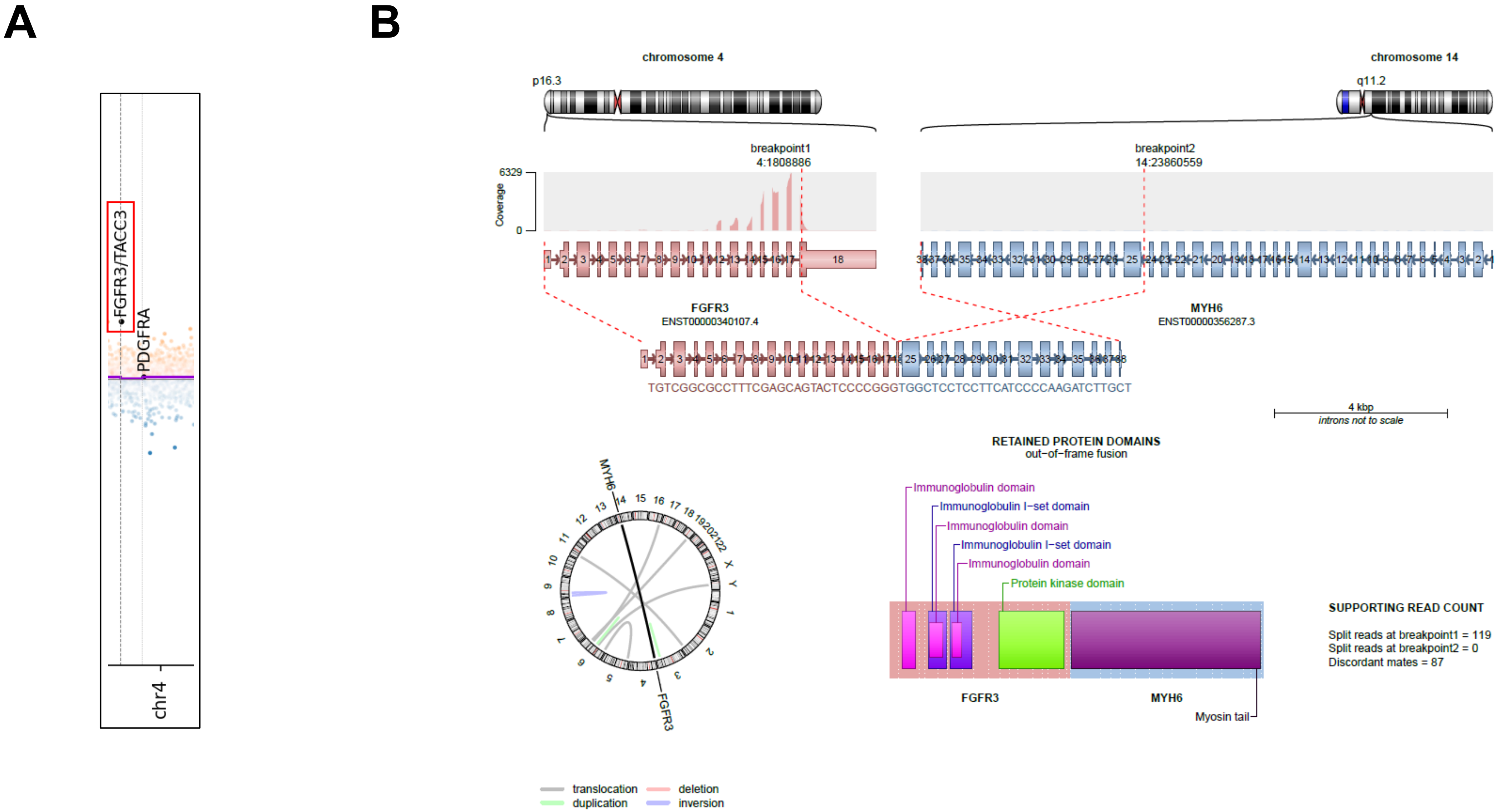

Supplementary Figure 1: *FGFR3::MYH6* fusion in a glioblastoma with *FGFR3* amplification. A, CNV profile showing *FGFR3* amplification. B, Fusion analysis with arriba revealed a *FGFR3::MYH6* fusion with 119 split and 87 discordant reads.
