# Supplementary material for: Detection of Copy‐Number Variations in CNS Tumours From Off‐Target Reads of Hybrid‐Capture Sequencing
Source: Neuropathol Appl Neurobiol. 2026 Mar 16;52(2):e70070. doi: 10.1111/nan.70070 (PMC12989910; doi:10.1111/nan.70070)
Supplement: Supplementary file 2 — Figure S2: nan70070‐sup‐0002‐Figure_S2.pdf. Correlation of DIN values and off‐target reads. Correlation analysis shows a relationship between higher DIN values and lower number of off‐target reads (r = −0.44, p = 4.8 x 10^−4^). [file NAN-52-e70070-s003.pdf]

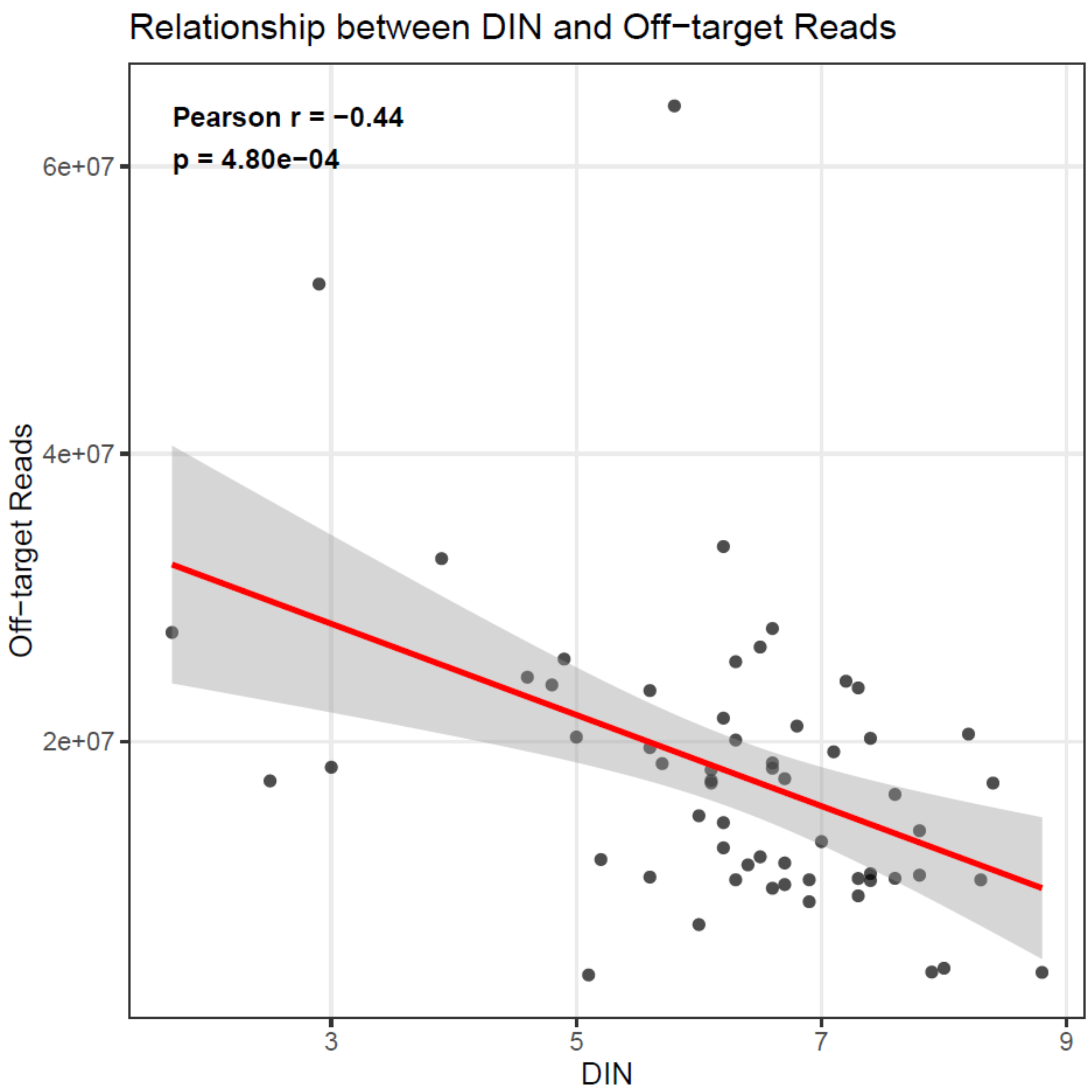

Supplementary Figure 2: Correlation of DIN values and off-target reads. Correlation analysis shows a relationship between higher DIN values and lower number of off-target reads ($r = -0.44$, $p = 4.8 \times 10^{-4}$).
