# Supplementary material for: Detection of Copy‐Number Variations in CNS Tumours From Off‐Target Reads of Hybrid‐Capture Sequencing
Source: Neuropathol Appl Neurobiol. 2026 Mar 16;52(2):e70070. doi: 10.1111/nan.70070 (PMC12989910; doi:10.1111/nan.70070)

sample #1

- Gains: 3p, 3q, 7p, 7q, 8p, 8q, 20p, 20q
- Losses: 10p, 10q
- Focal CNVs: Del *CDKN2A/B*

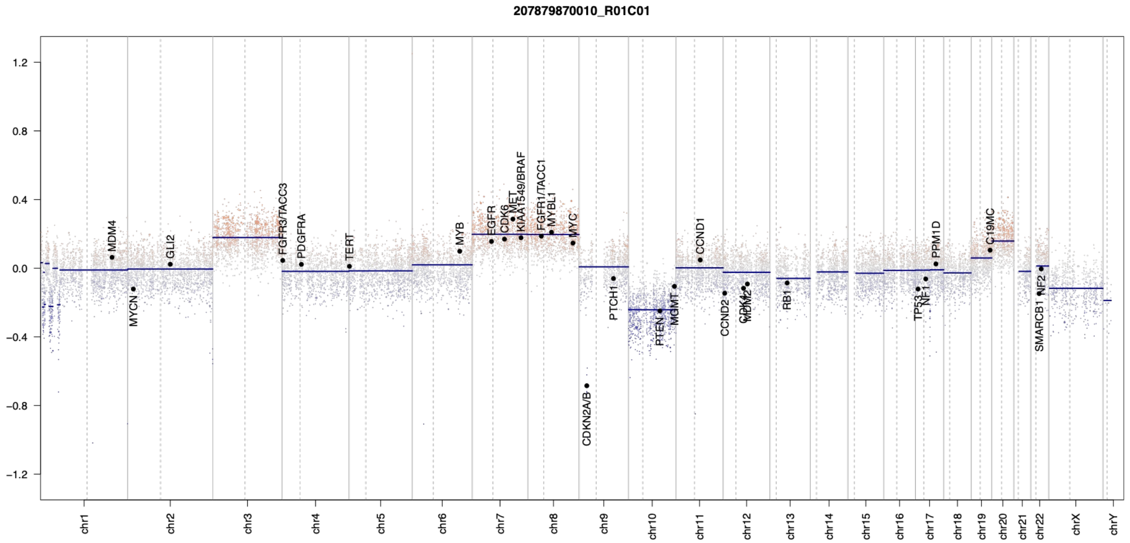

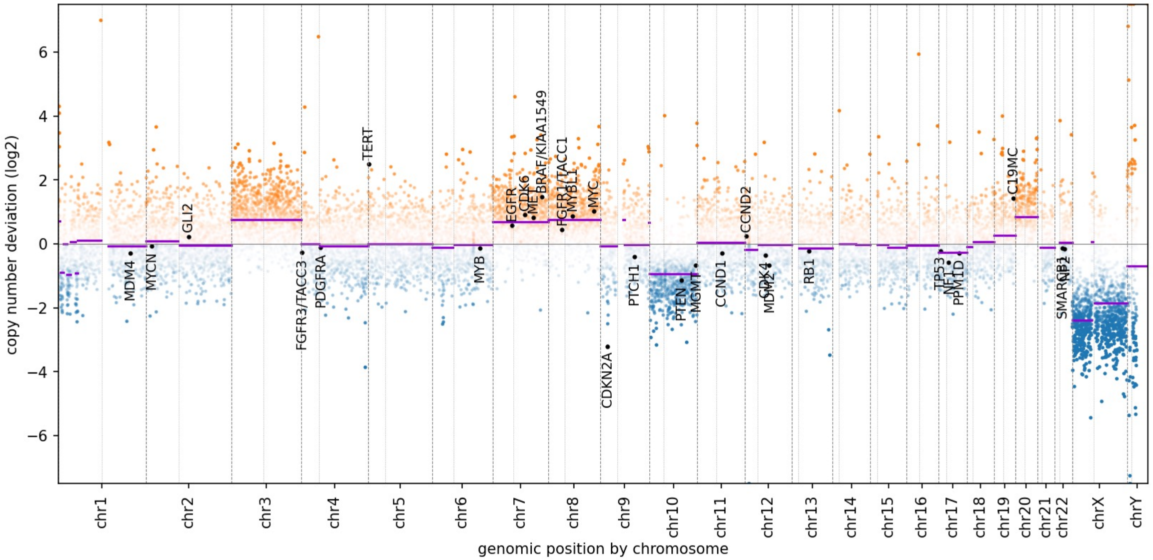

sample #2

- Gains: 7p, 7q, 20p, 20q
- Losses: 4p, 10p, 10q, 18q
- Focal CNVs: Amp *PDGFRA, EGFR*

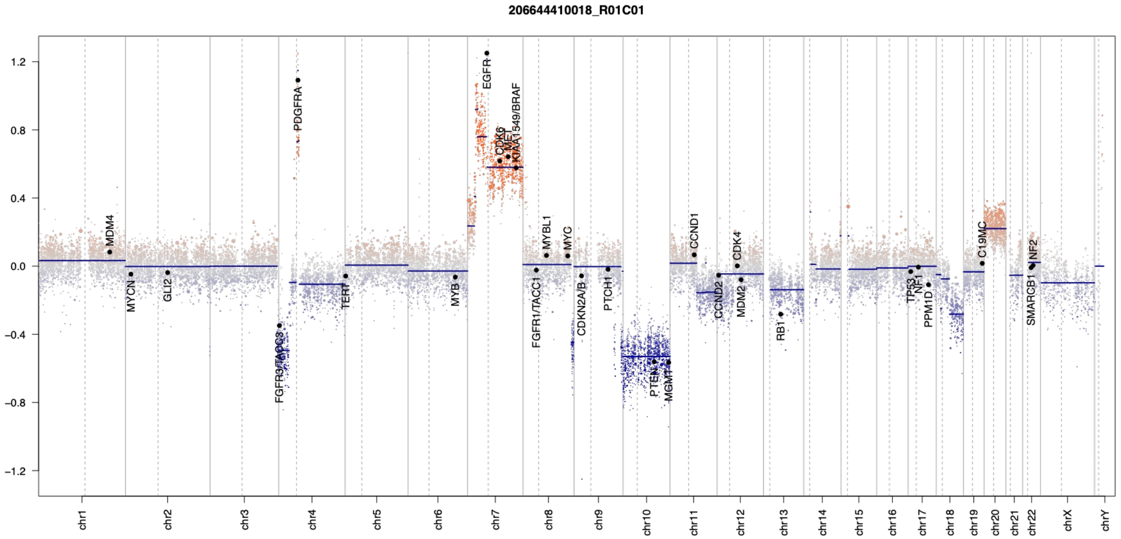

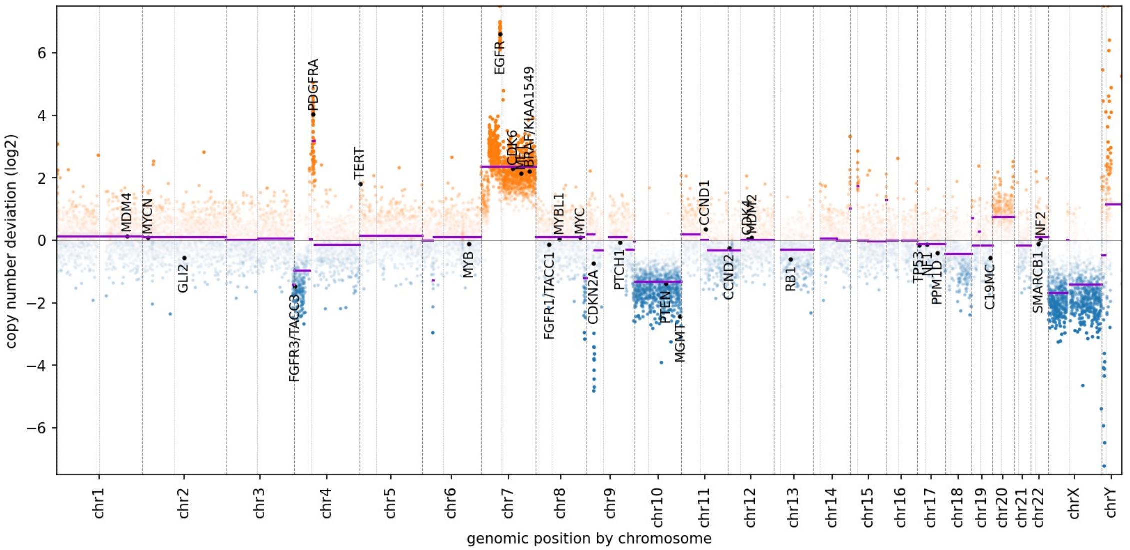

sample #3

•Gains: 7p, 7q, 12p, 12q, 20p, 20q, 21q

•Losses: 6p, 6q, 8p, 8q, 9p, 9q, 10p, 10q, 14q, 15q, 16p, 16q, 18p, 18q, 19p, 19q, 22q

•Focal CNVs: Amp *MDM4*; Del *CDKN2A/B*

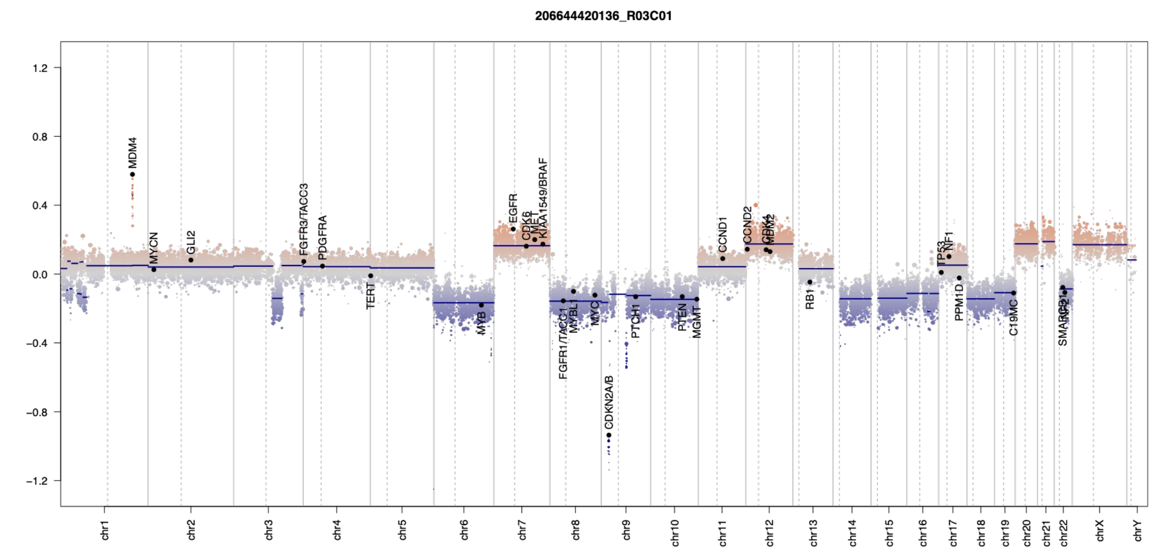

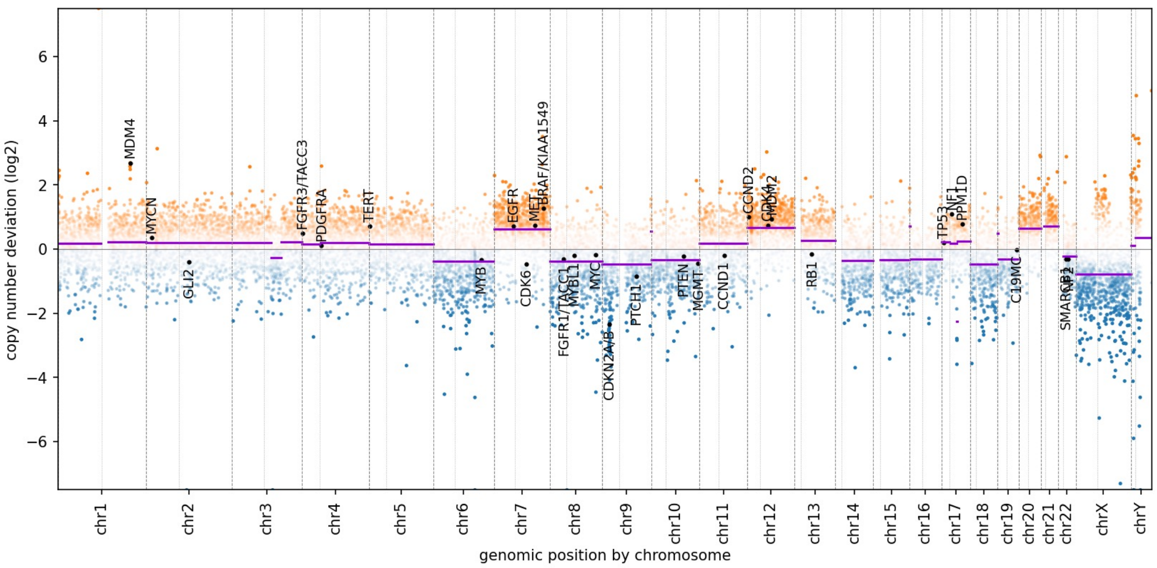

sample #4

•Gains: 7p, 7q, 19p, 19q

•Losses: 10p, 10q, 22q

•Focal CNVs: Amp *FGFR3*

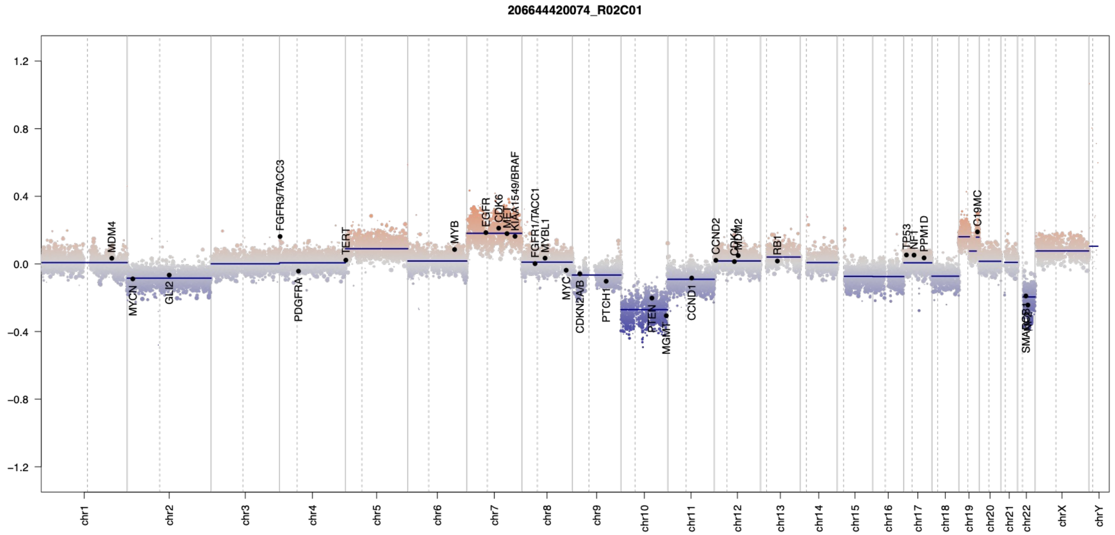

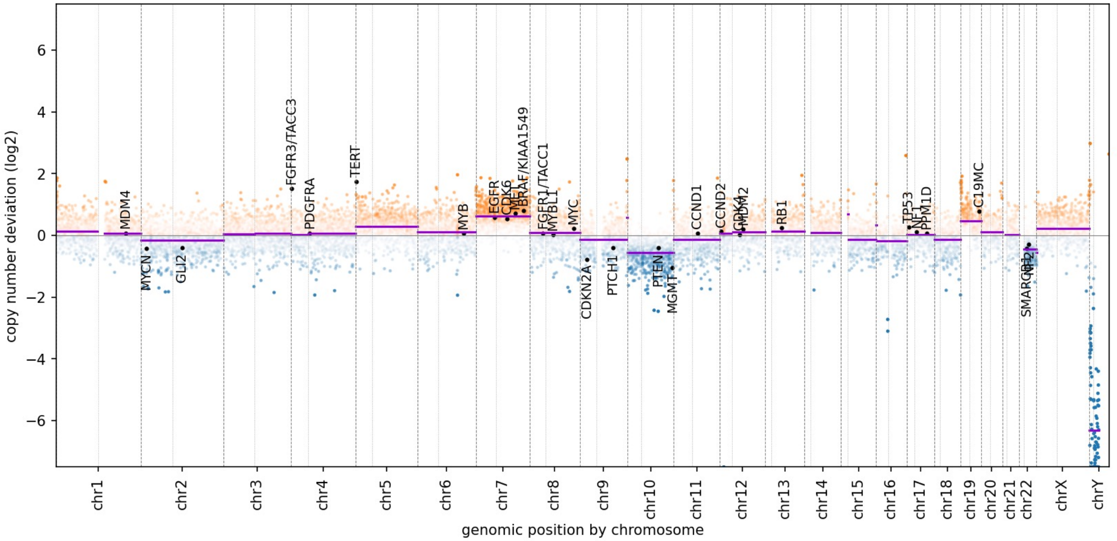

sample #5

- Gains: 7p, 7q, 18q
- Losses: 6p, 6q, 9p, 10p, 10q, 12p, 13q, 15q
- Focal CNVs: –

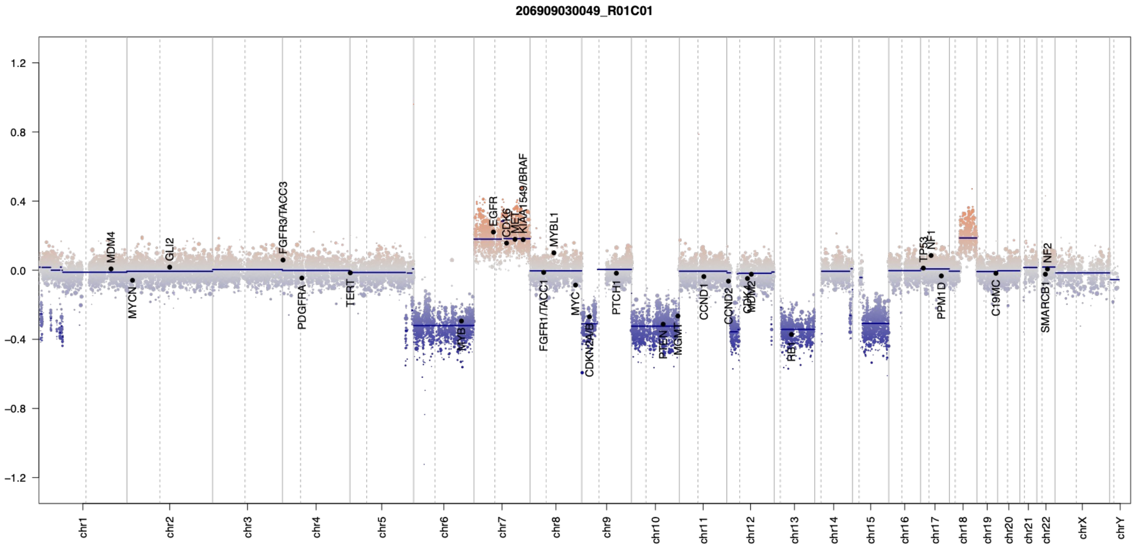

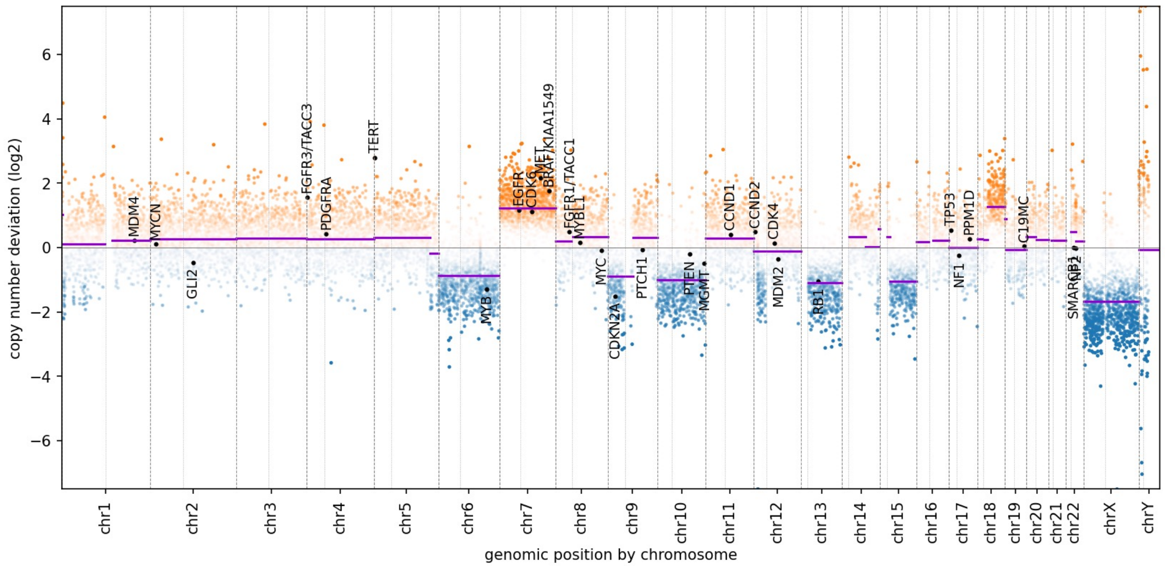

sample #6

•Gains: 7p, 7q, 19p, 19q

•Losses: 10p, 10q, 15q

•Focal CNVs: Amp *MDM4*, *EGFR*

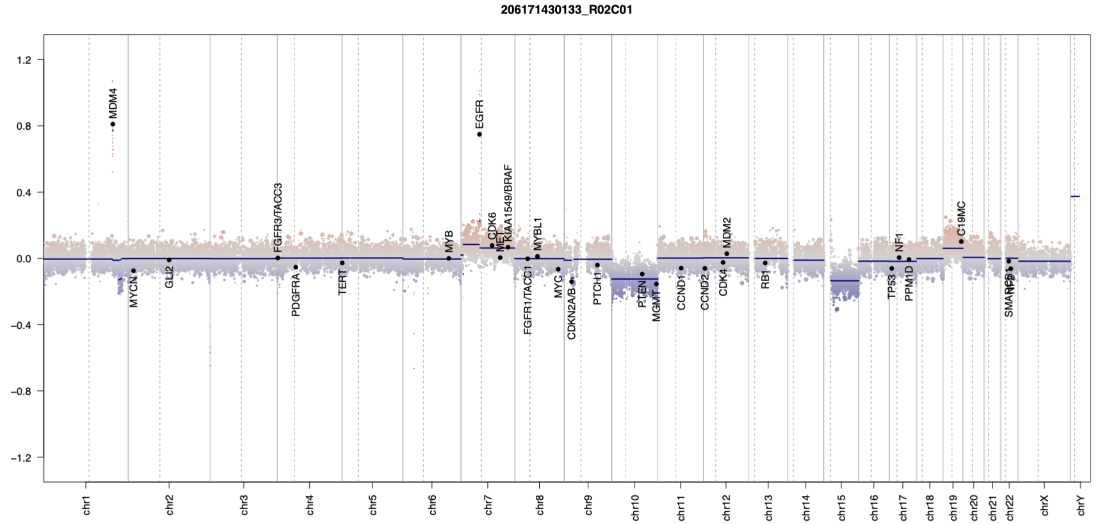

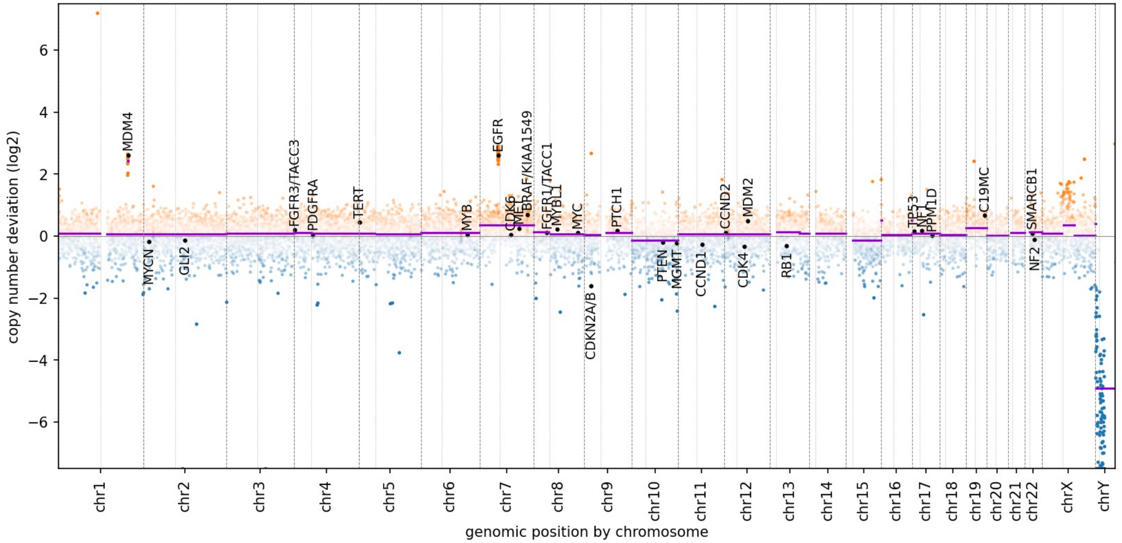

sample #7

- Gains: 7p, 7q, 20p, 20q
- Losses: 10p, 10q
- Focal CNVs: Del *CDKN2A/B*

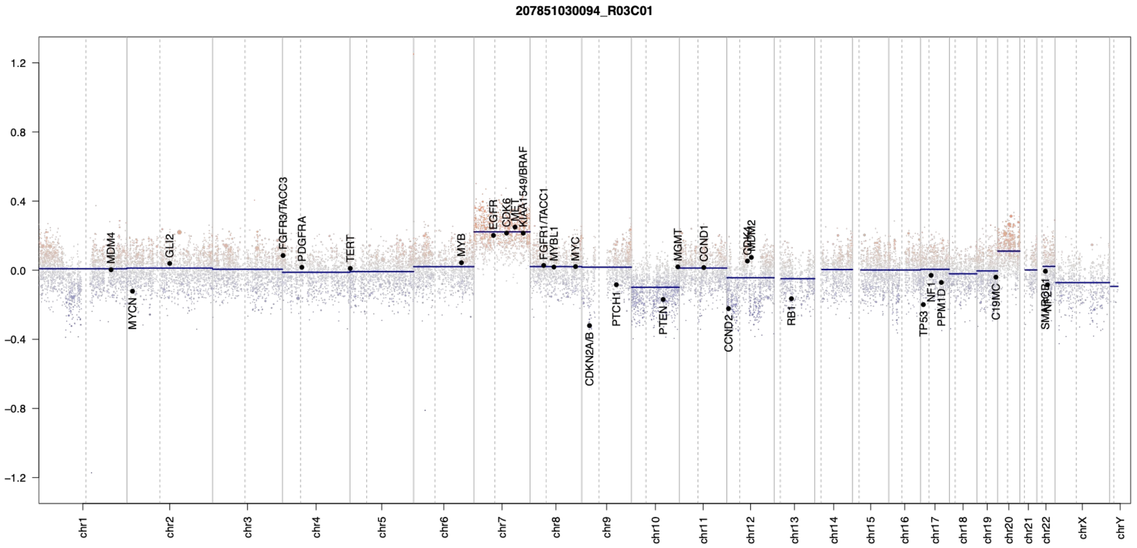

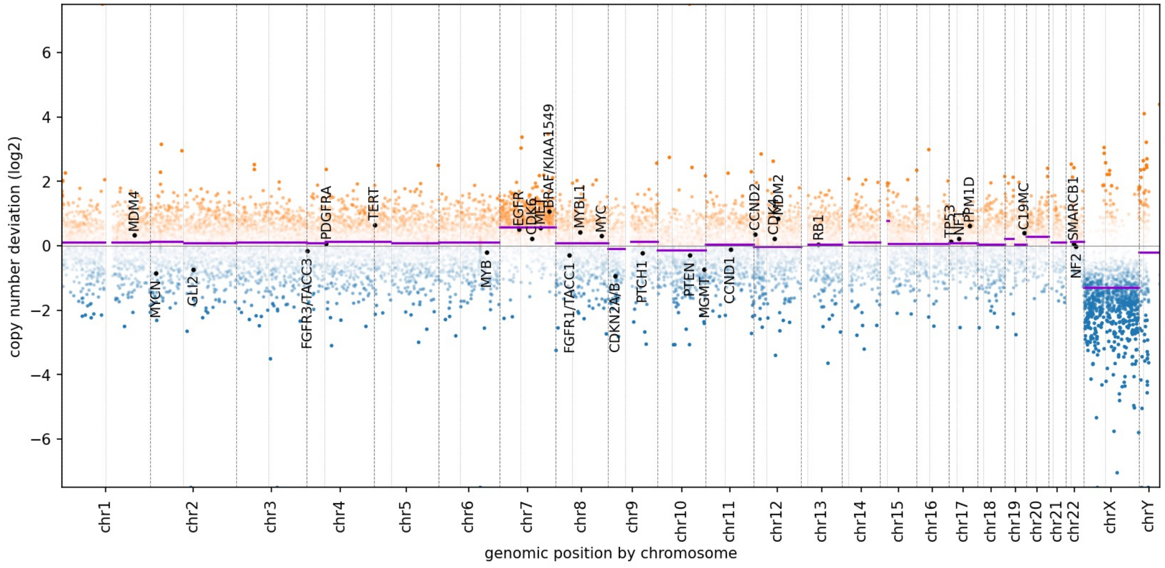

sample #8

- Gains: 1q, 7p, 7q
- Losses: 3q, 9p, 10p, 10q, 15q
- Focal CNVs: Del *CDKN2A/B*, Del *PTEN*

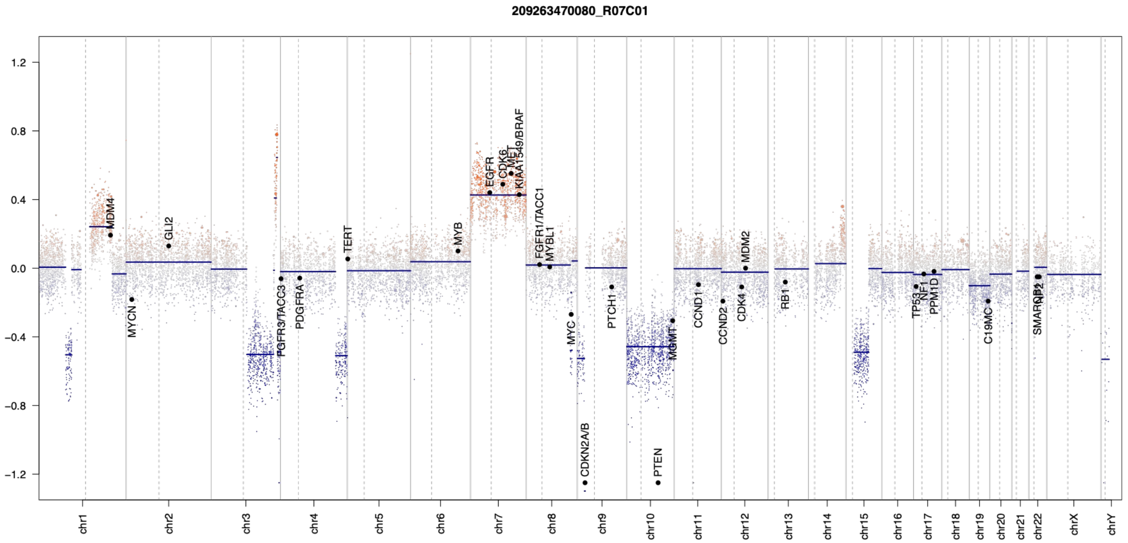

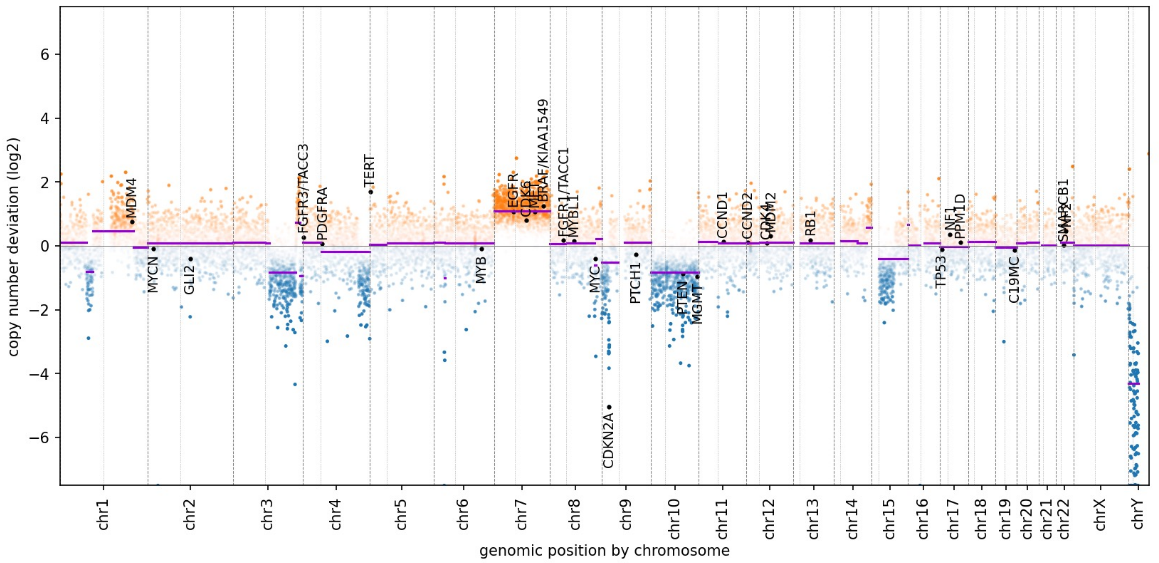

sample #9

- Gains: 7p, 7q
- Losses: 6p, 6q, 9p, 9q, 10p, 10q, 12p
- Focal CNVs: Amp *EGFR*, Del *CDKN2A/B*

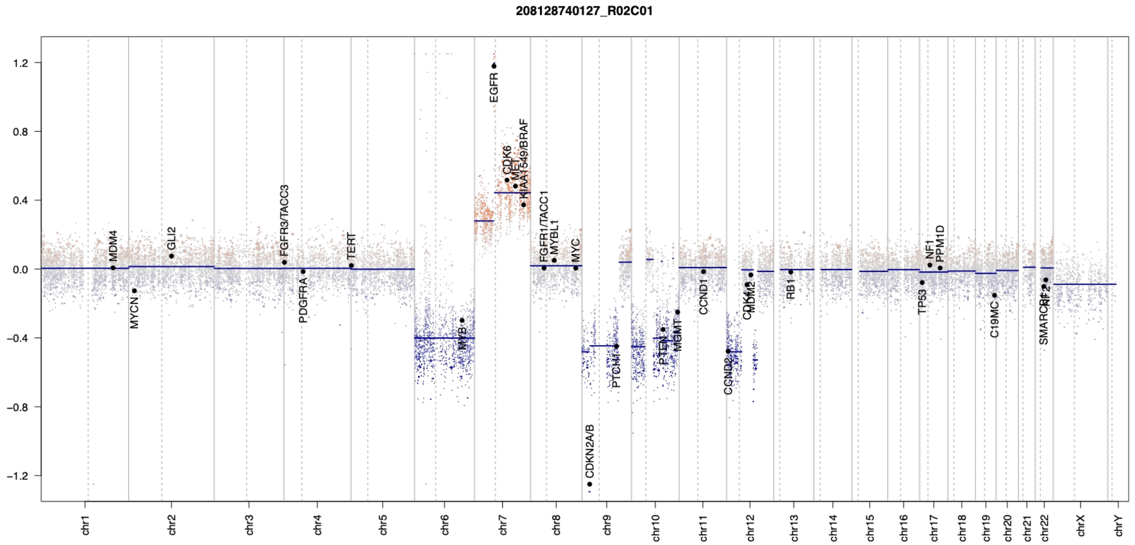

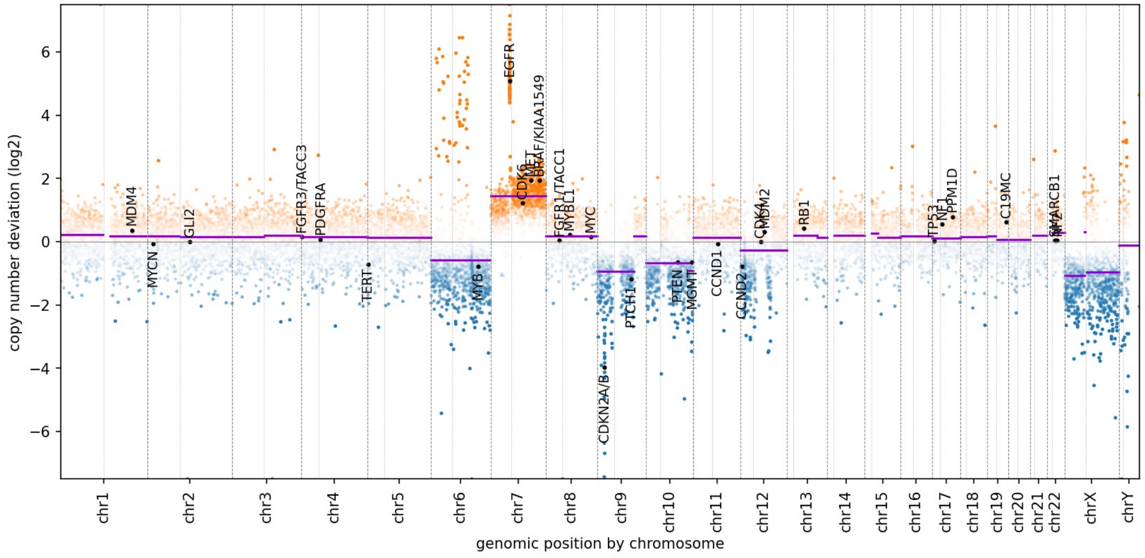

sample #10

- Gains: 7q, 18p, 20p, 20q
- Losses: 3p, 3q, 4q, 5p, 9p, 10p, 10q, 13q, 14q, 18q, 21q, 22q
- Focal CNVs: Del *CDKN2A/B*

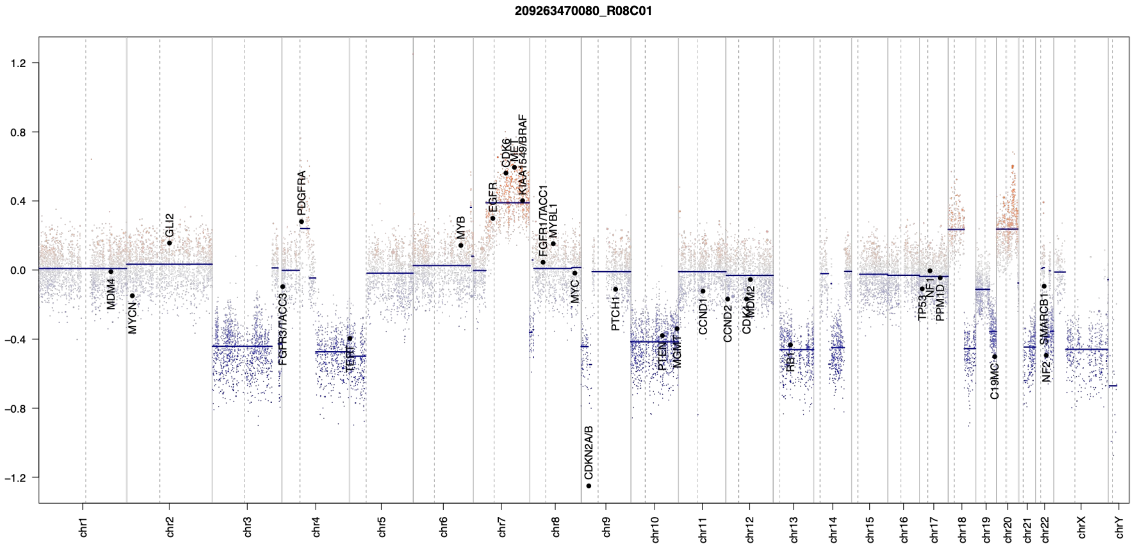

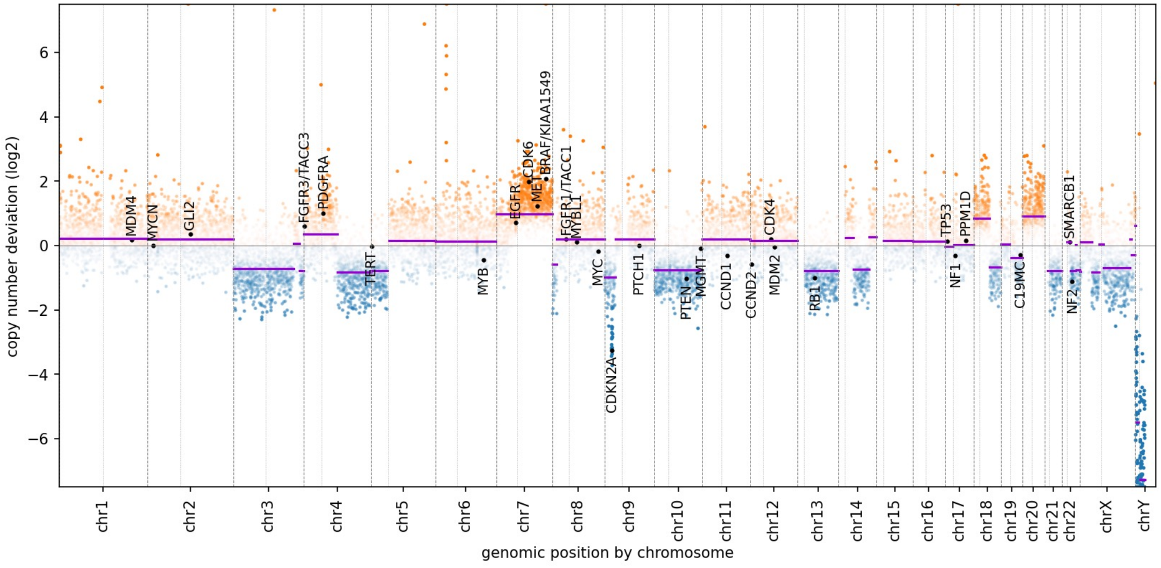

sample #11

- Gains: 1p, 1q, 2p, 2q, 7p, 7q, 19p, 19q
- Losses: 10p, 10q
- Focal CNVs: Amp *MDM4*, *EGFR*

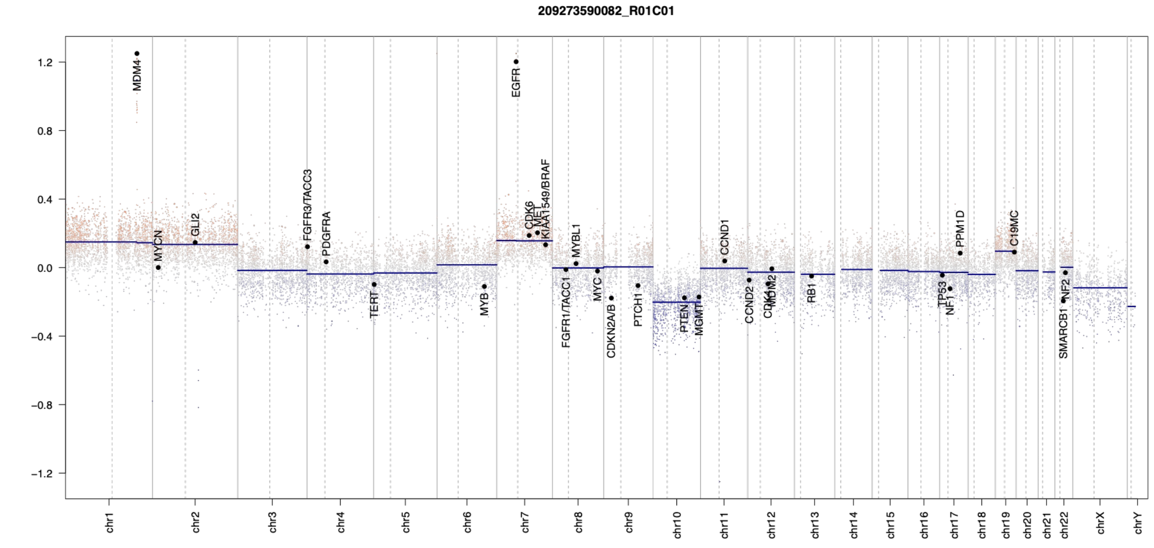

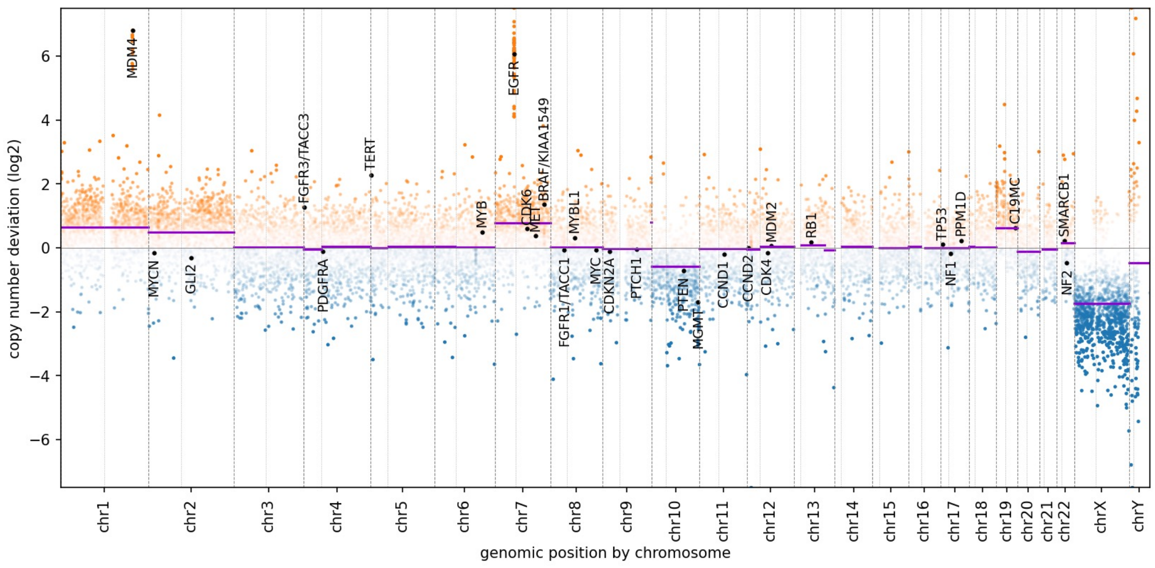

sample #12

- Gains: 7p, 7q, 19p, 19q, 20p, 20q
- Losses: 9p, 10p, 10q
- Focal CNVs: Del *CDKN2A/B*; Amp *EGFR*

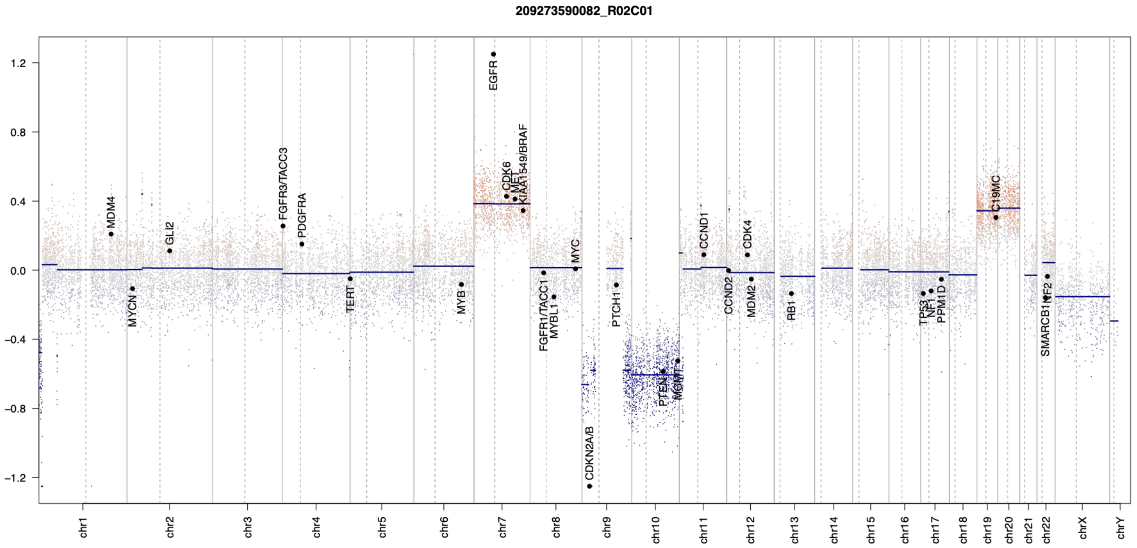

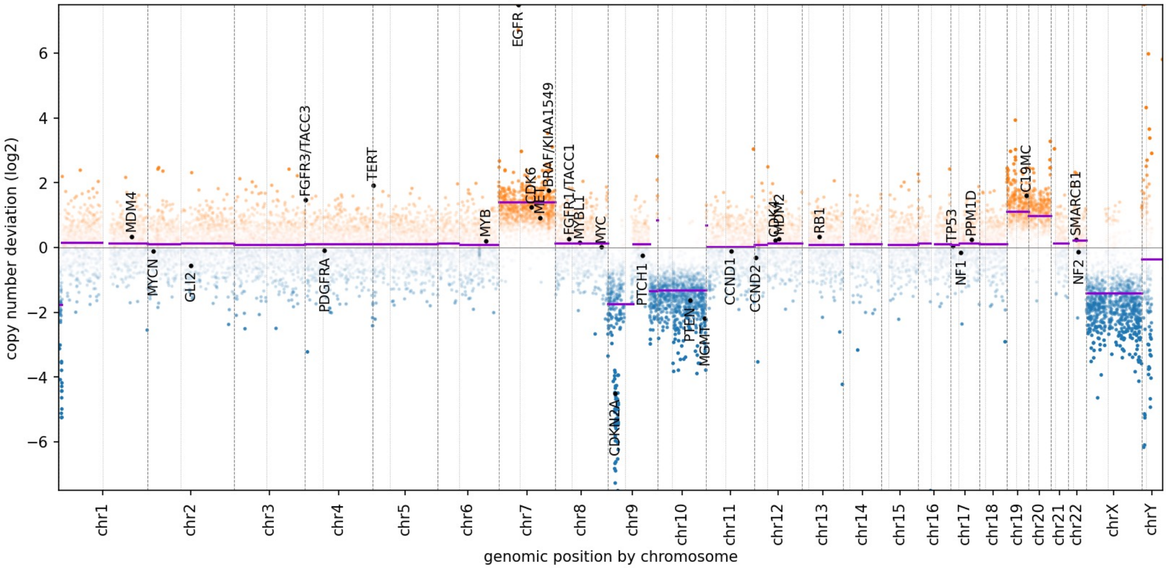

sample #13

- Gains: 7p, 7q, 14q, 20p, 20q
- Losses: 10p, 10q
- Focal CNVs: Del *CDKN2A/B*; Amp *EGFR*

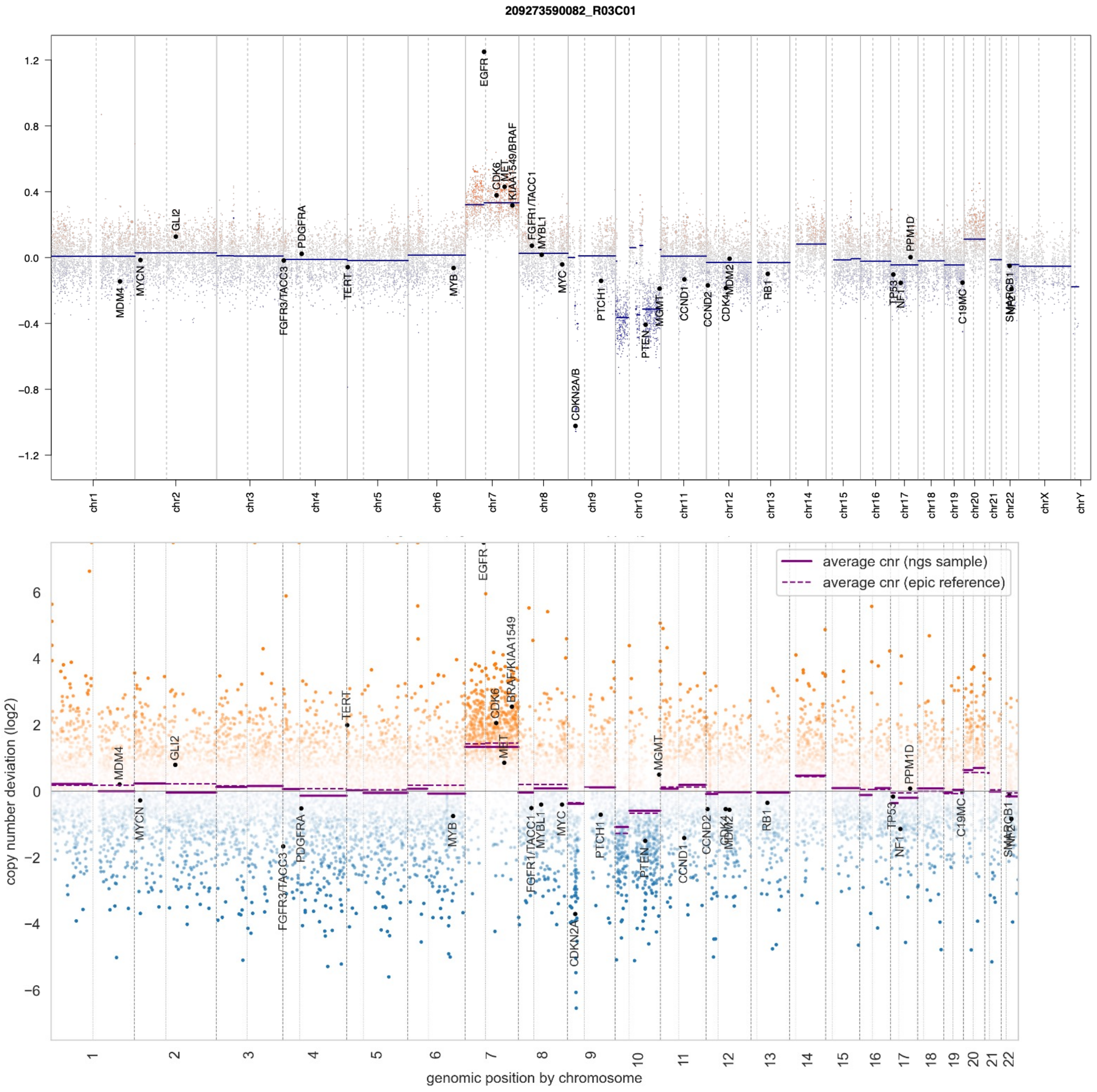

sample #14

- Gains: 7p, 7q
- Losses: 1q, 6q, 9p, 10p, 10q, 13q, 14q
- Focal CNVs: Del *CDKN2A/B*; Amp *MDM4*

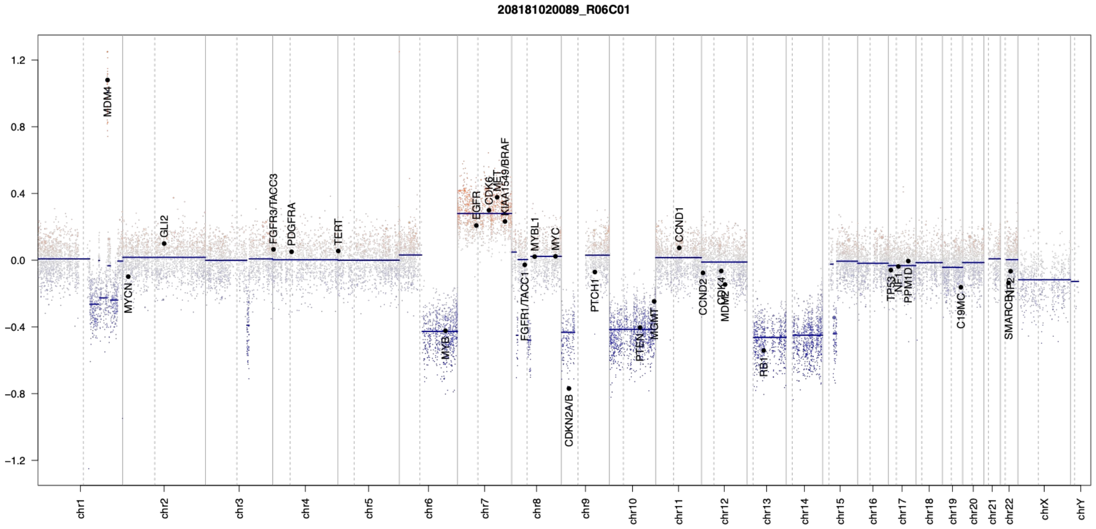

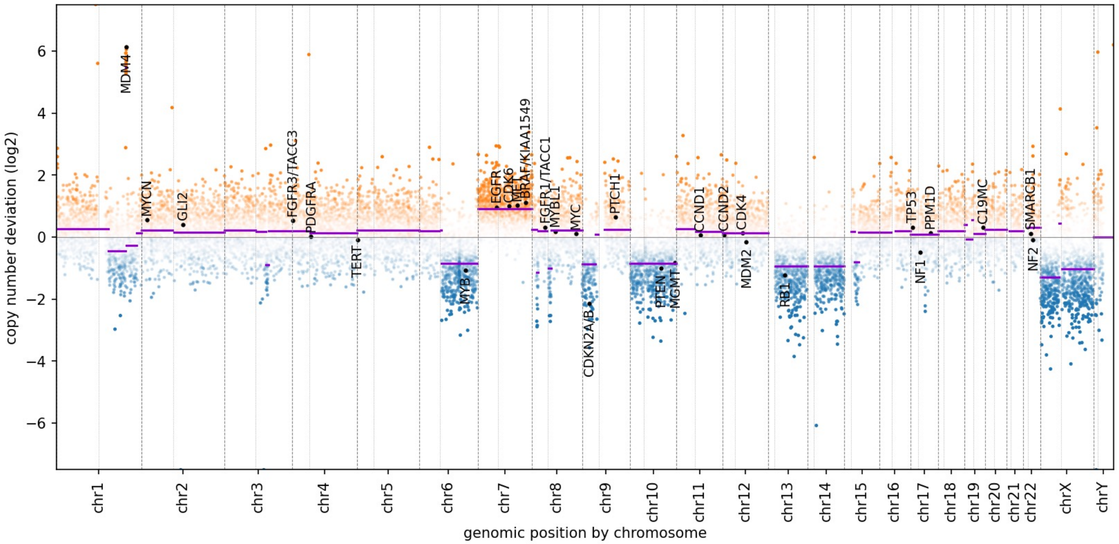

sample #15

- Gains: 7p, 7q
- Losses: 6q, 9p, 9q, 10p, 10q, 14q, 16q
- Focal CNVs: -

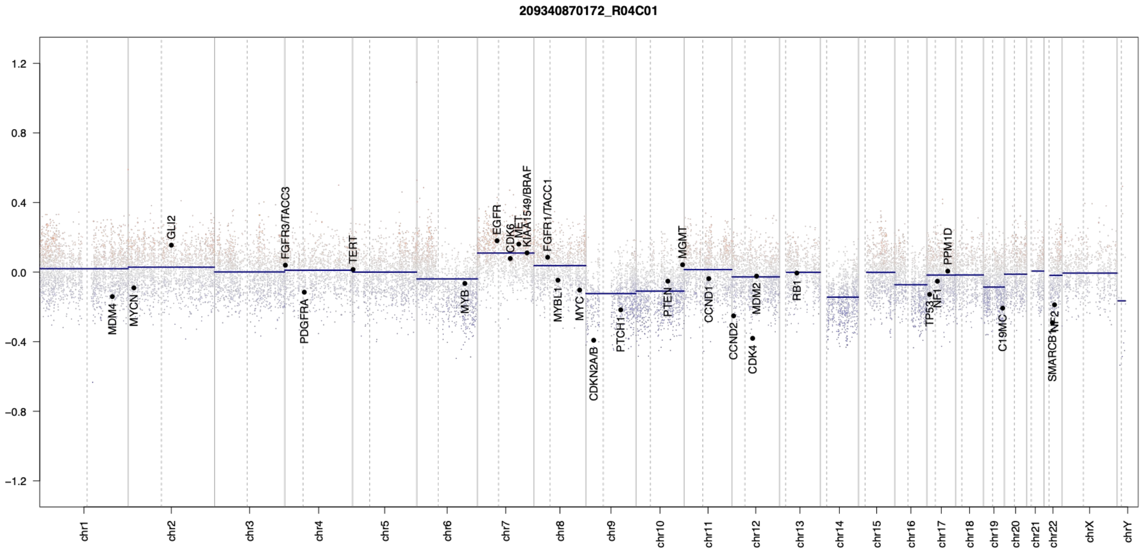

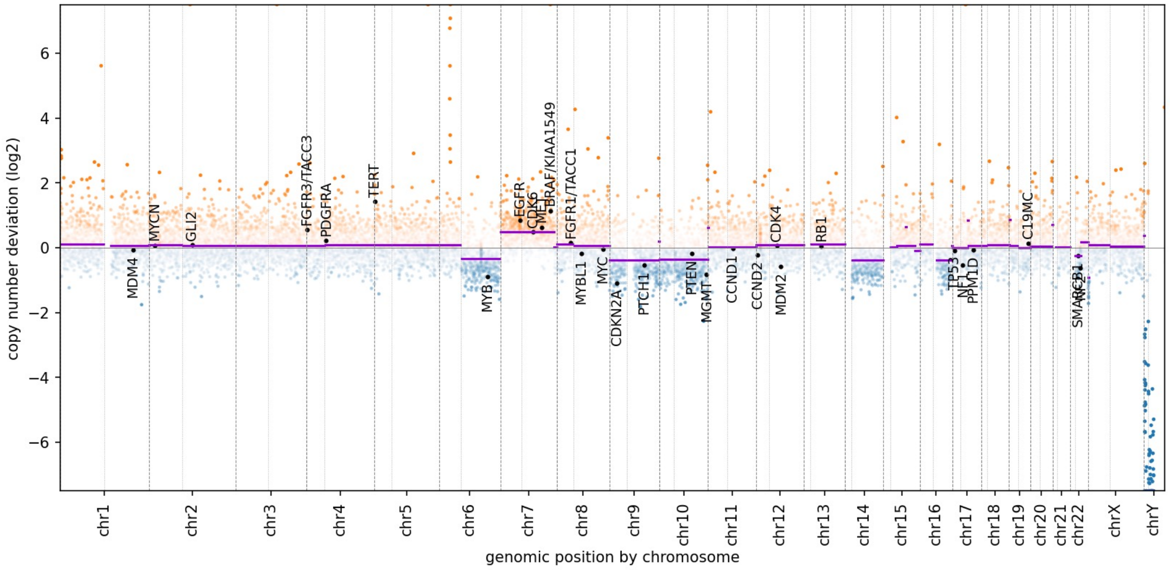

sample #16

- Gains: 7q
- Losses: 10p, 10q
- Focal CNVs: Amp *EGFR*

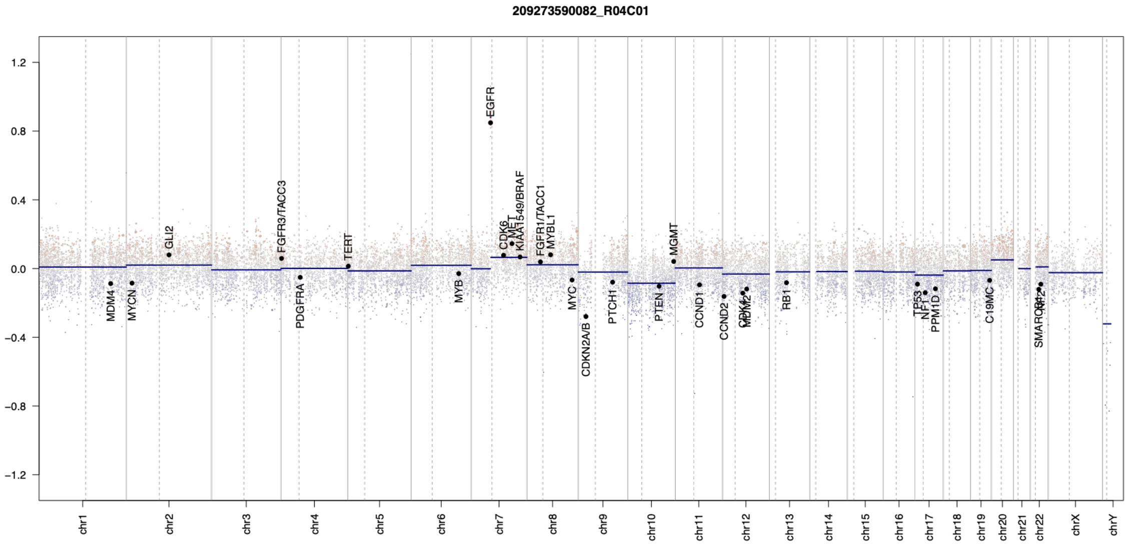

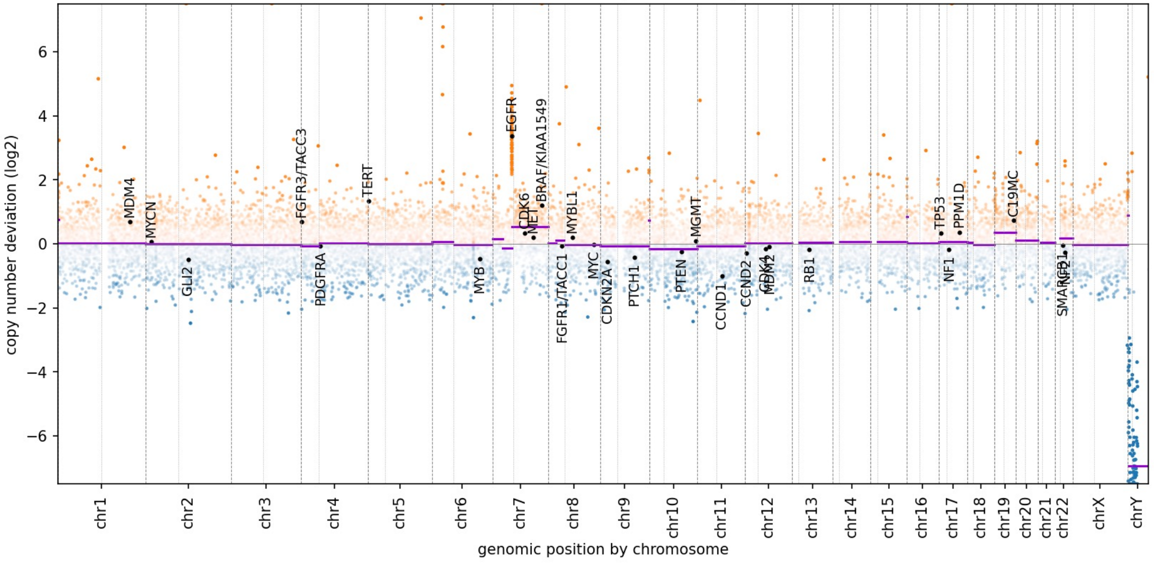

sample #17

- Gains: 7p, 7q, 19p
- Losses: 10p, 10q
- Focal CNVs: Del *CDKN2A/B*; Amp *EGFR*

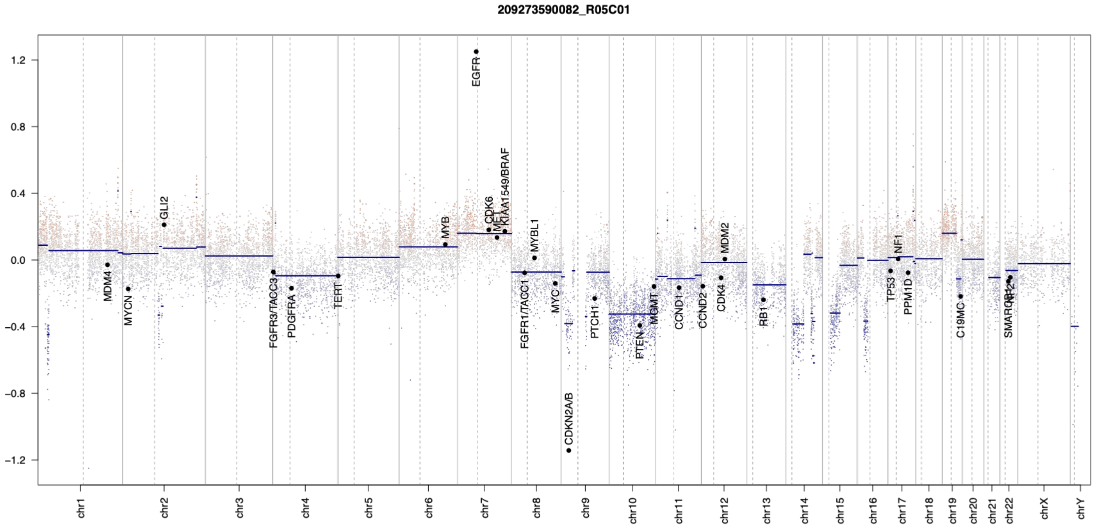

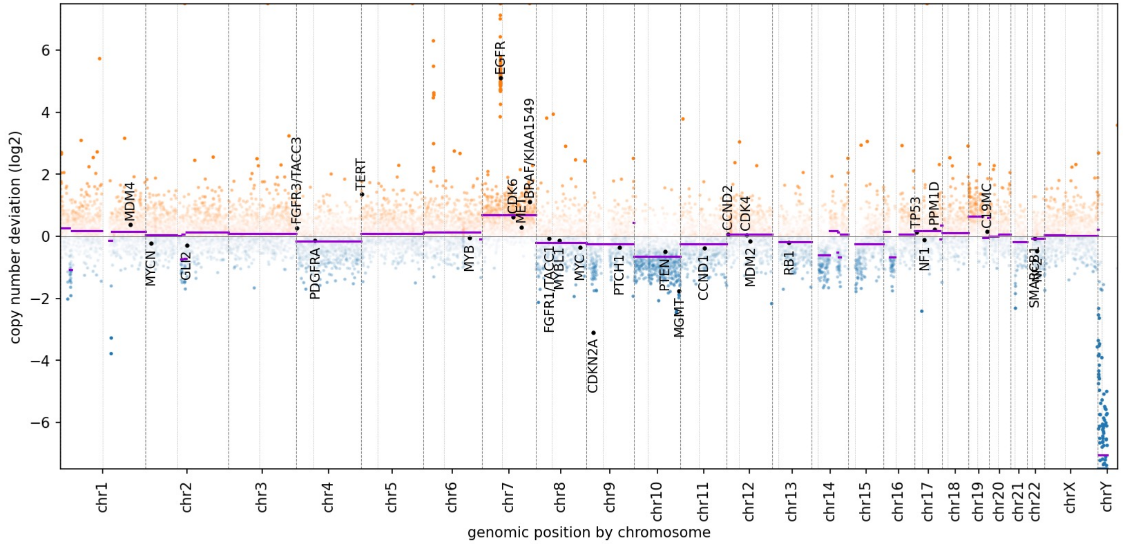

sample #18

- Gains: 7p, 7q
- Losses: 9p, 9q, 10p, 10q, 13q
- Focal CNVs: -

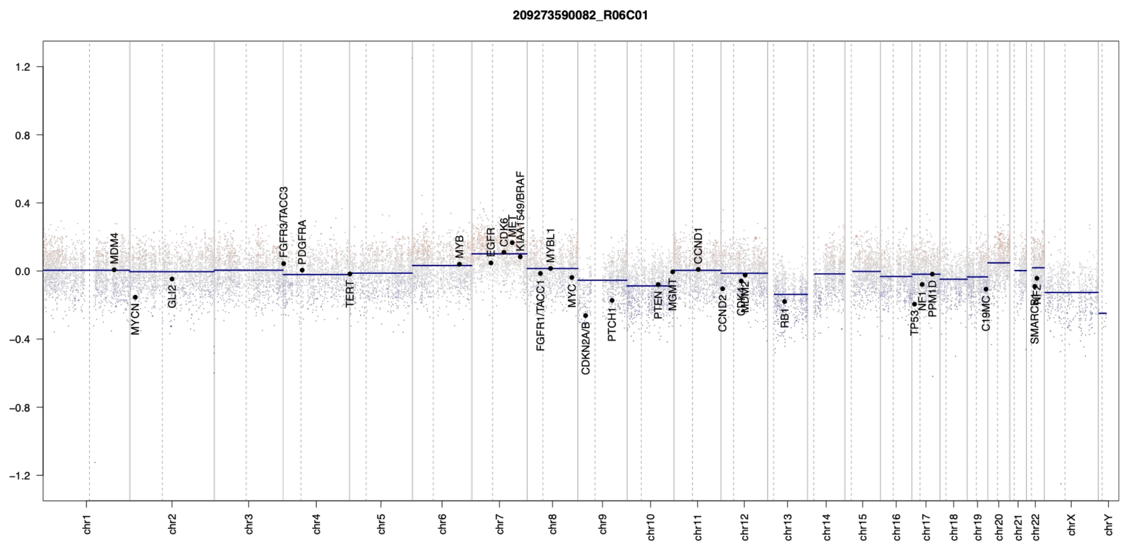

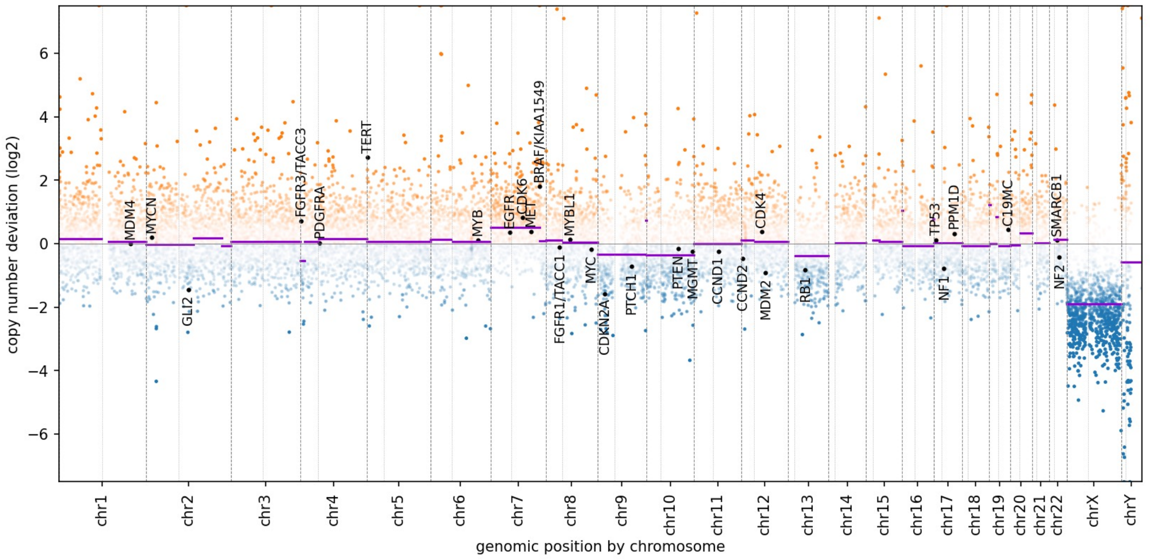

sample #19

- Gains: 7p, 7q, 20p
- Losses: 6q, 10p, 10q, 11q, 14q, 22q
- Focal CNVs: Del *CDKN2A/B*

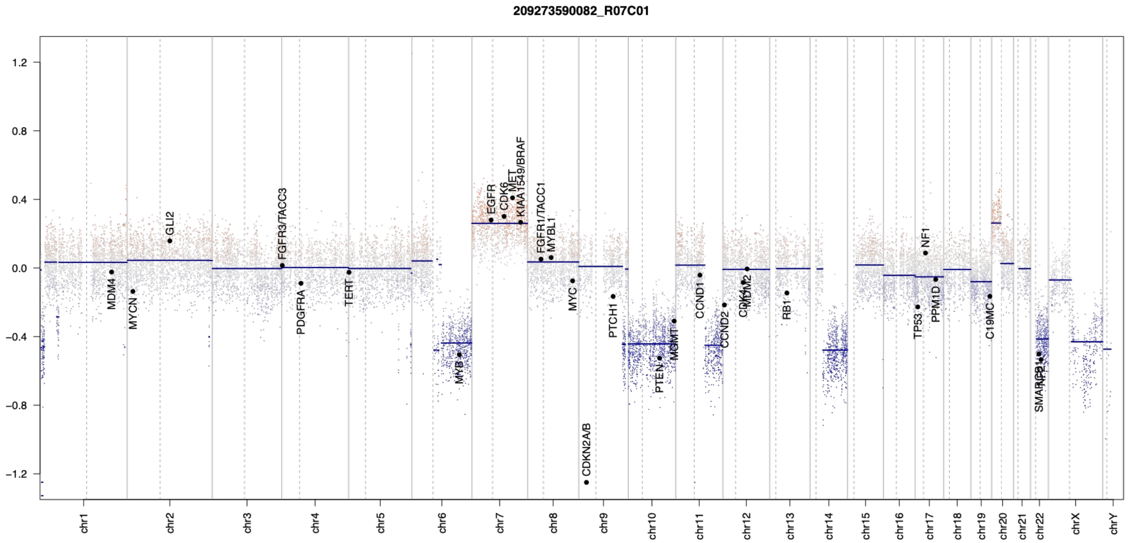

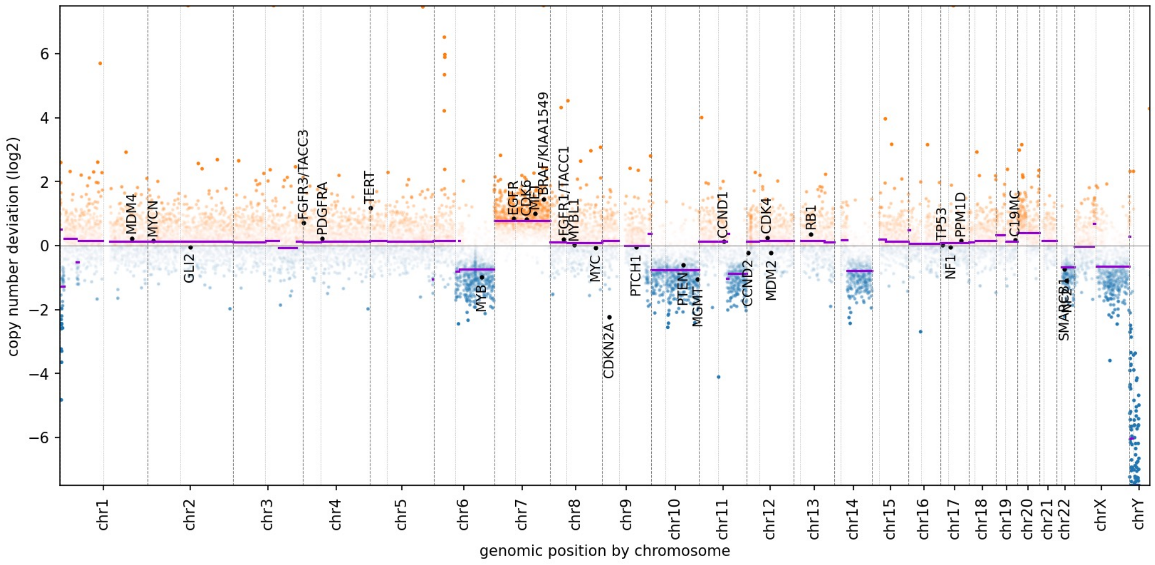

sample #20

- Gains: 7p, 7q
- Losses: 9p, 9q, 10p, 10q, 14q, 15q, 16q, 22q
- Focal CNVs: Del *CDKN2A/B*

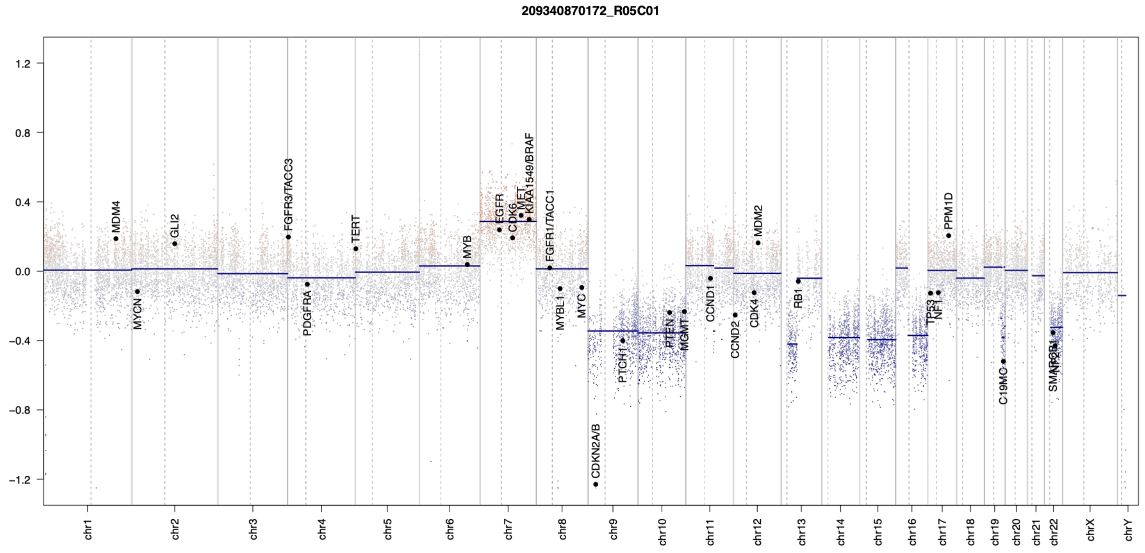

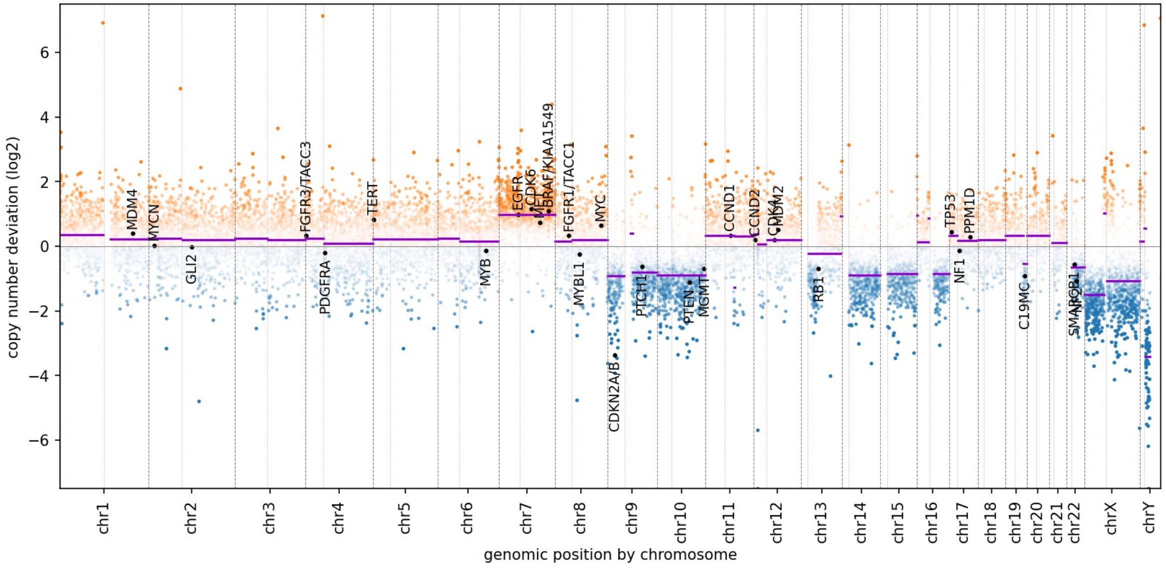

sample #21

- Gains: 6p, 6q, 7p, 7q, 9q
- Losses: 4p, 4q, 5q, 9p, 10p, 10q, 13q, 18p, 18q
- Focal CNVs: Del *CDKN2A/B*; Amp *EGFR, TERT*

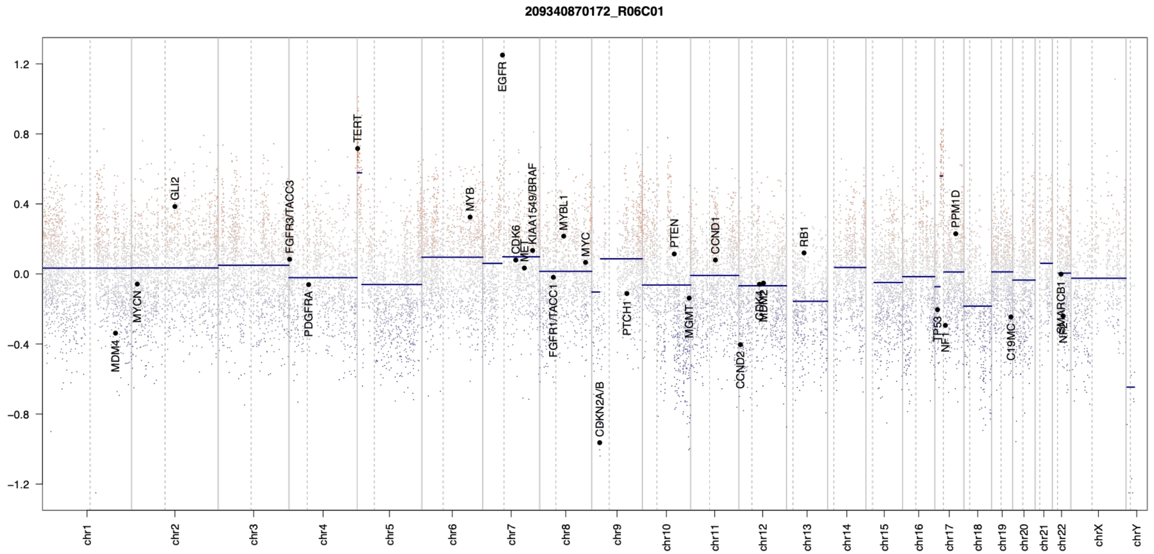

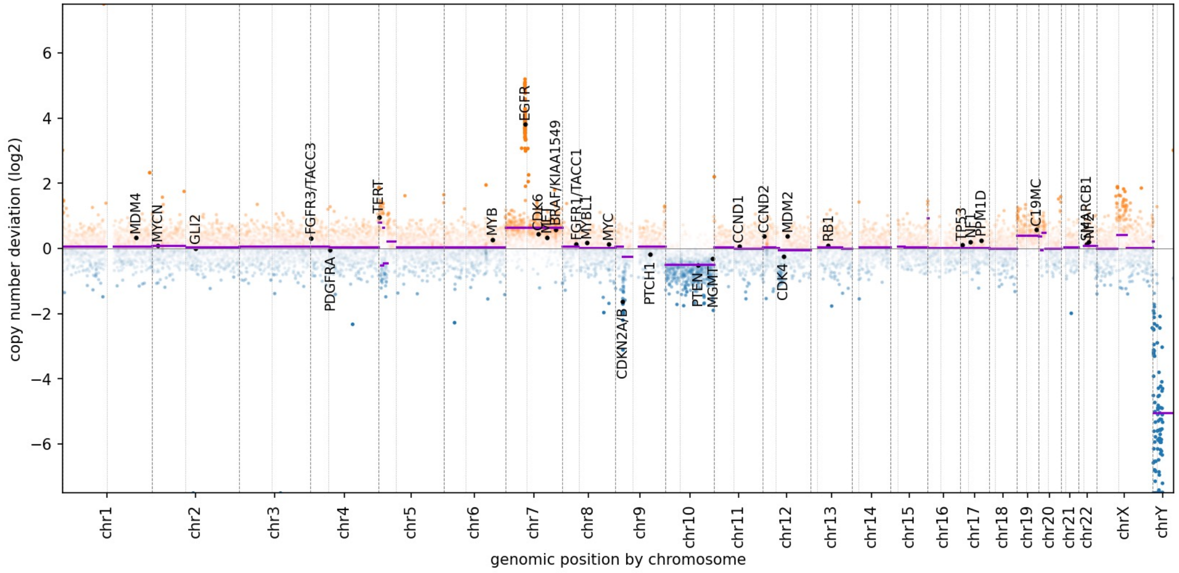

sample #22

- Gains: 2p, 2q, 4p, 4q, 5p, 7p, 7q, 20q
- Losses: 6q, 10p, 10q, 17p, 18p, 18q, 22q
- Focal CNVs: Del *CDKN2A/B*

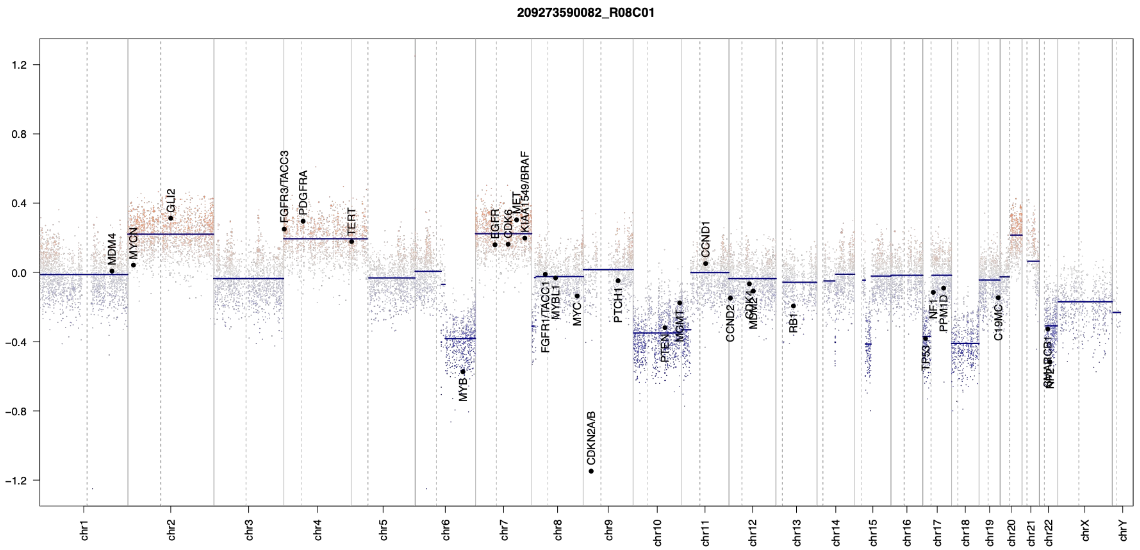

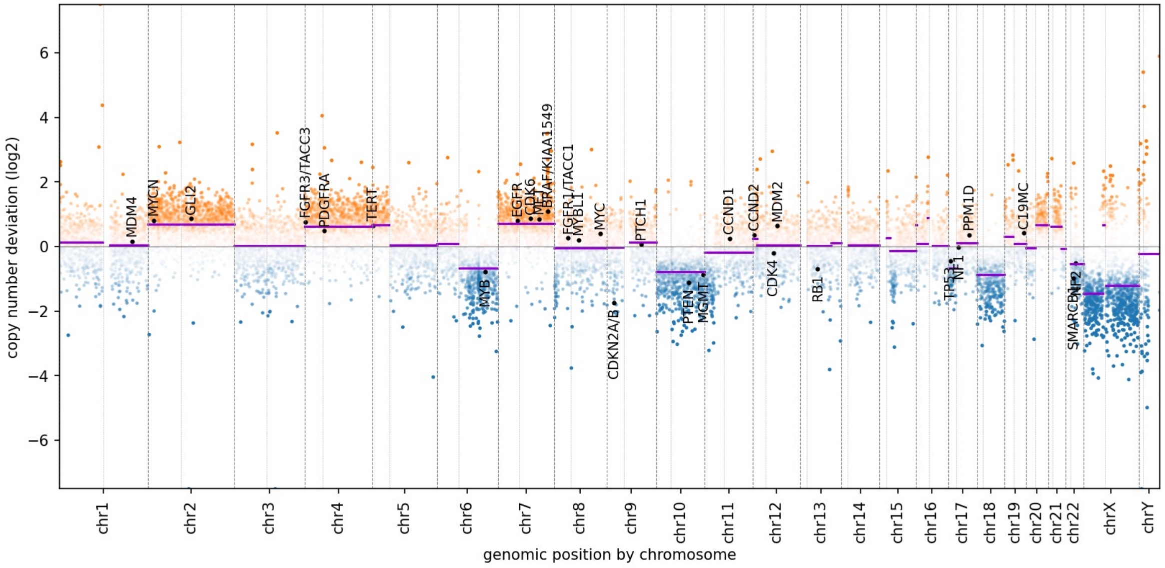

sample #23

- Gains: 7p, 7q
- Losses: 9p, 9q, 10q, 14q, 19q
- Focal CNVs: Del *CDKN2A/B*

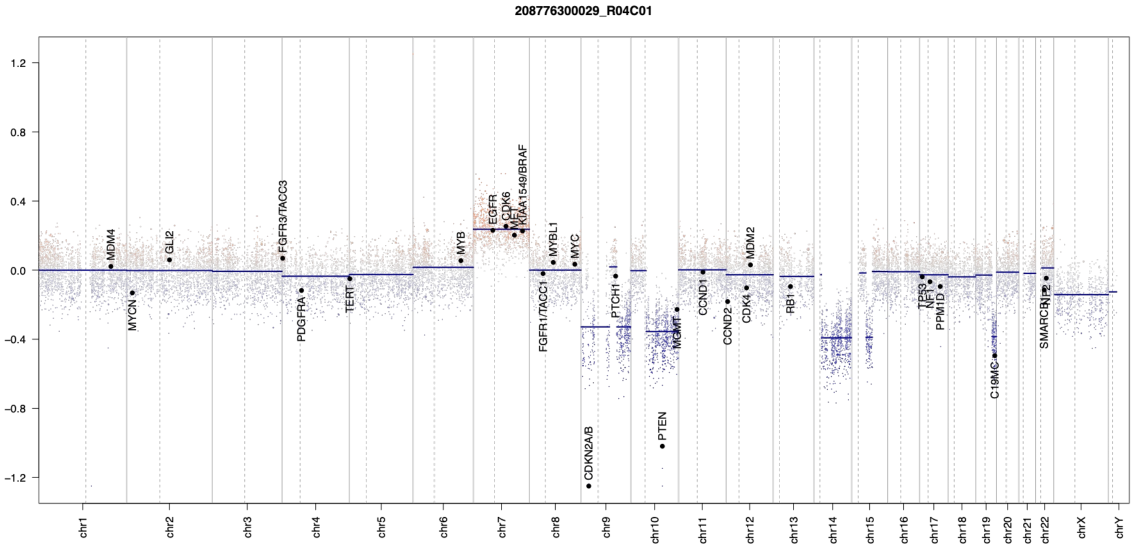

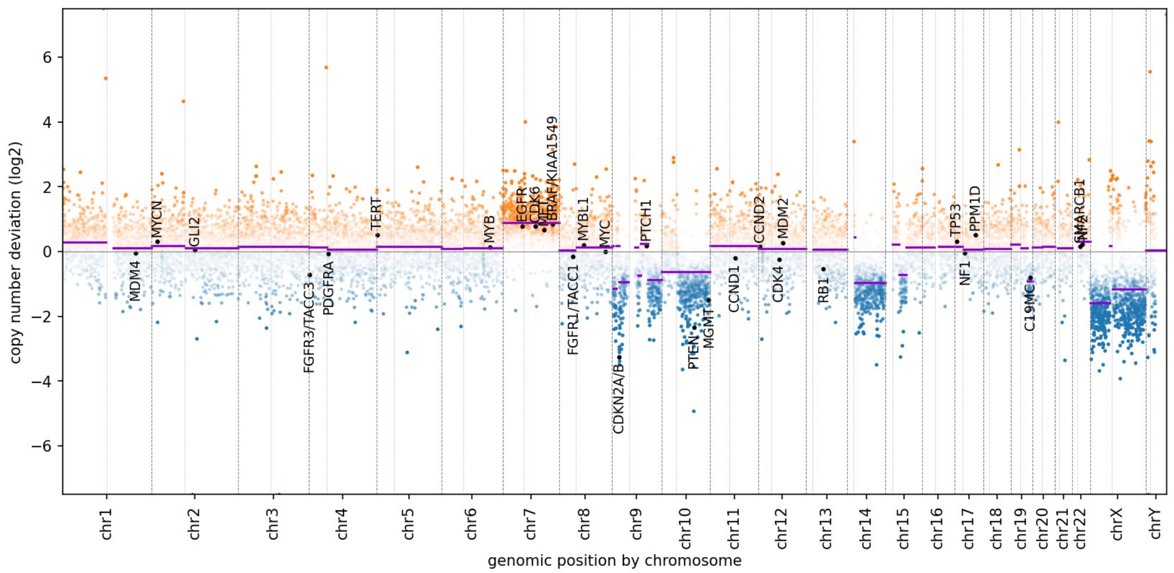

sample #24

- Gains: 7p, 7q, 17p, 17q, 20p, 20q
- Losses: 9p, 10p, 10q, 22q
- Focal CNVs: Del *CDKN2A/B*

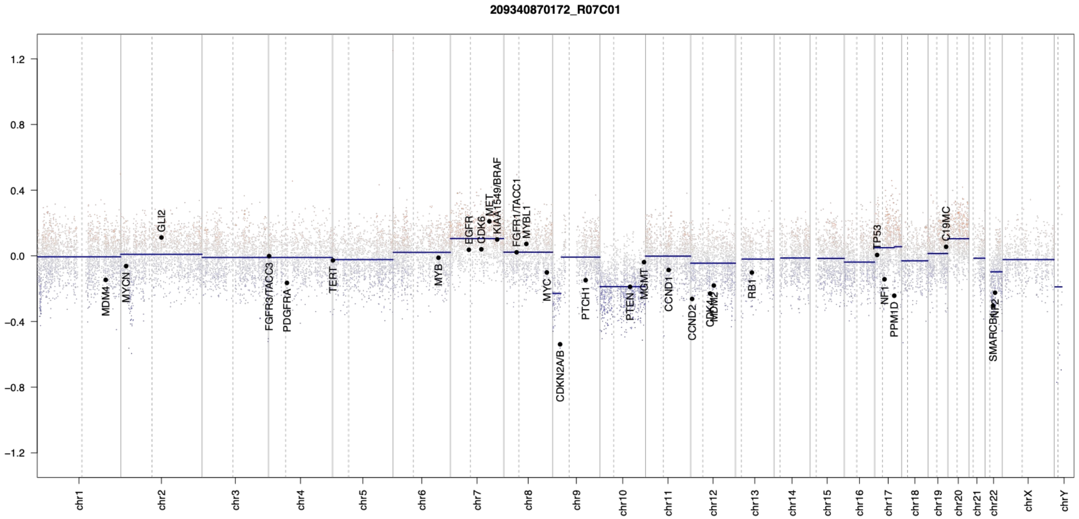

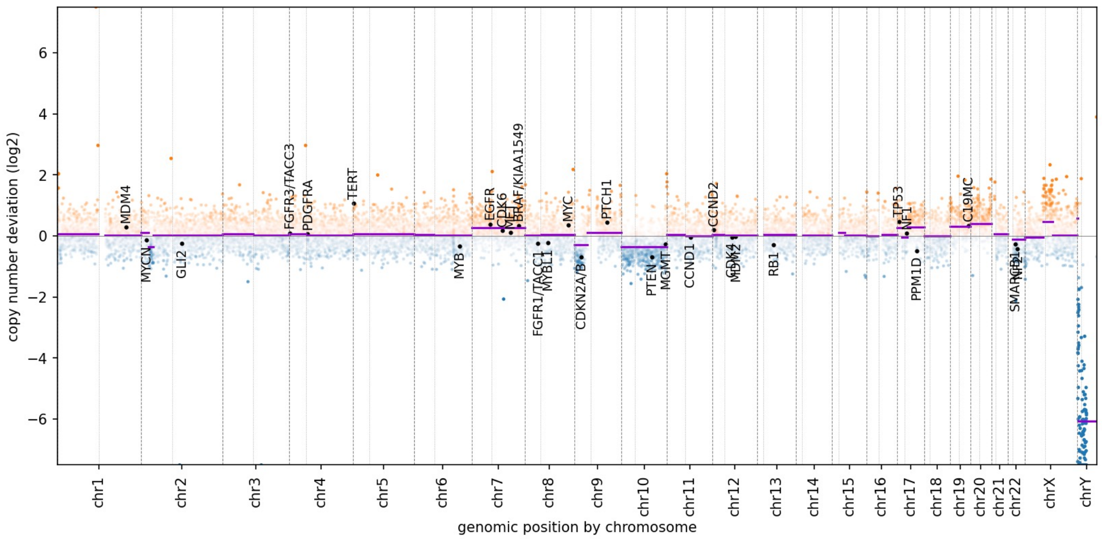

sample #25

- Gains: 7p, 7q
- Losses: 9p, 10p, 10q, 14q
- Focal CNVs: Del *CDKN2A/B*; Amp *EGFR*

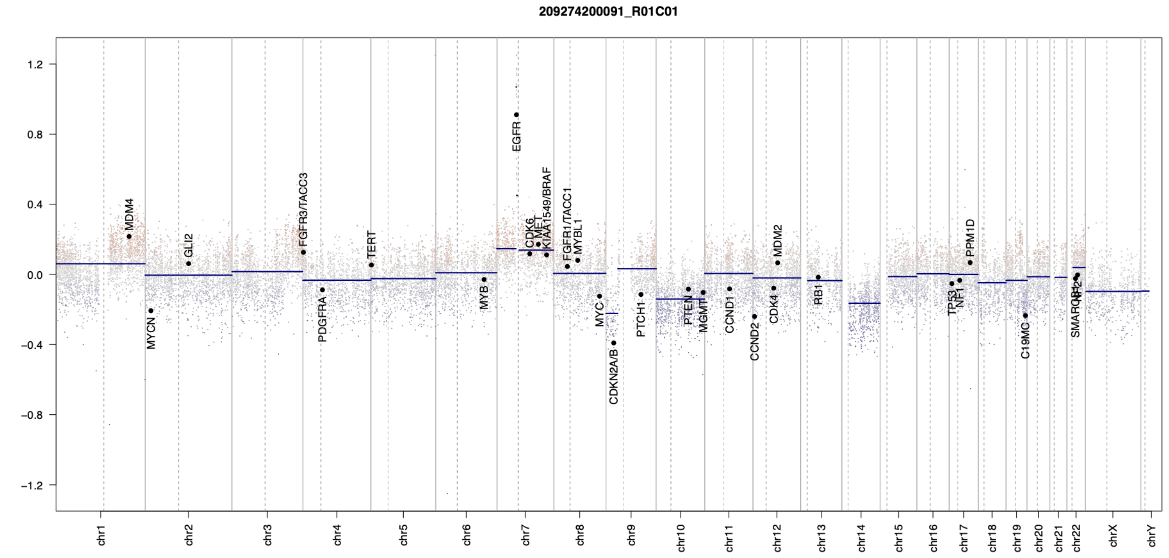

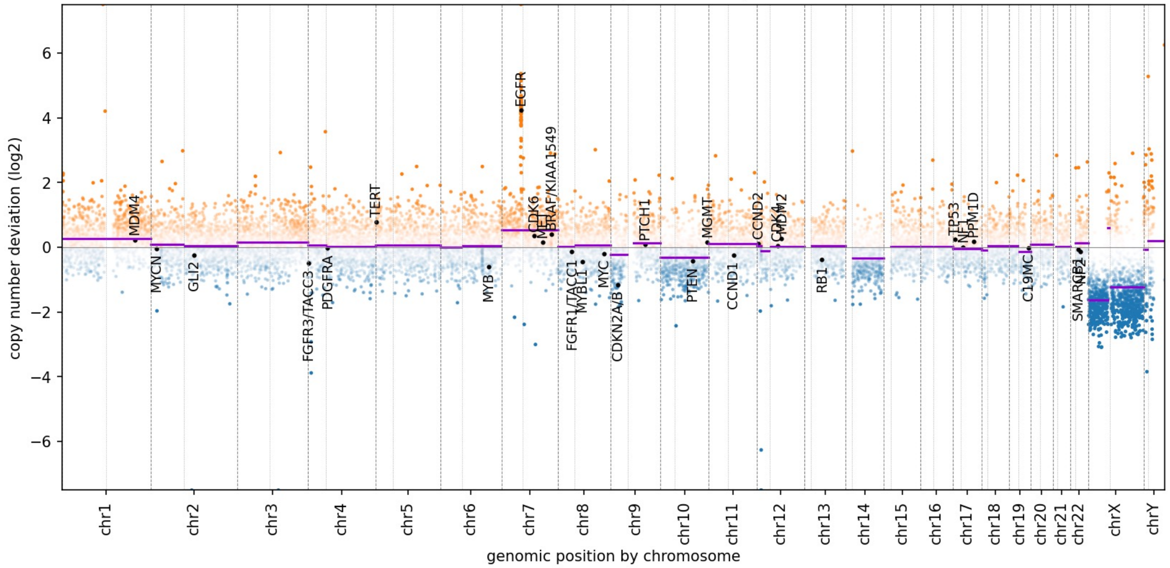

sample #26

- Gains: 21q
- Losses: 1p, 4p, 4q, 8p, 9p, 14q, 18p, 18q, 19q
- Focal CNVs: Del *CDKN2A/B*

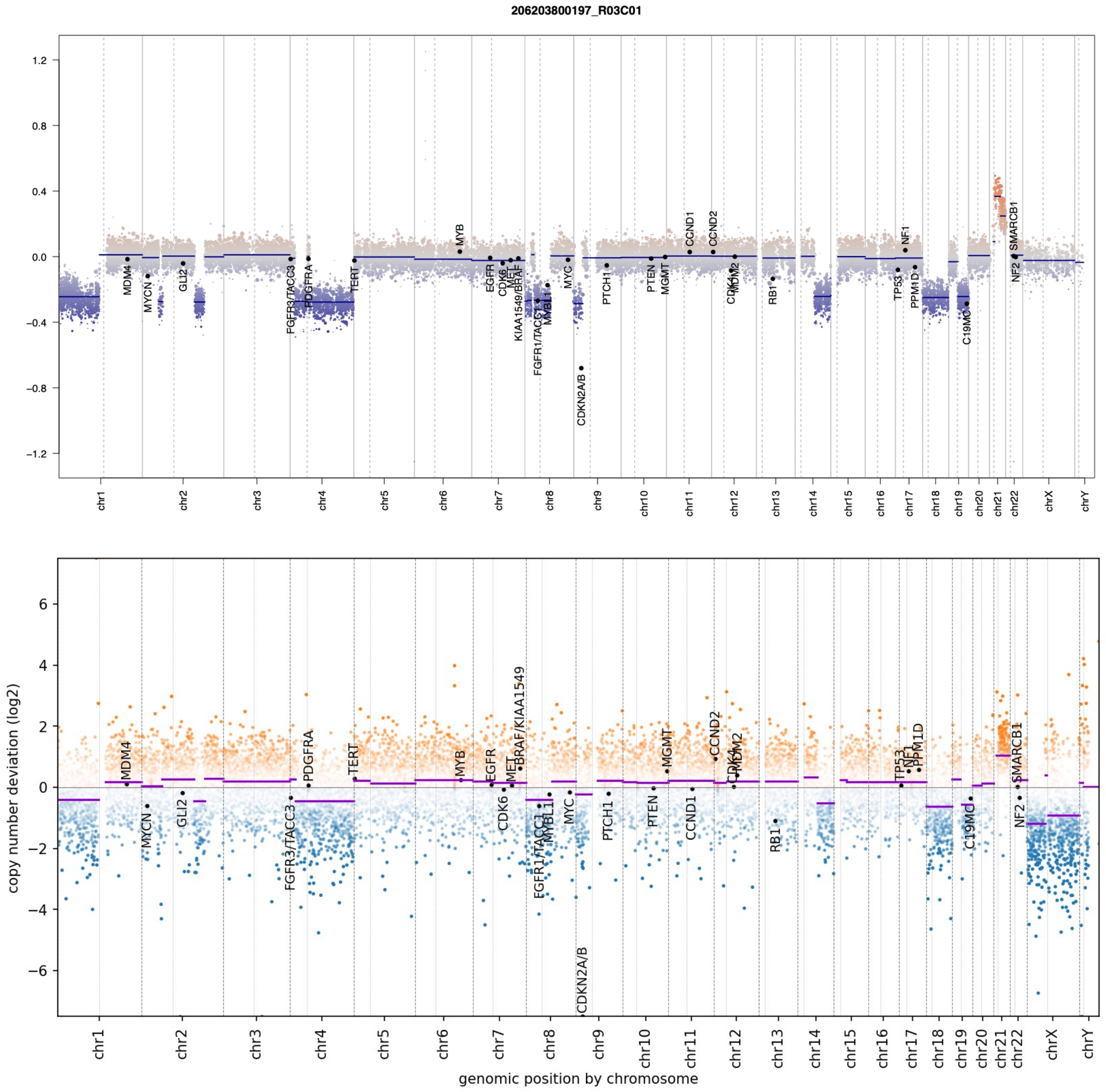

sample #27

- Gains: 11q
- Losses: 1p, 2q, 15q, 19q
- Focal CNVs: -

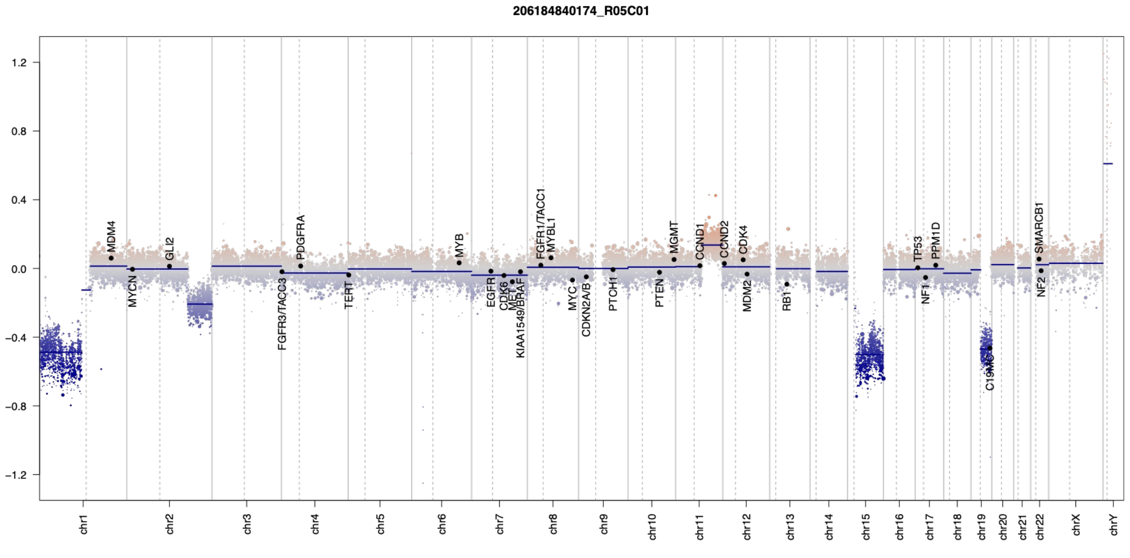

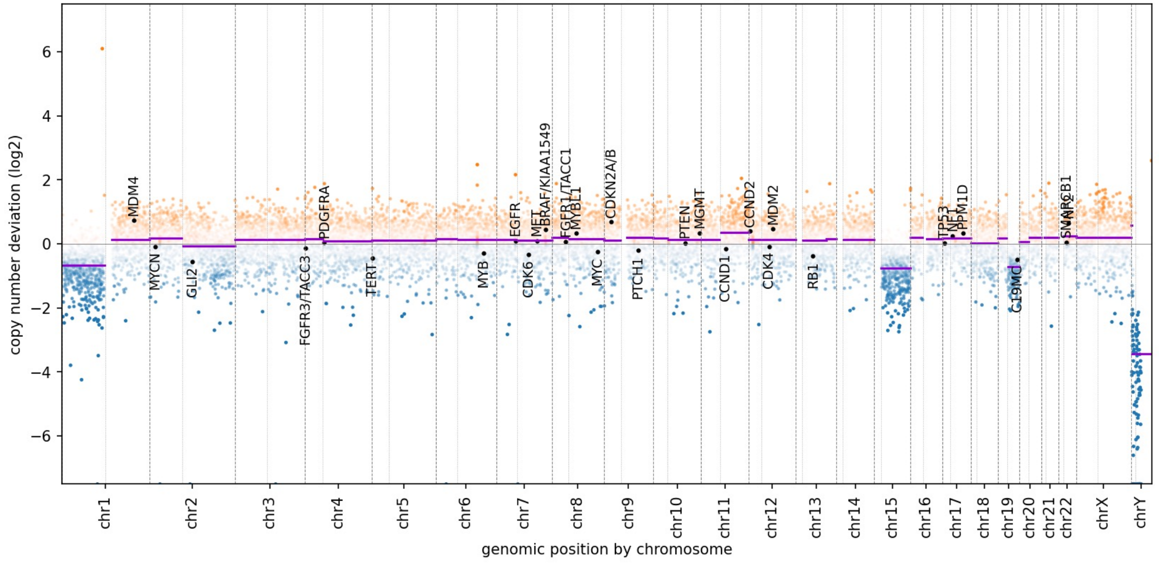

sample #28

- Gains: -
- Losses: 1p, 19q
- Focal CNVs: -

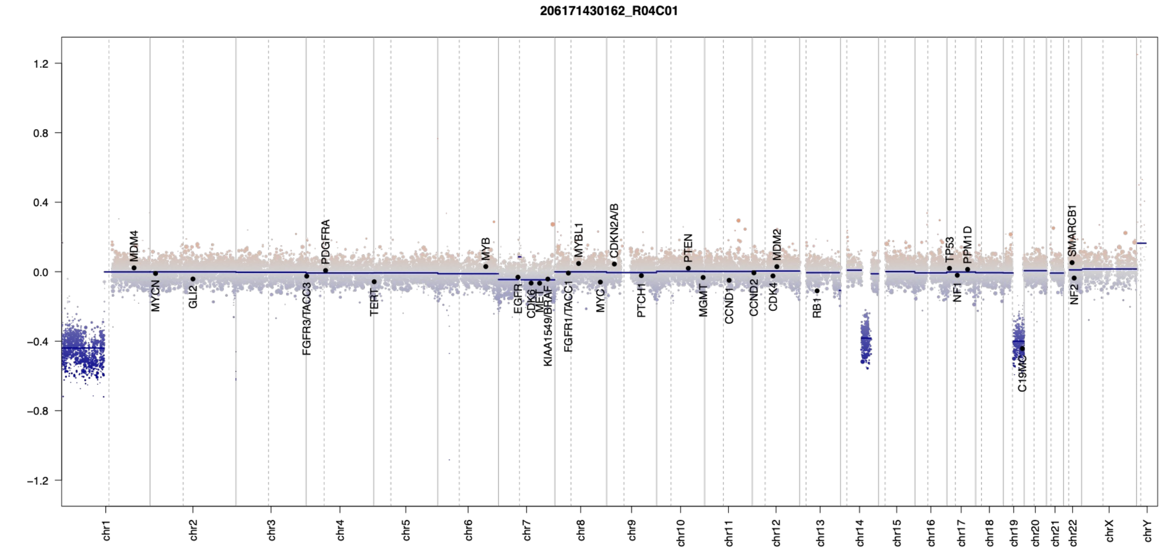

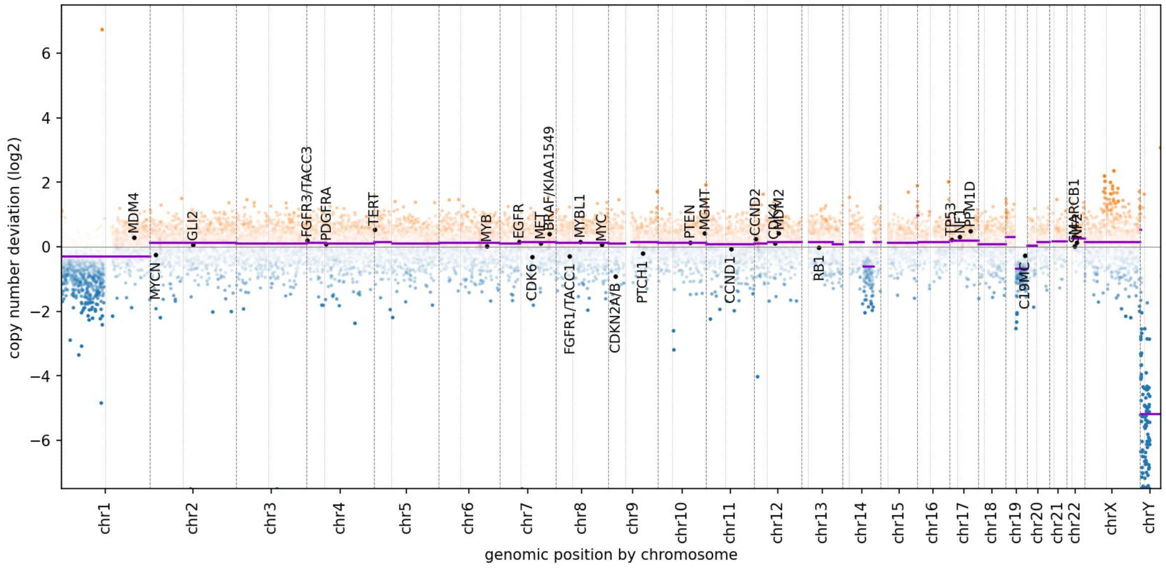

sample #29

- Gains: -
- Losses: 1p, 4p, 4q, 9p, 9q, 14q, 19q
- Focal CNVs: -

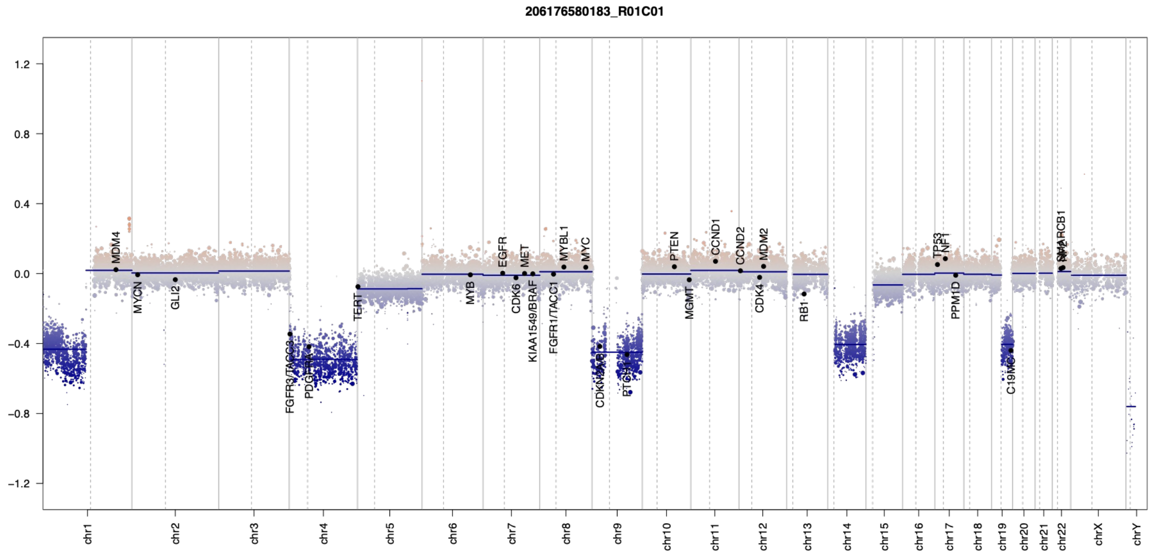

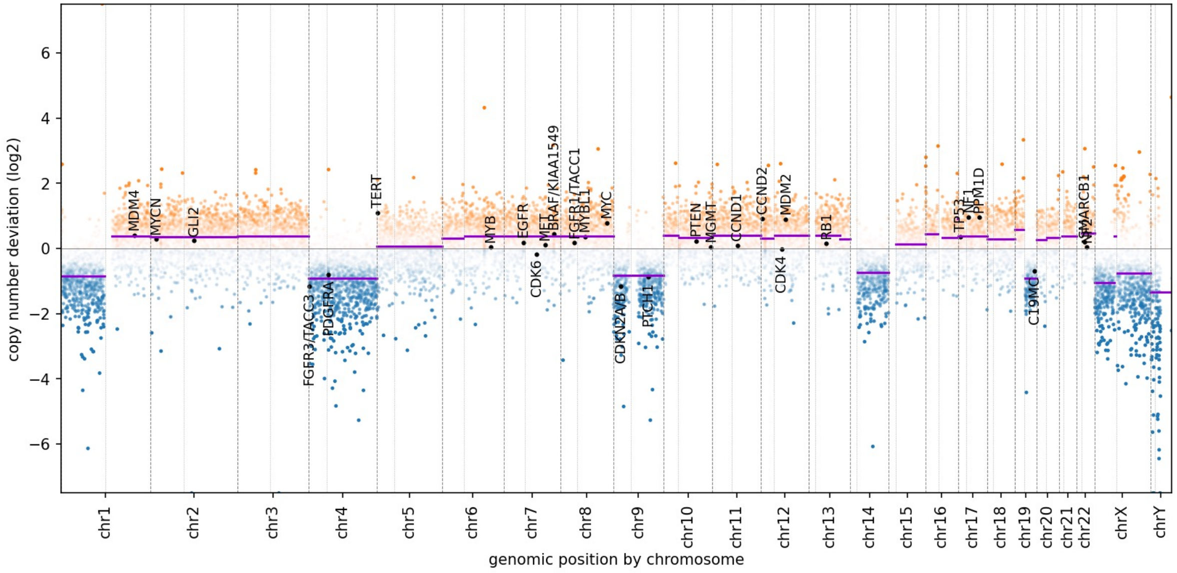

sample #30

- Gains: -
- Losses: 1p, 19q
- Focal CNVs: -

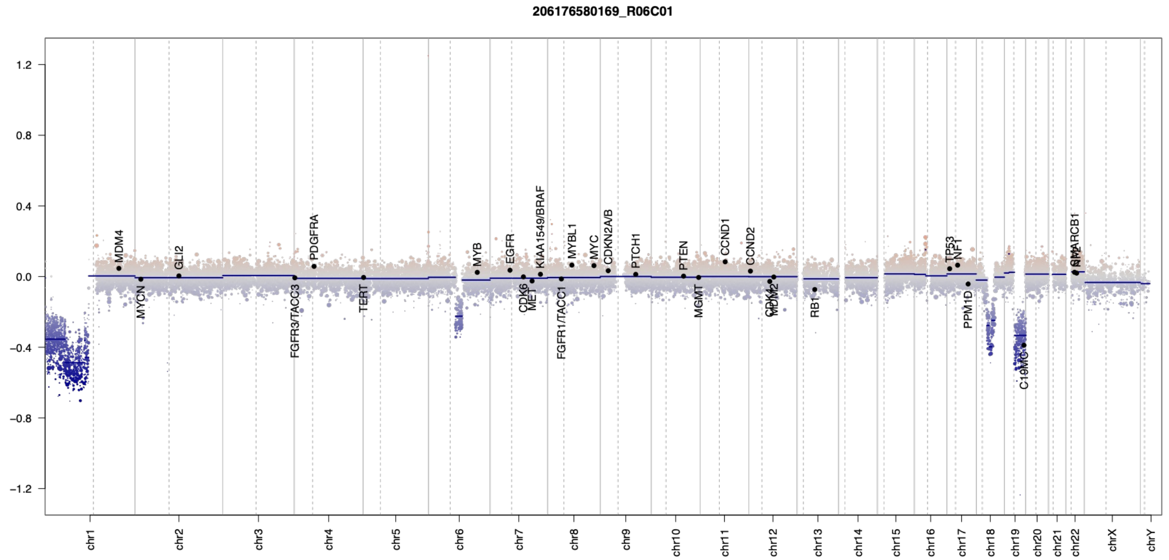

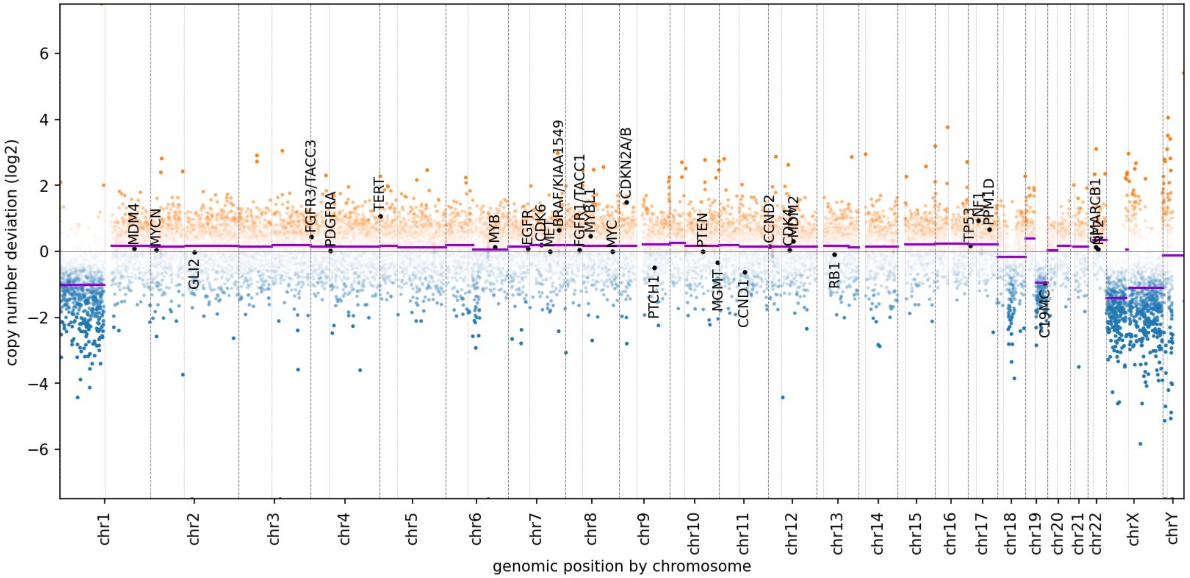

sample #31

- Gains: -
- Losses: 1p, 4q, 14q, 15q, 18p, 18q, 19q
- Focal CNVs: Amp *PDGFRA*

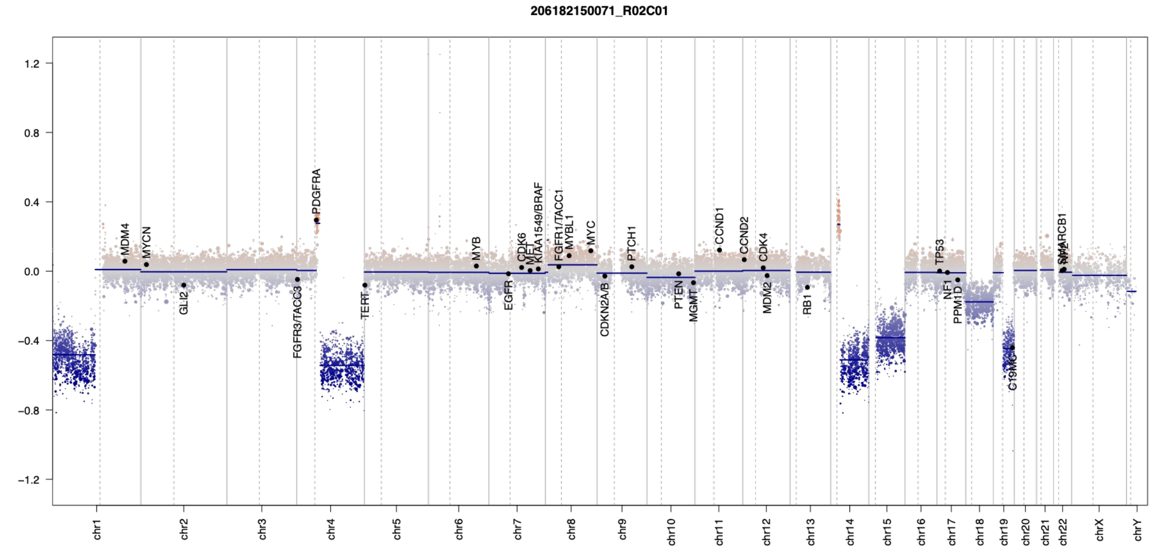

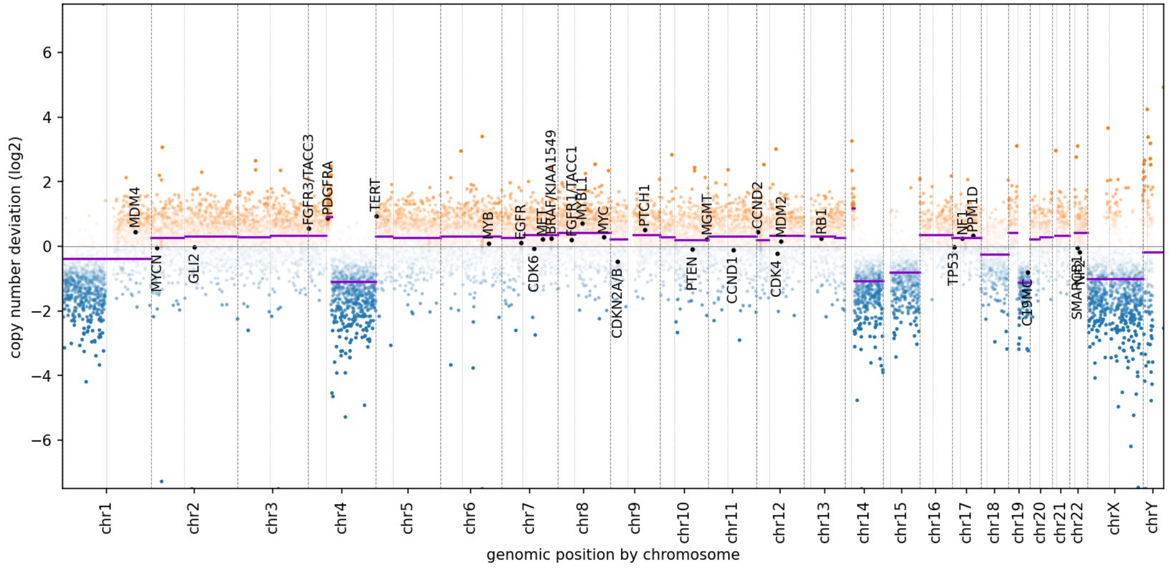

sample #32

- Gains: -
- Losses: 1p, 19q
- Focal CNVs: -

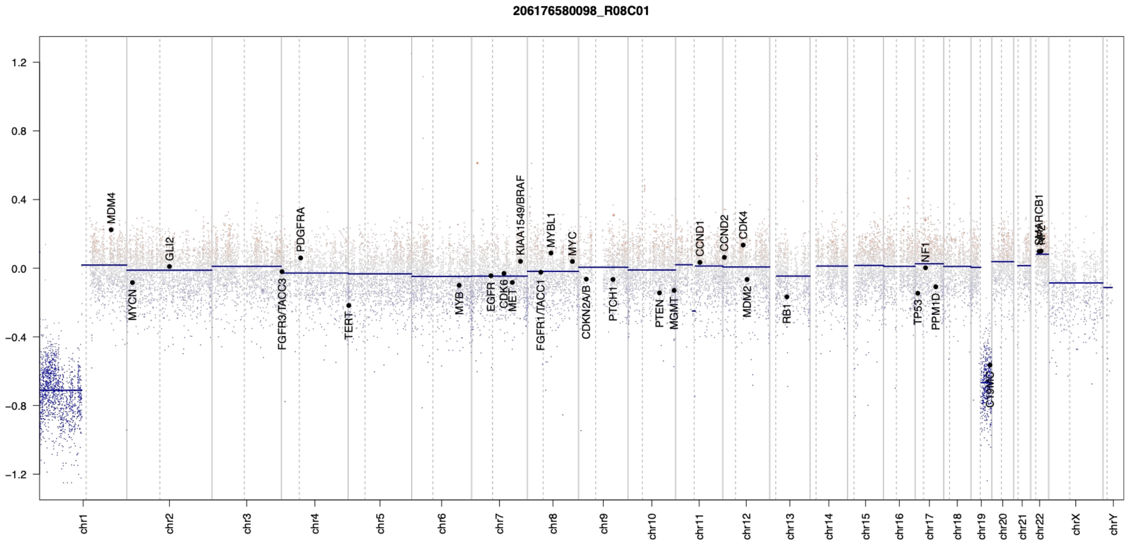

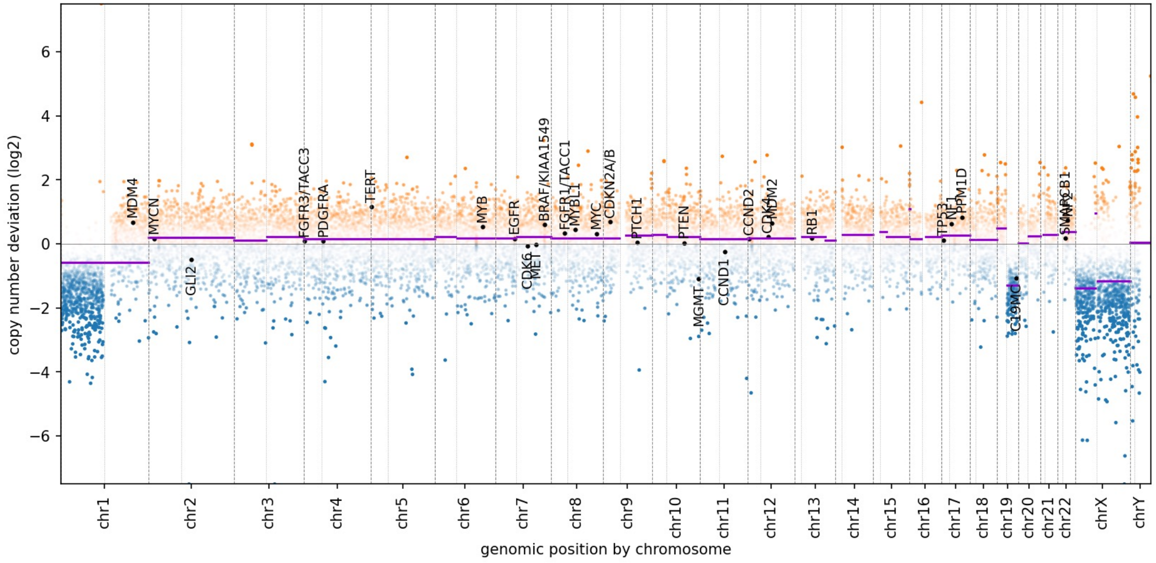

sample #33

• Gains: -

• Losses: 1p, 2p, 2q, 4p, 4q, 5p, 5q, 6p, 6q, 9p, 9q, 15q, 18p, 18q, 19q, 20p, 20q

• Focal CNVs: -

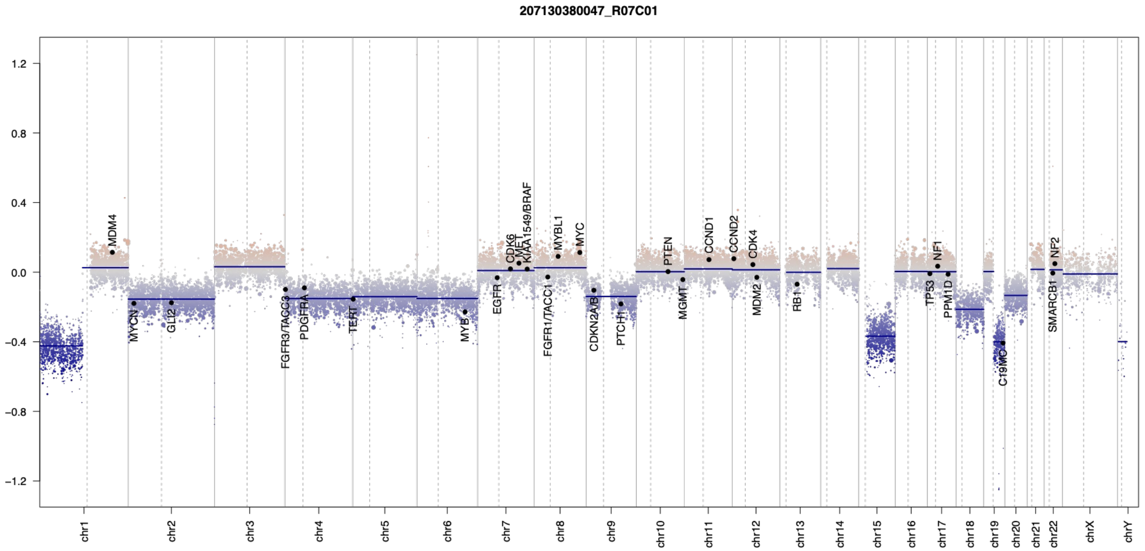

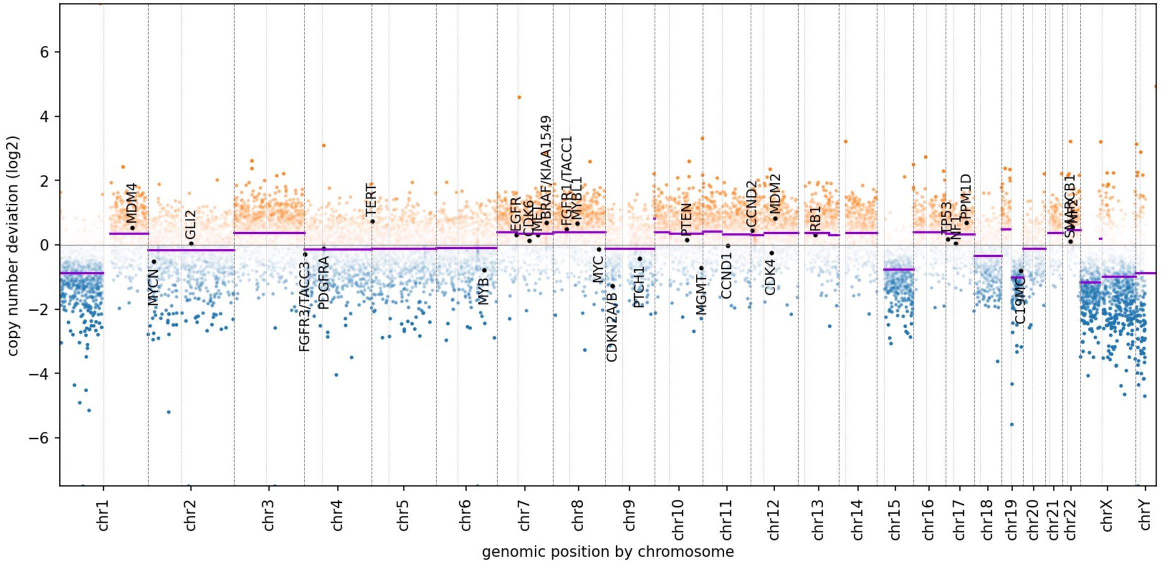

sample #34

- Gains: -
- Losses: 1p, 4p, 4q, 5p, 5q, 6p, 6q, 9p, 9q, 12p, 12q, 15q, 18p, 18q, 19q
- Focal CNVs: -

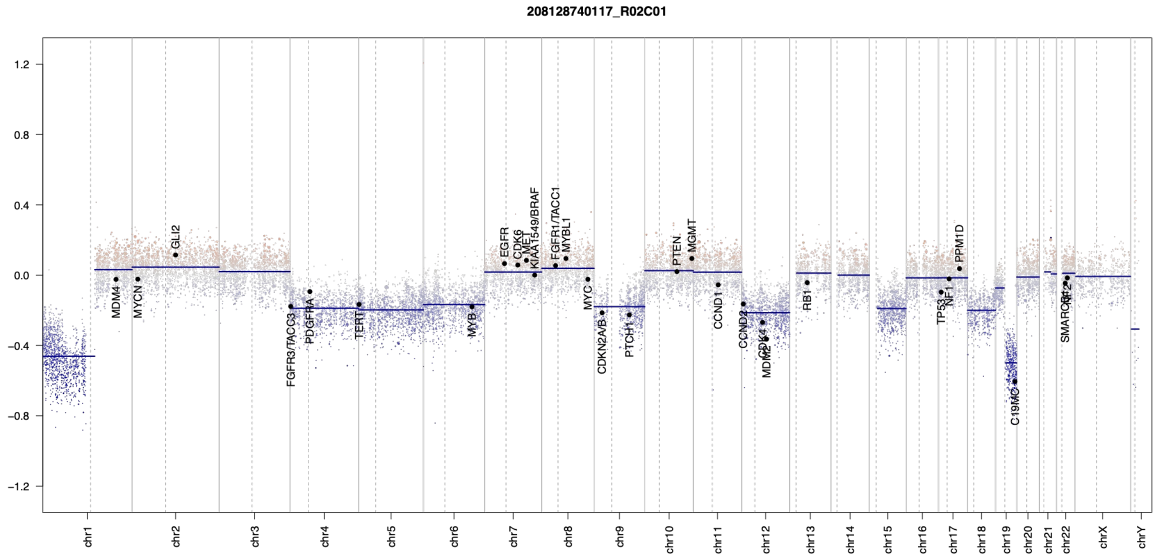

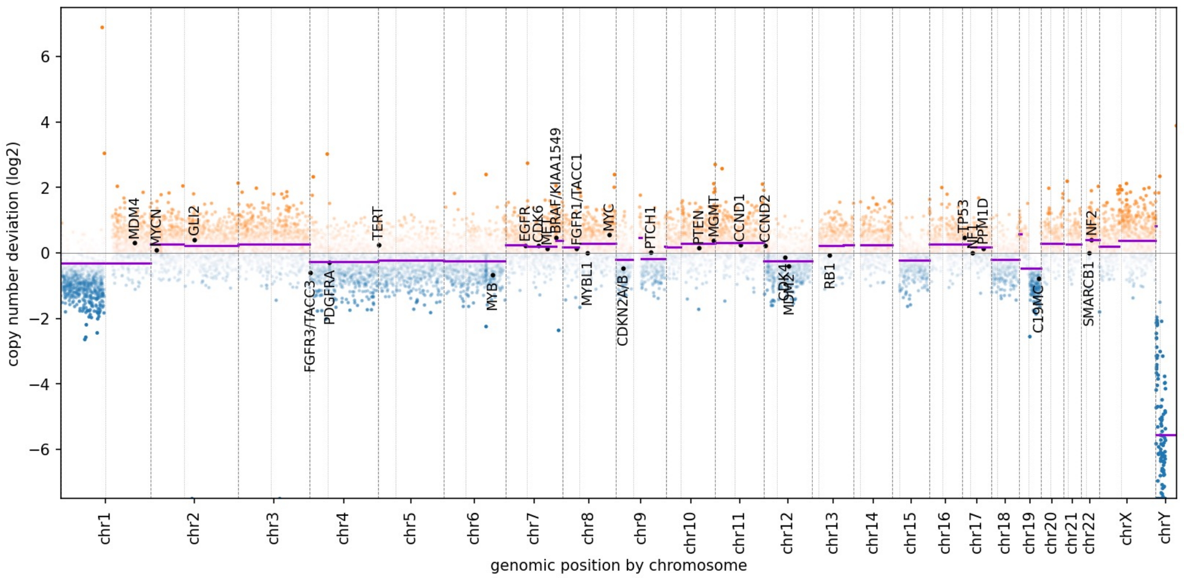

sample #35

- Gains: -
- Losses: 1p, 19q
- Focal CNVs: -

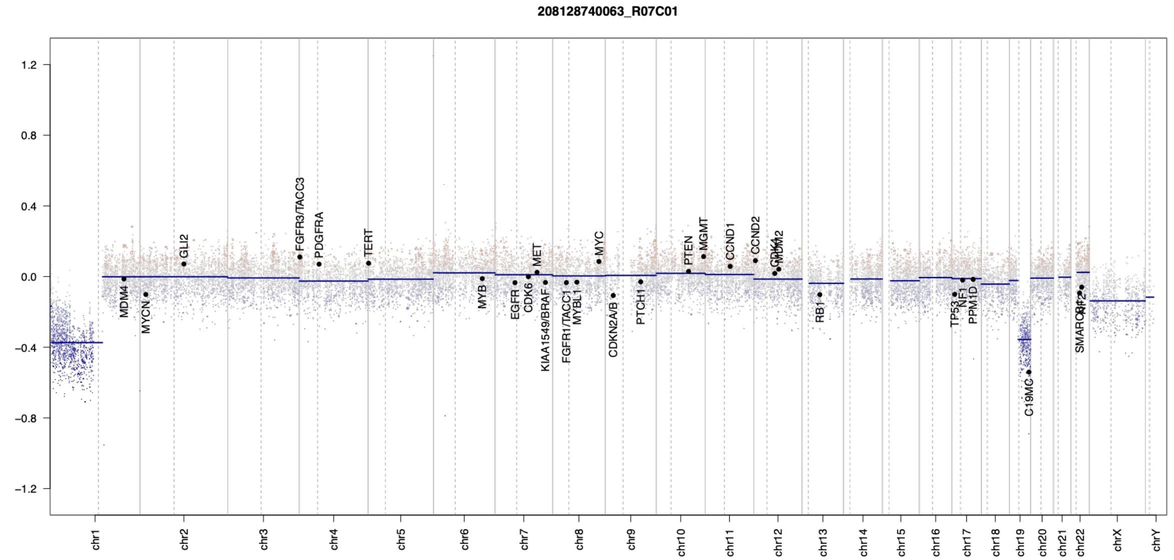

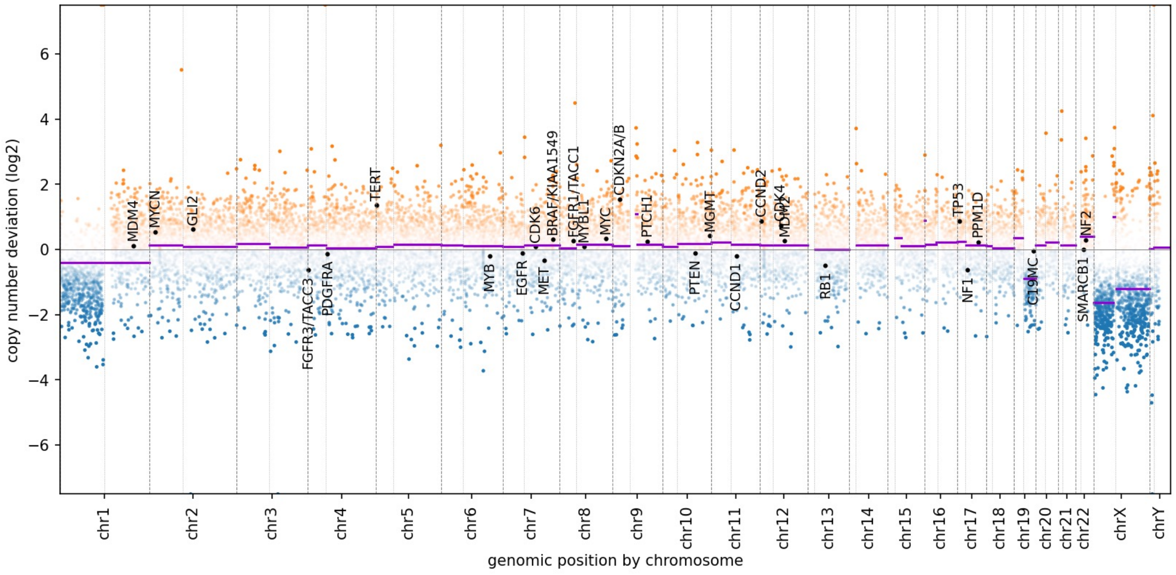

sample #36

- Gains: -
- Losses: 1p, 19q
- Focal CNVs: -

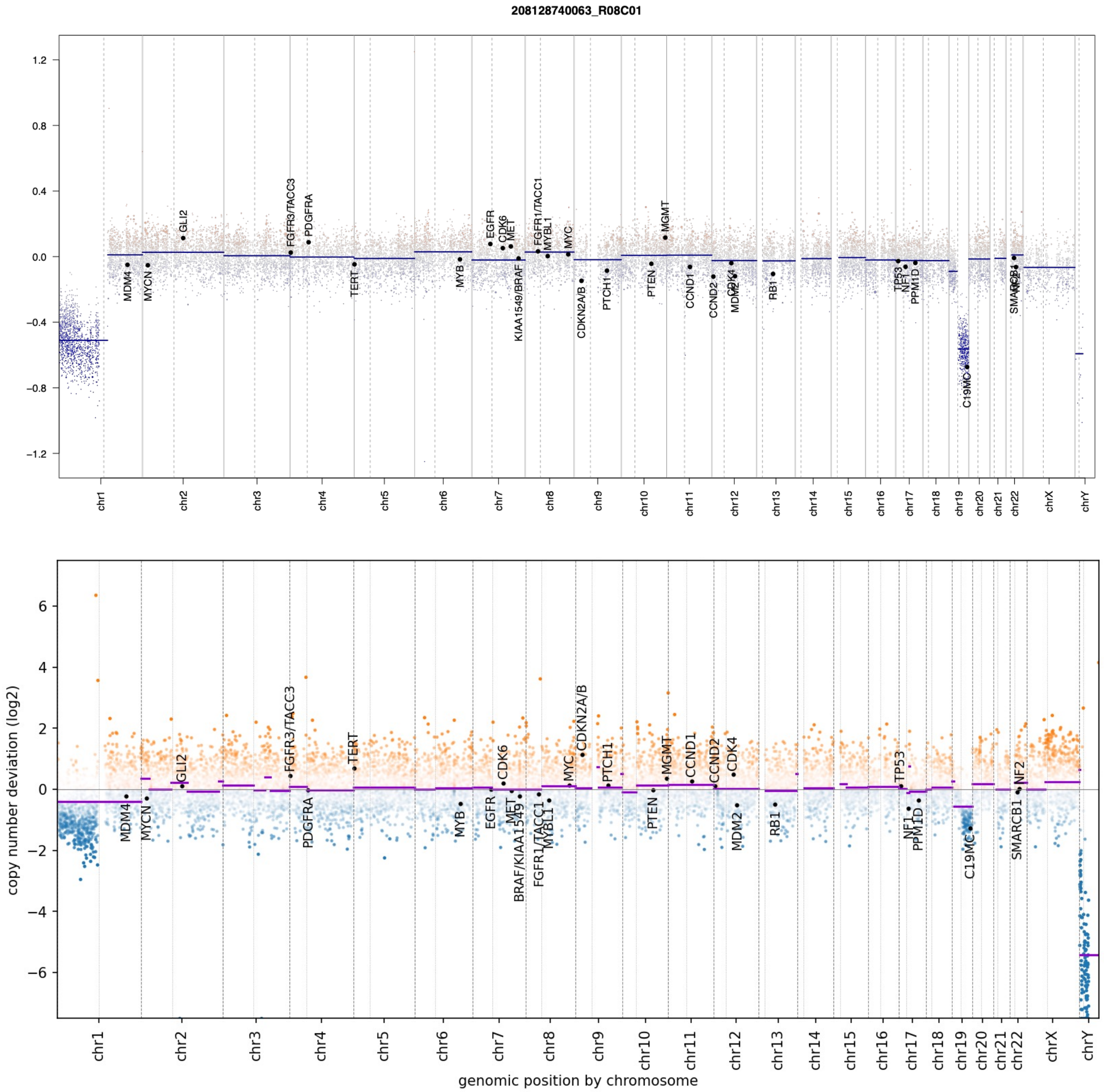

sample #37

- Gains: -
- Losses: 1p, 19q
- Focal CNVs: -

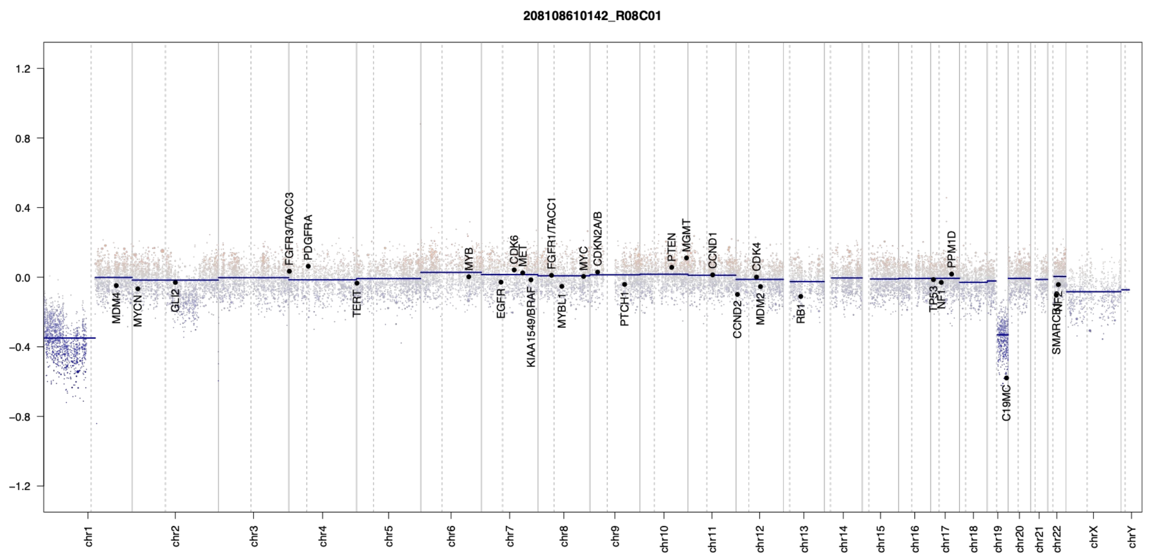

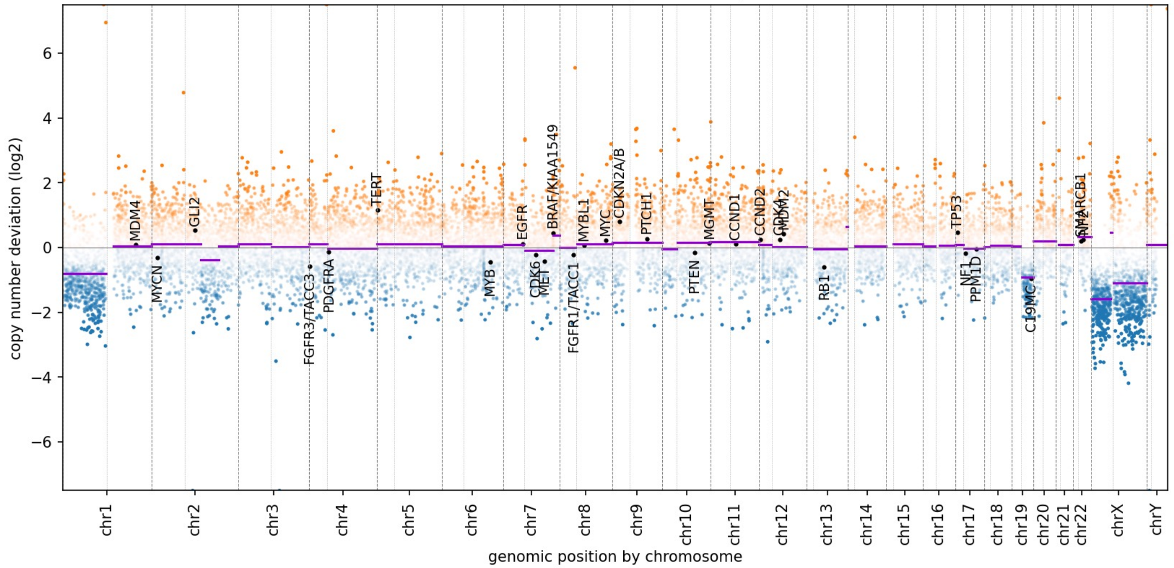

sample #38

- Gains: -
- Losses: 1p, 19q
- Focal CNVs: -

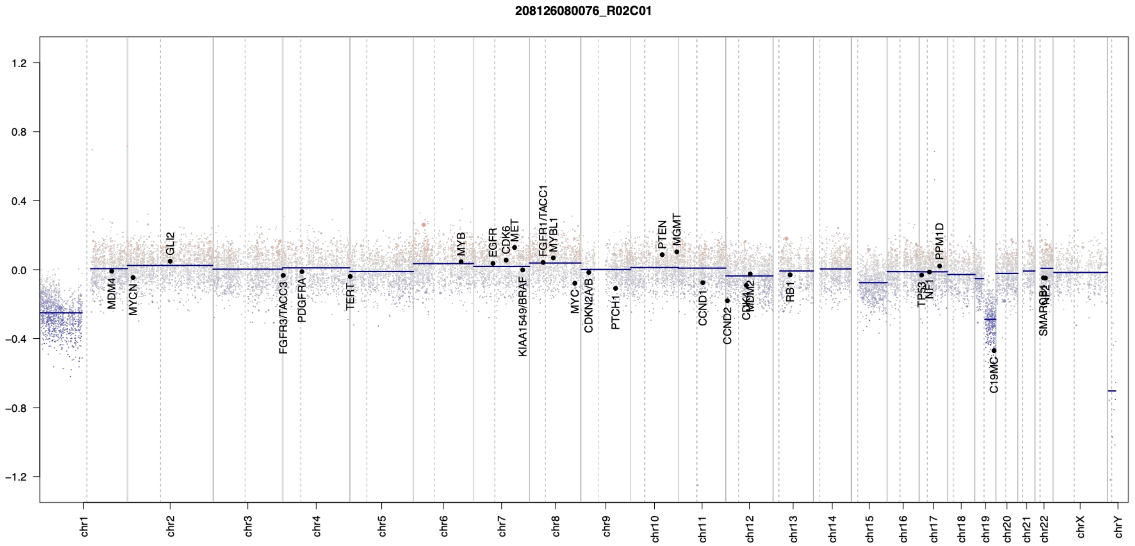

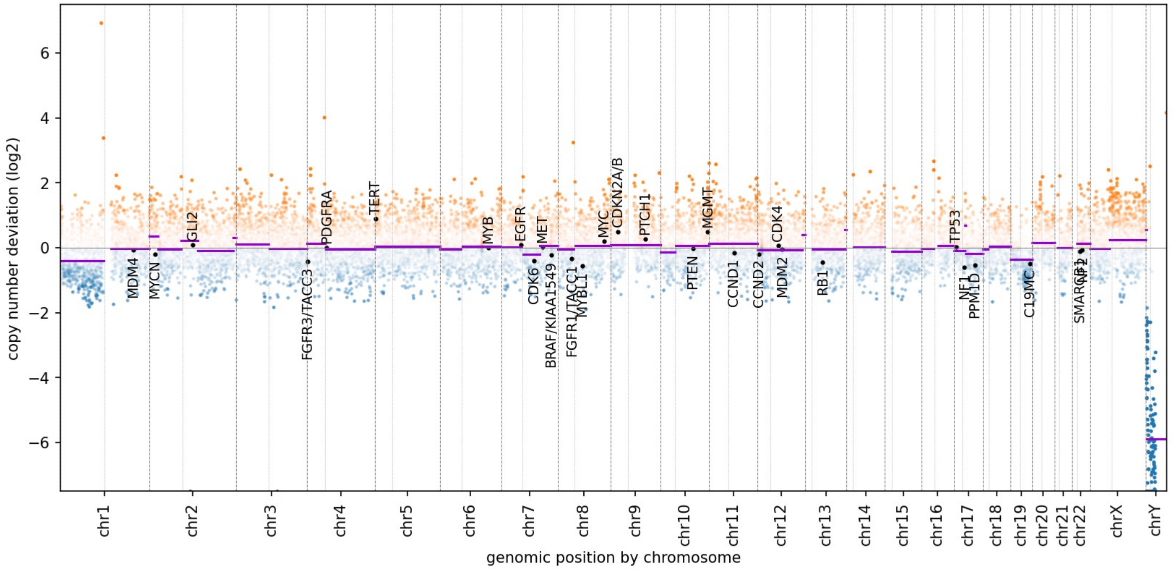

sample #39

- Gains: -
- Losses: 1p, 19q
- Focal CNVs: -

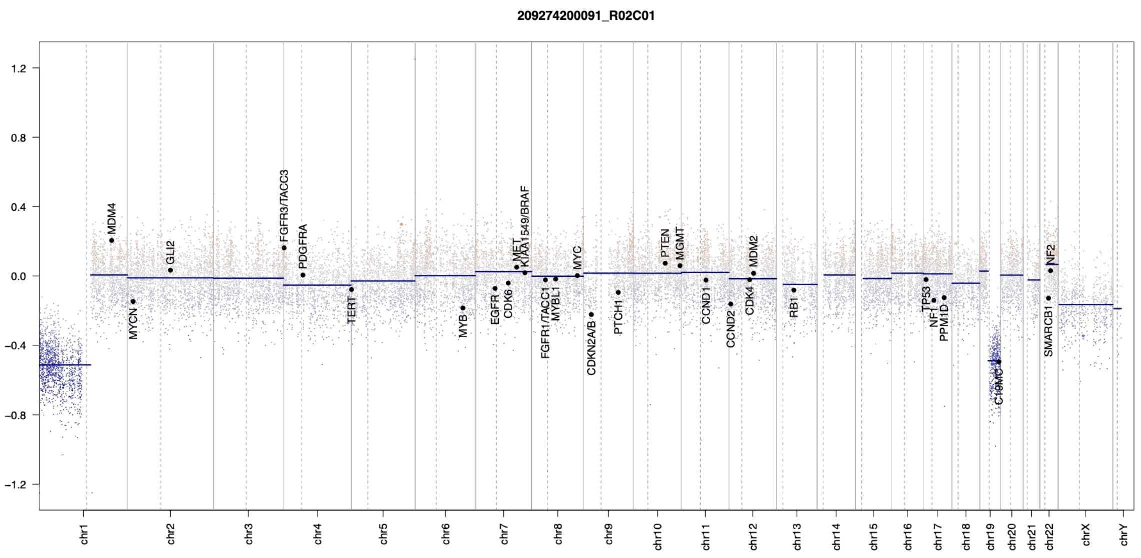

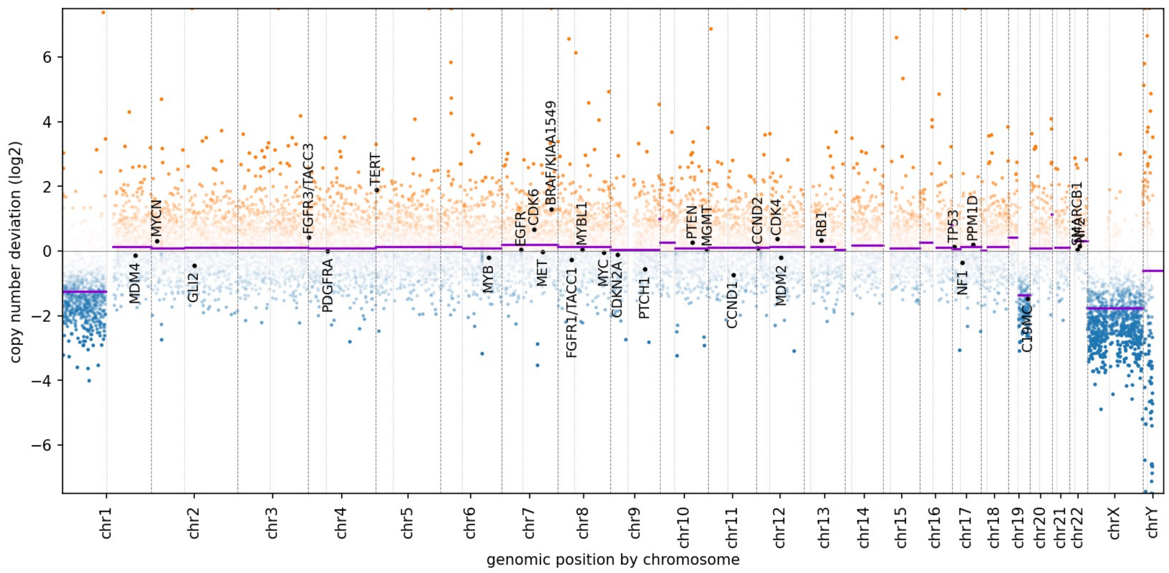

sample #40

- Gains: -
- Losses: 1p, 19q
- Focal CNVs: -

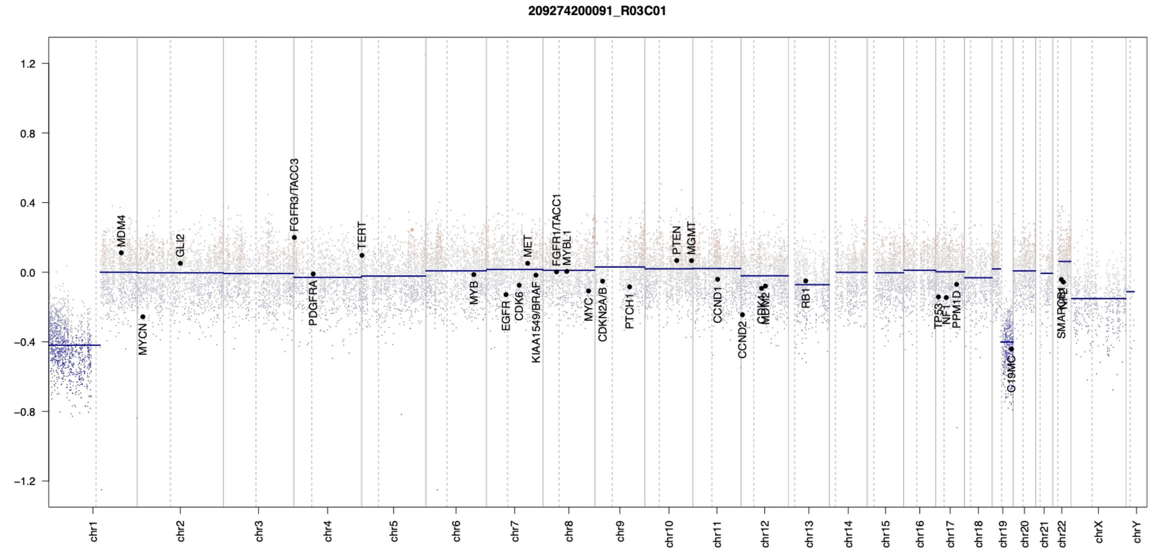

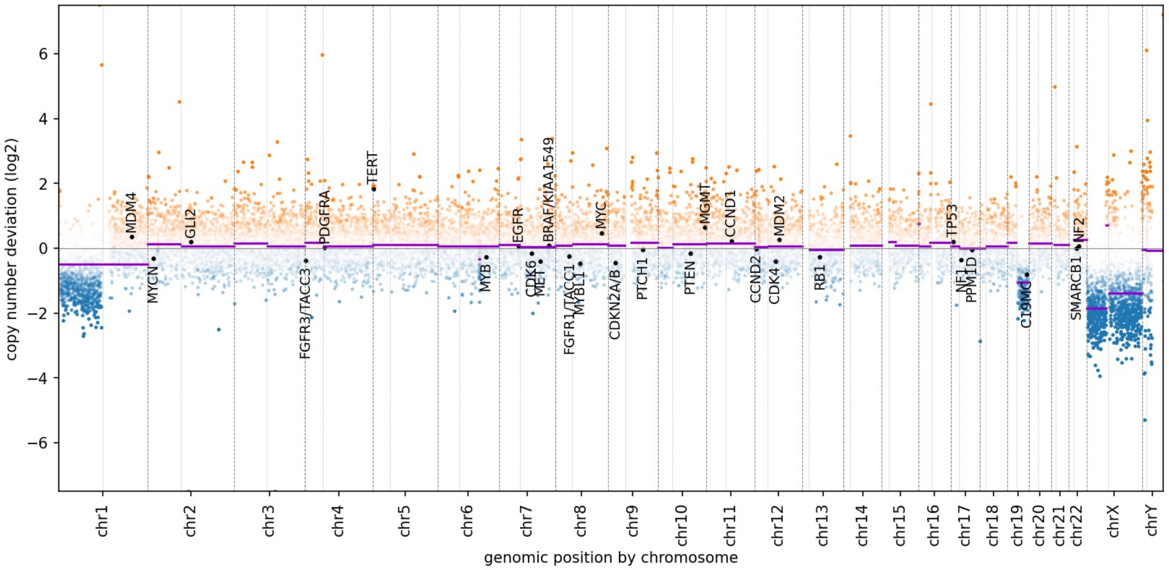

sample #41

- Gains: 2p, 2q, 3q
- Losses: -
- Focal CNVs: -

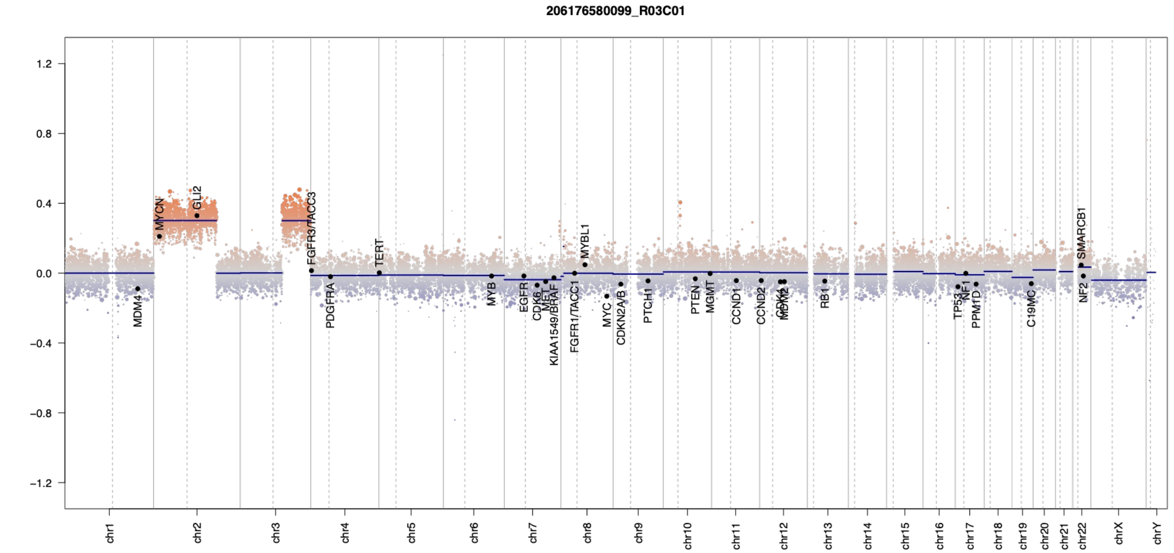

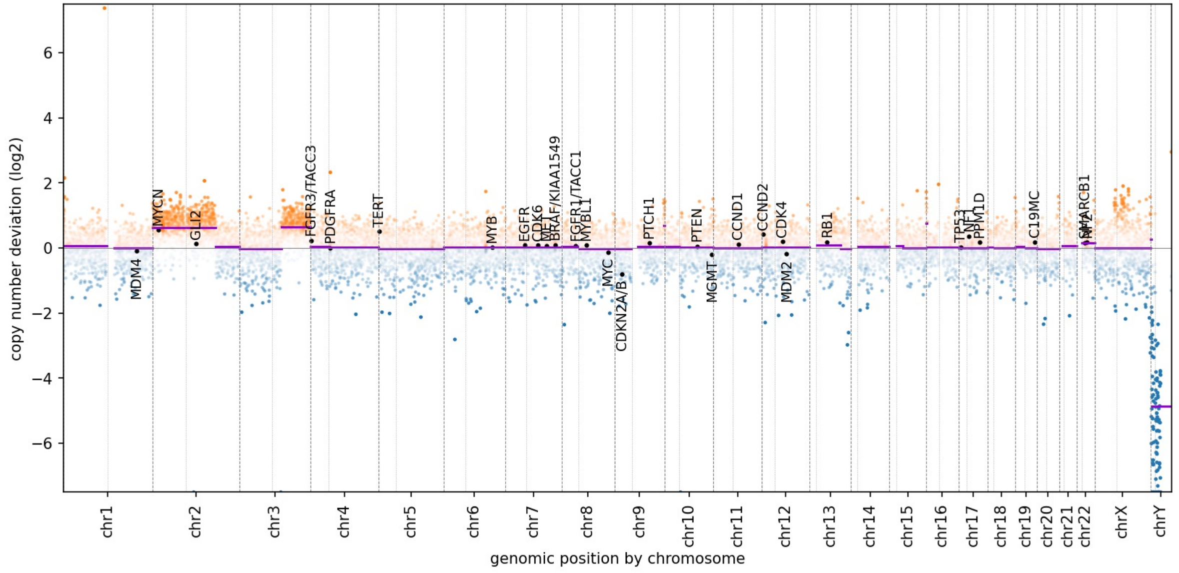

sample #42

- Gains: -
- Losses: -
- Focal CNVs: -

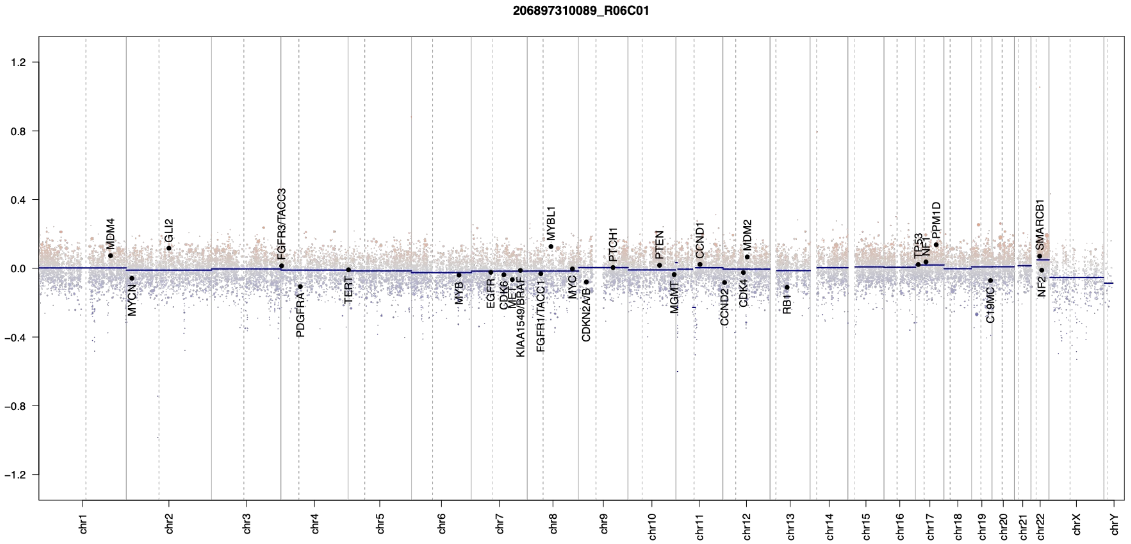

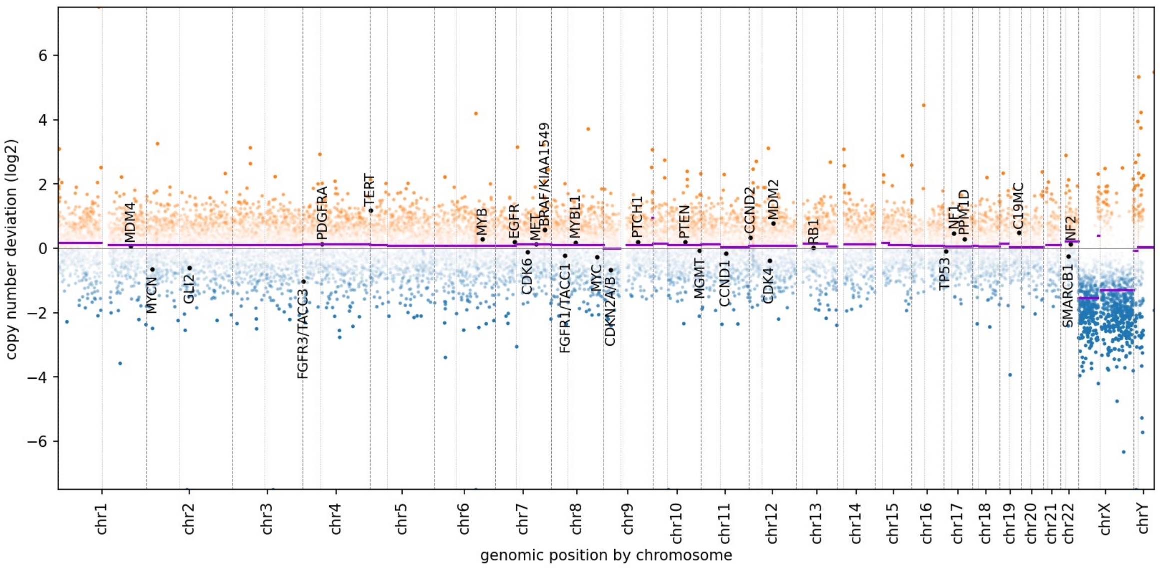

sample #43

- Gains: 1q, 6p, 6q, 8q, 12p, 12q
- Losses: 10q, 16p, 16q
- Focal CNVs: -

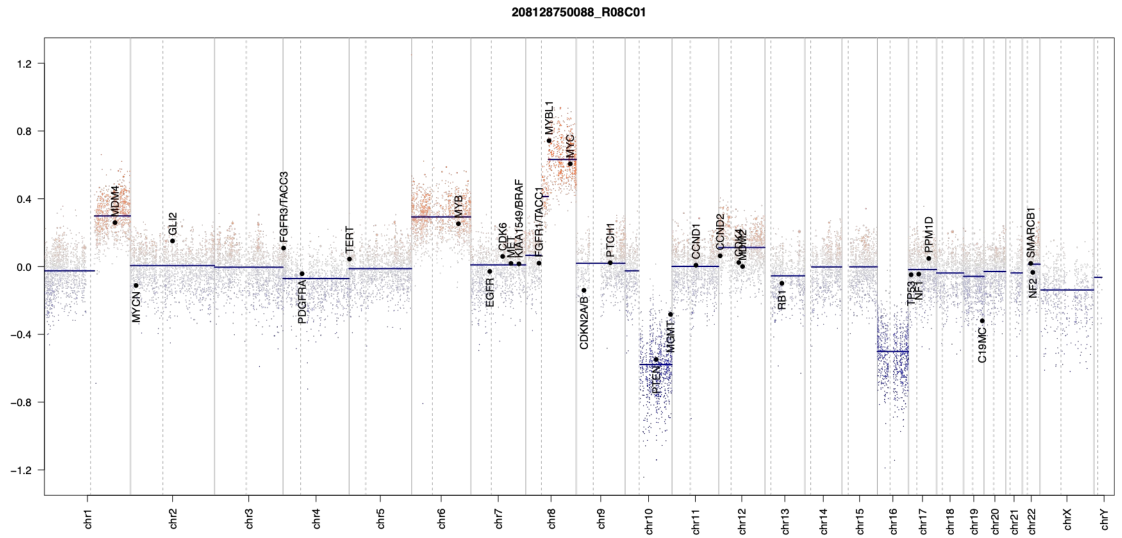

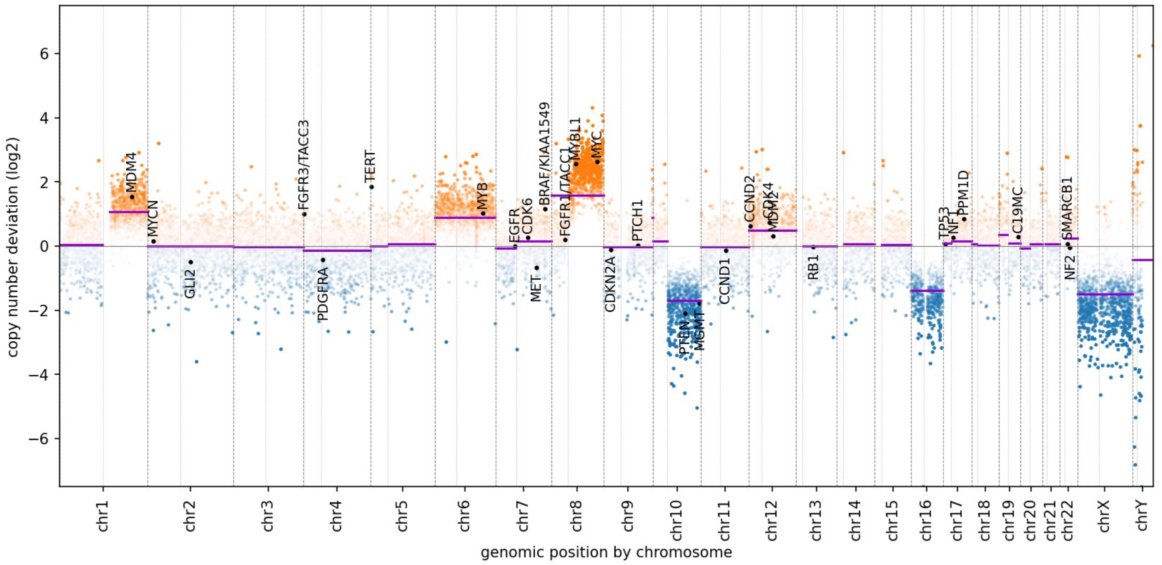

sample #44

- Gains: 7p, 7q
- Losses: 10q, 16q
- Focal CNVs: -

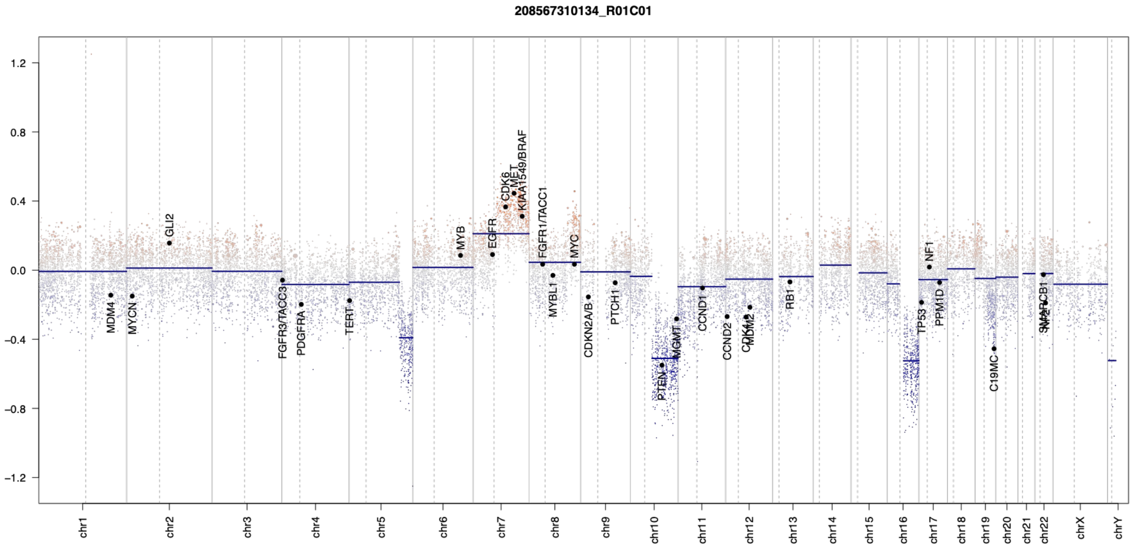

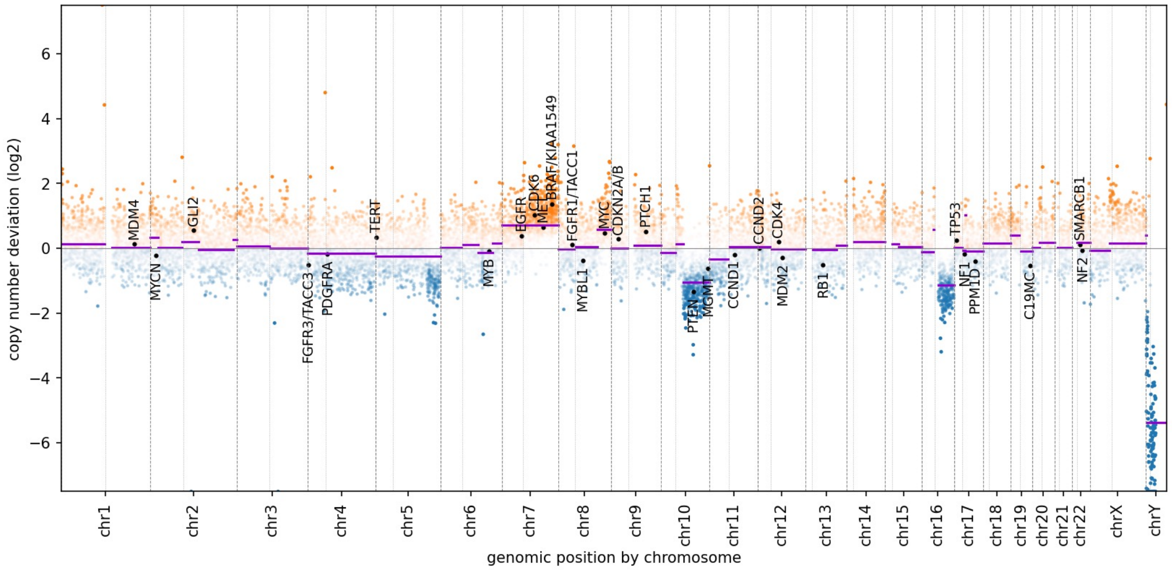

sample #45

- Gains: 3p, 3q, 7p, 7q, 13q, 16p, 16q, 20p, 18p, 18q, 20p, 20q, 21q
- Losses: 11p, 11q, 12p, 12q, 22q
- Focal CNVs: -

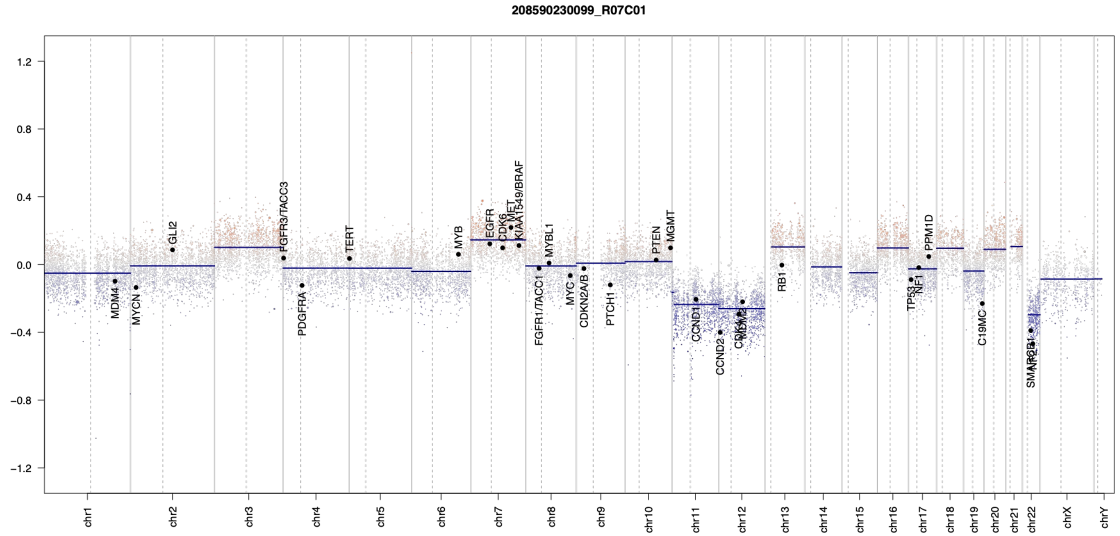

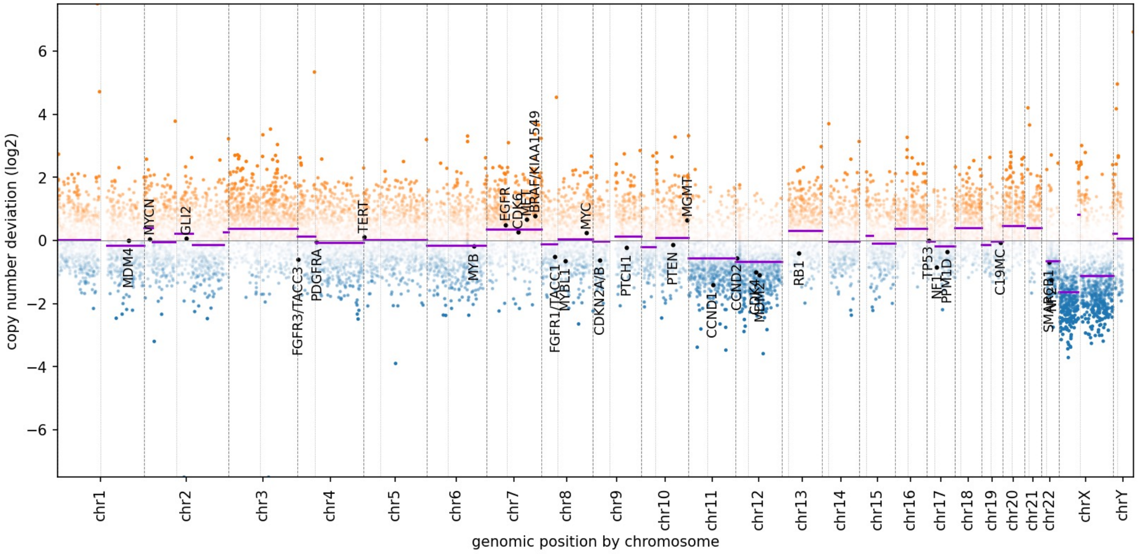

sample #46

- Gains: 2p, 2q, 4p, 4q, 7p, 7q, 10p, 10q, 13q, 17q, 18p, 18q, 20p, 20q, 21q, 22q
- Losses: 17p
- Focal CNVs: -

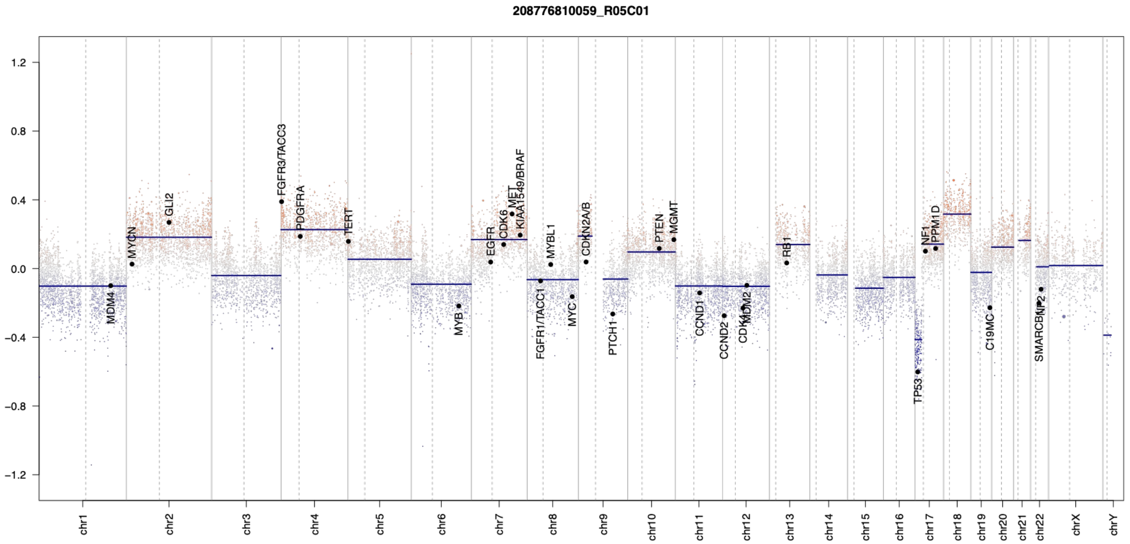

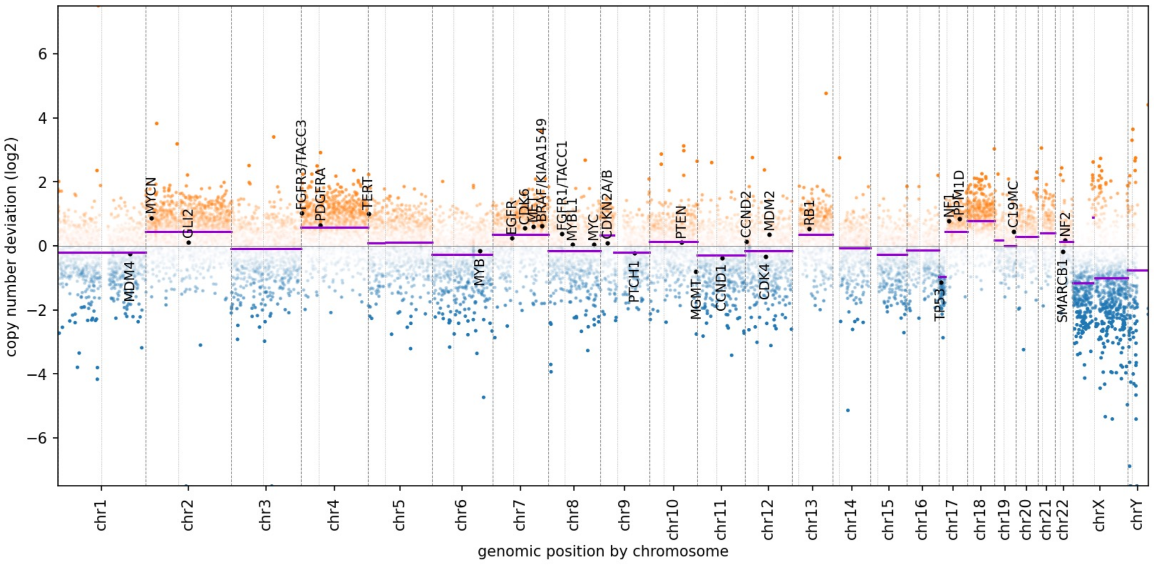

sample #47:

- Gains: 2p, 2q
- Losses: -
- Focal CNVs: -

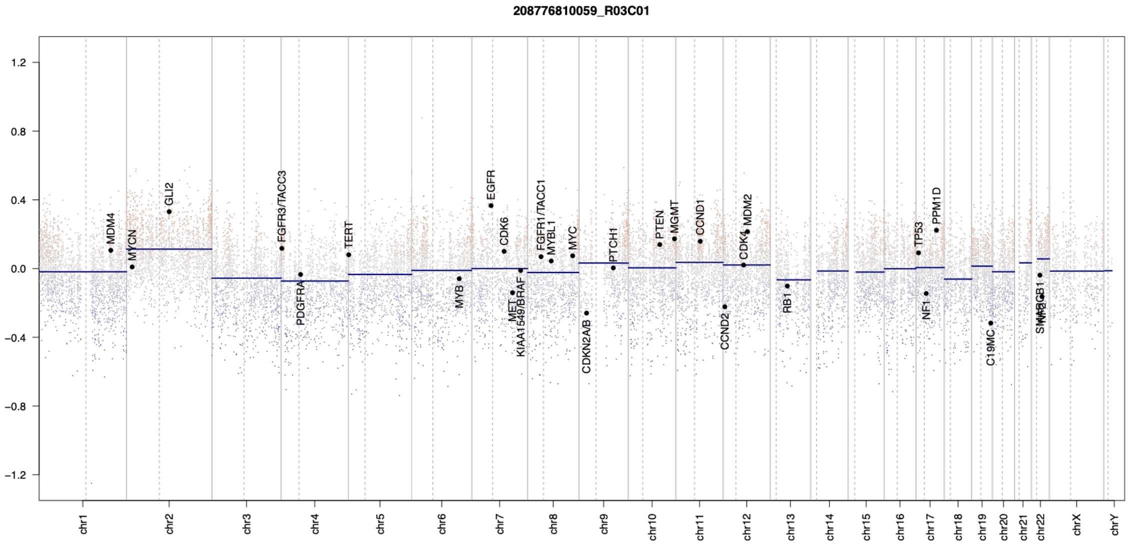

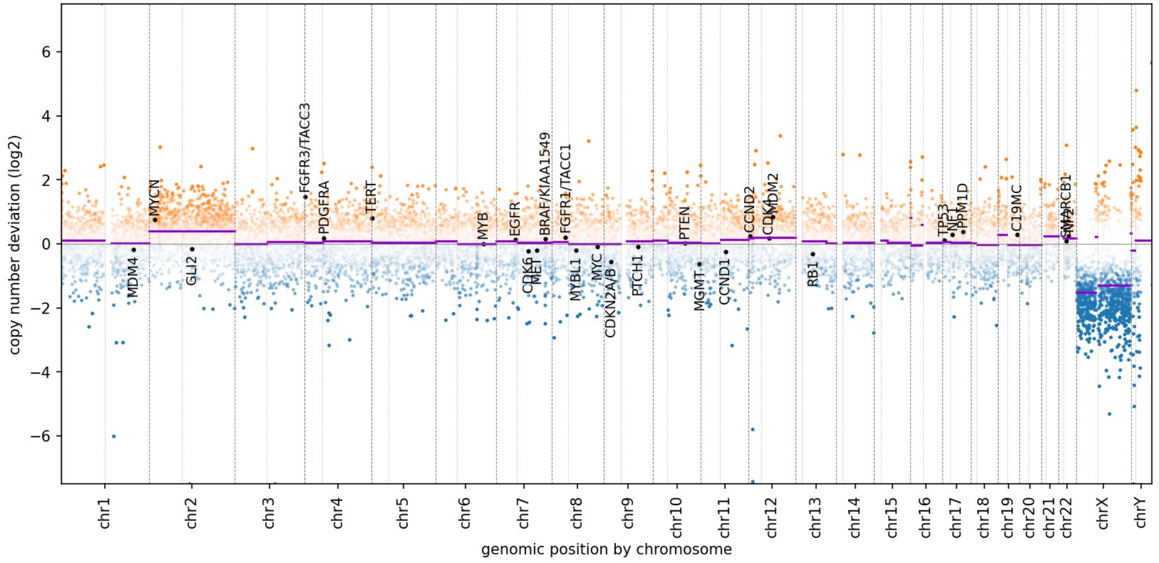

sample #48

- Gains: -
- Losses: 3p, 3q, 6q, 9p, 9q, 22q
- Focal CNVs: -

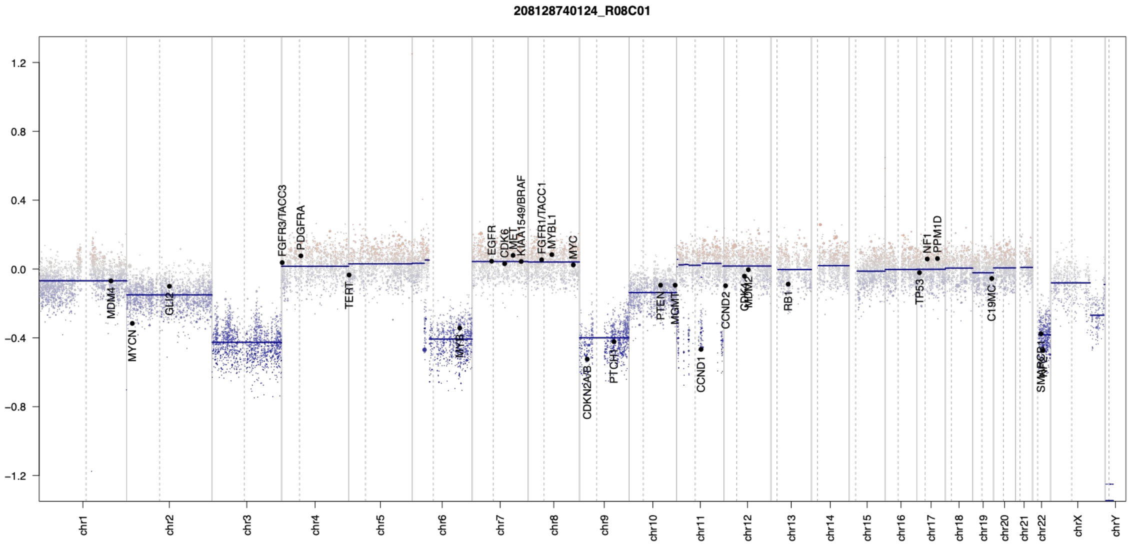

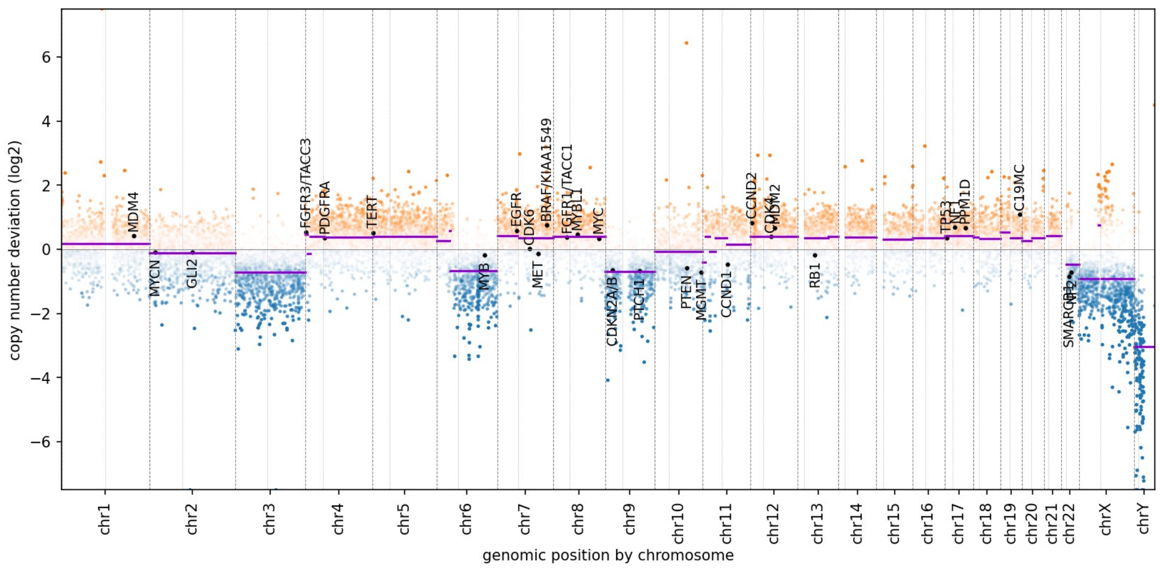

sample #49

- Gains: 1q, 5p, 7p, 7q, 8p, 8q, 9p, 9q, 11p, 11q, 12p, 12q, 13q, 14q, 18p, 18q, 20p, 20q, 21q
- Losses: 22q
- Focal CNVs: -

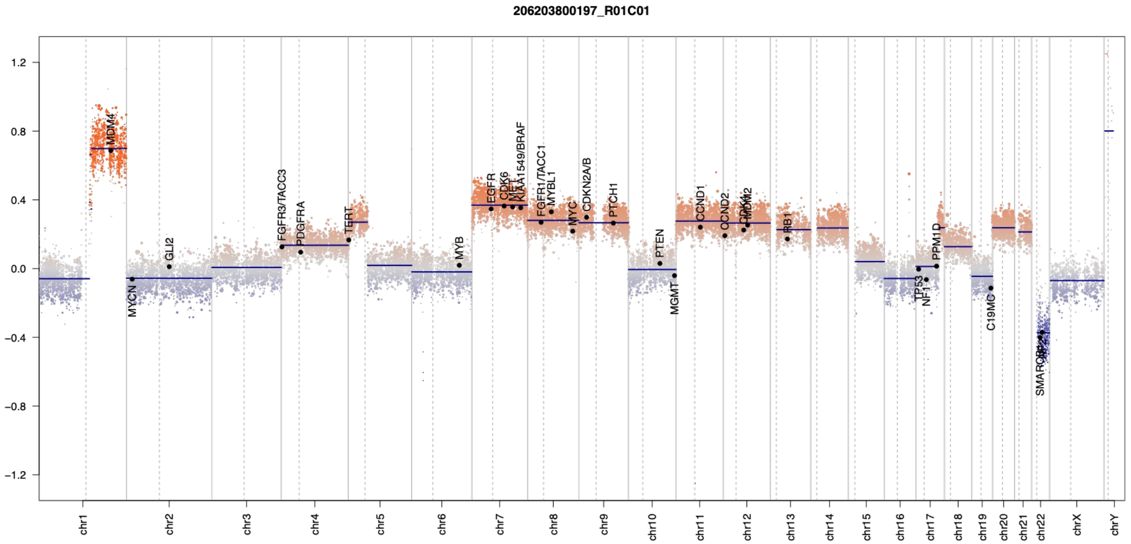

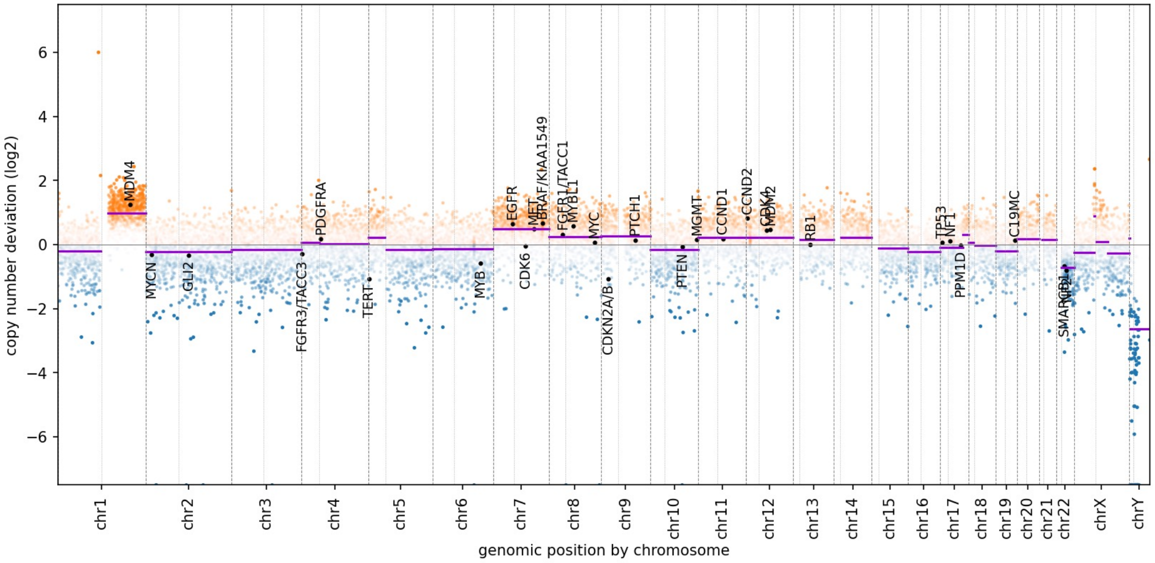

sample #50

- Gains: 1q, 8p, 8q, 10p, 10q, 14q, 18p, 18q, 19p, 19q
- Losses: 16q
- Focal CNVs: -

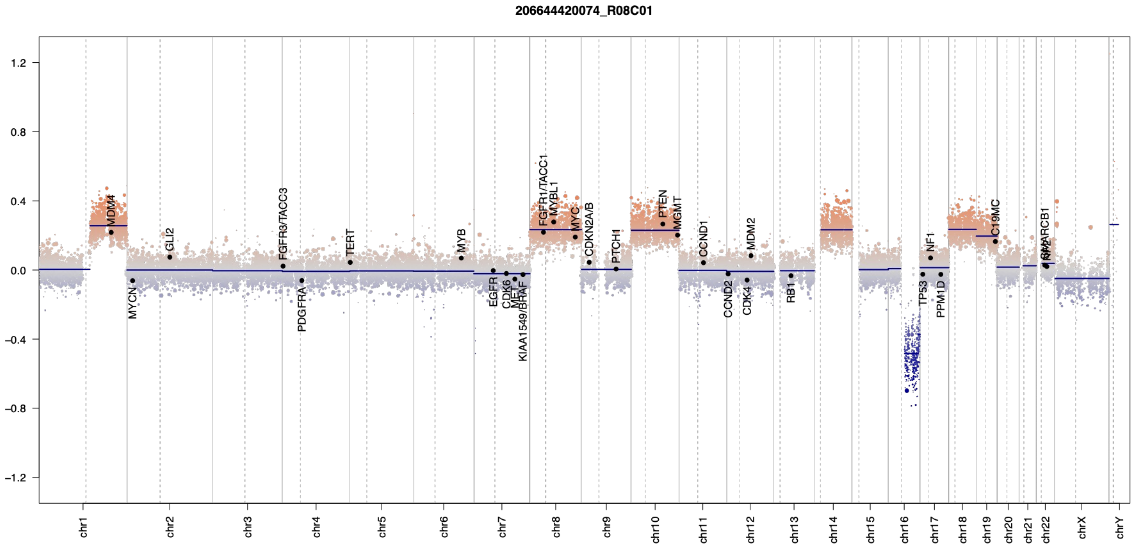

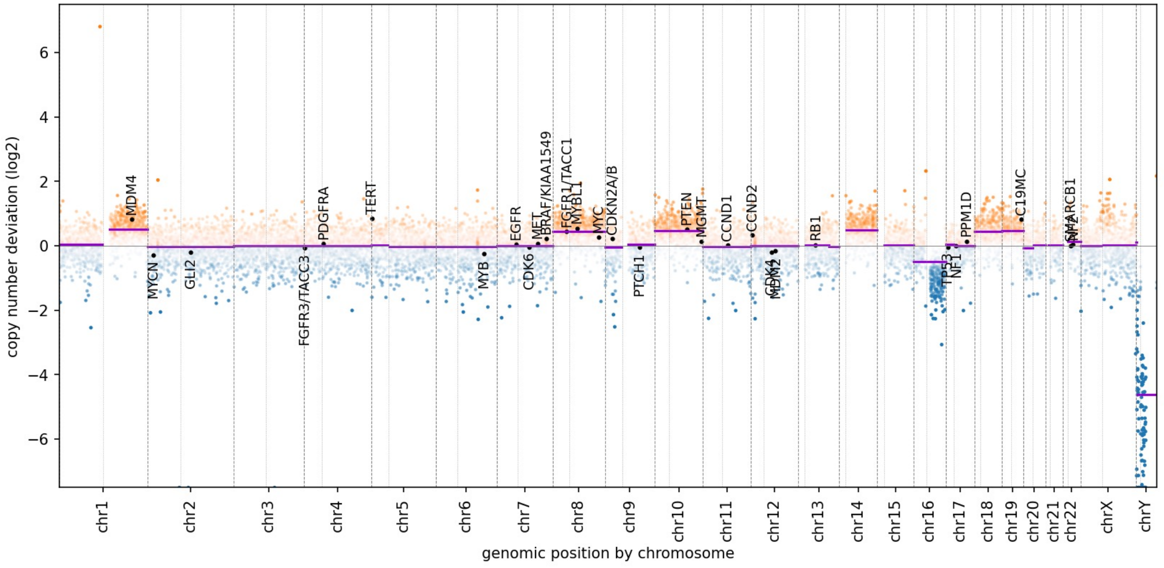

sample #51

- Gains: -
- Losses: 3p, 3q, 5p, 5q, 6p, 6q, 13q, 14q, 17p, 17q, 21q, 22q
- Focal CNVs: -

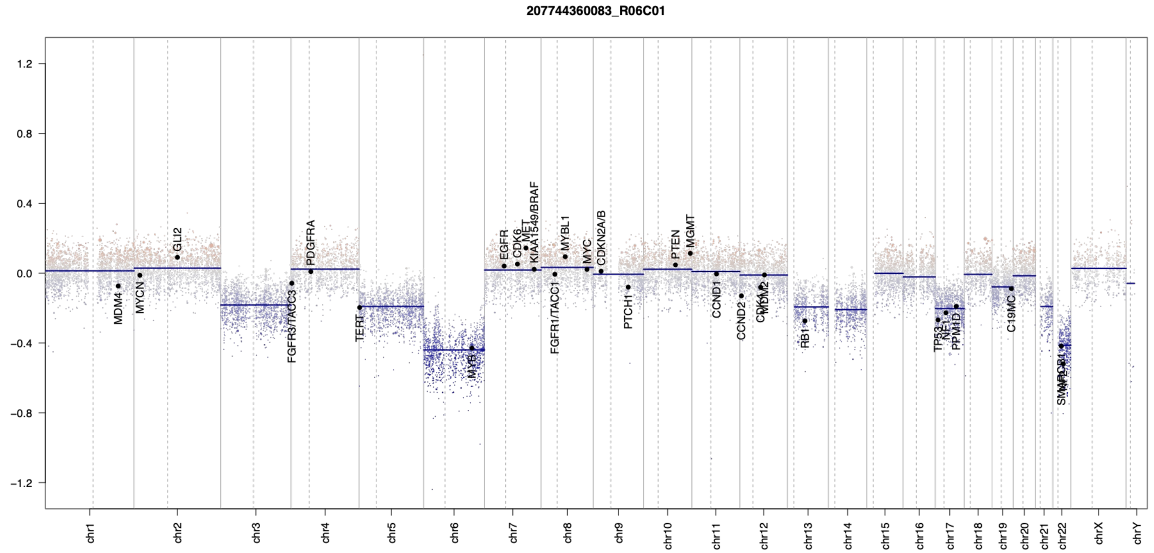

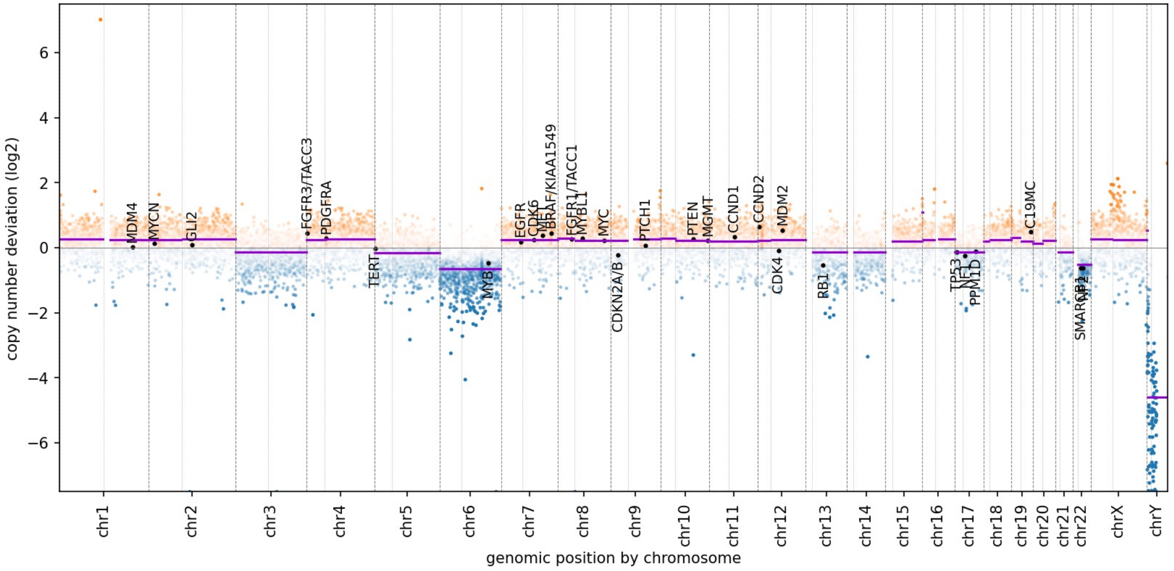

sample #52

- Gains: -
- Losses: -
- Focal CNVs: -

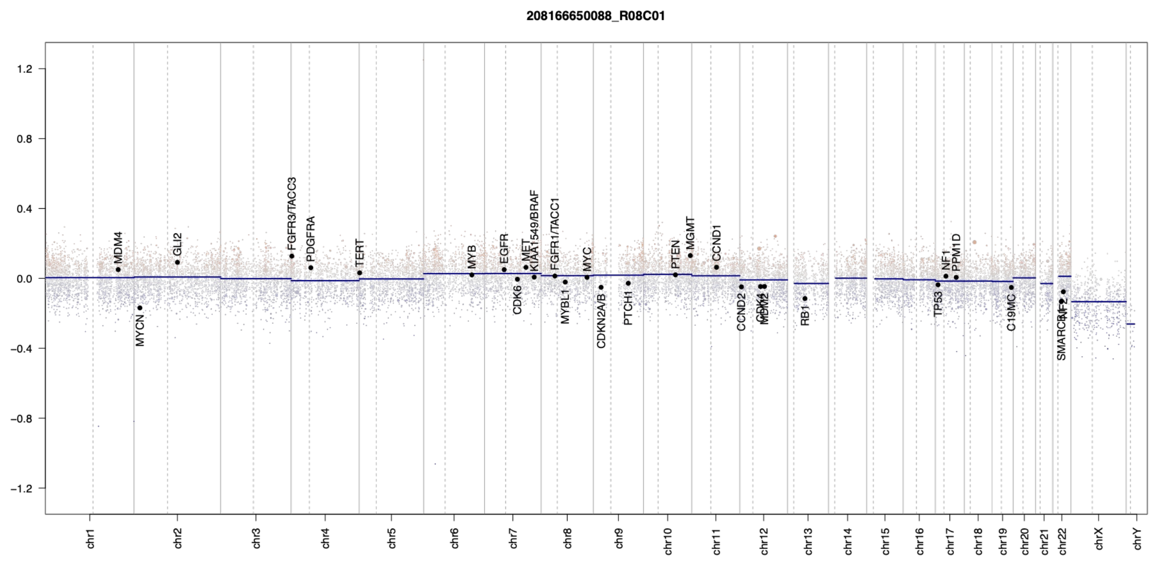

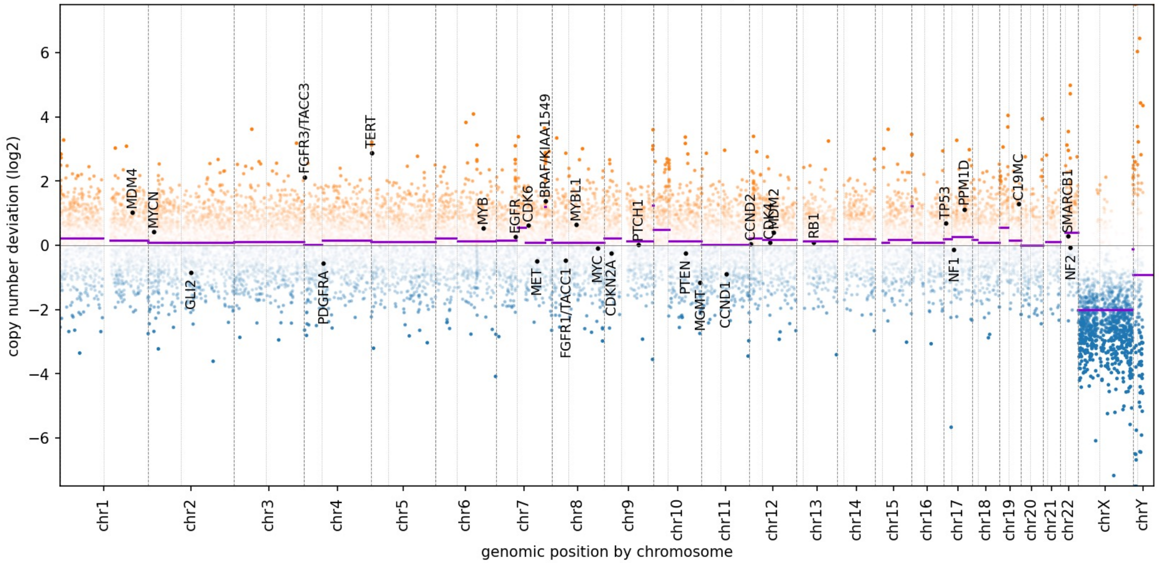

sample #53

- Gains: 5p, 5q, 7p, 7q, 8p, 8q, 9p, 9q, 11p, 11q, 12p, 12q, 14q, 15q, 18p, 18q, 19p, 19q, 20p, 20q
- Losses: 13q
- Focal CNVs: -

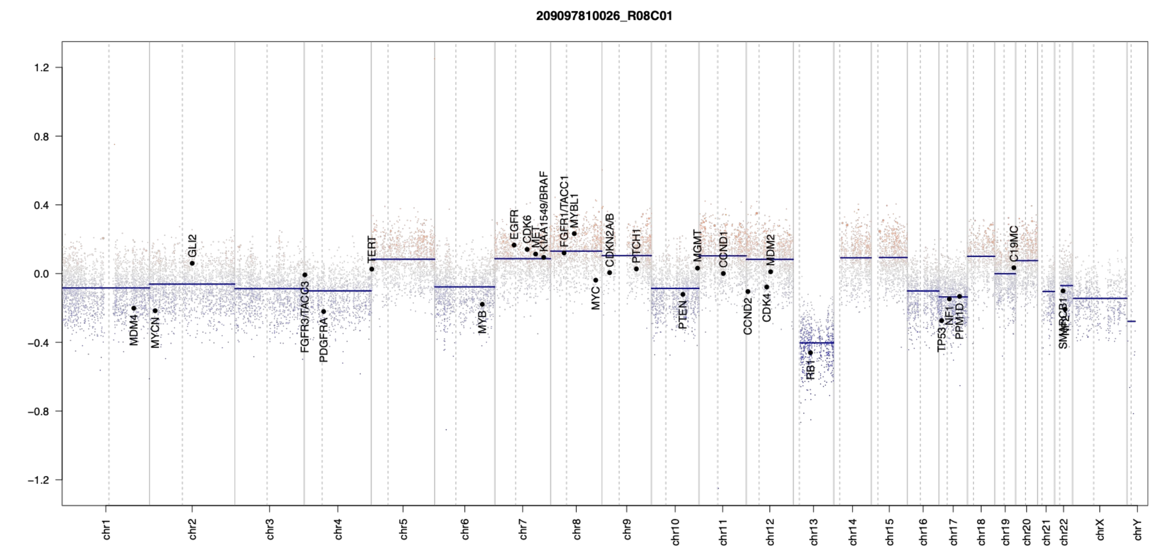

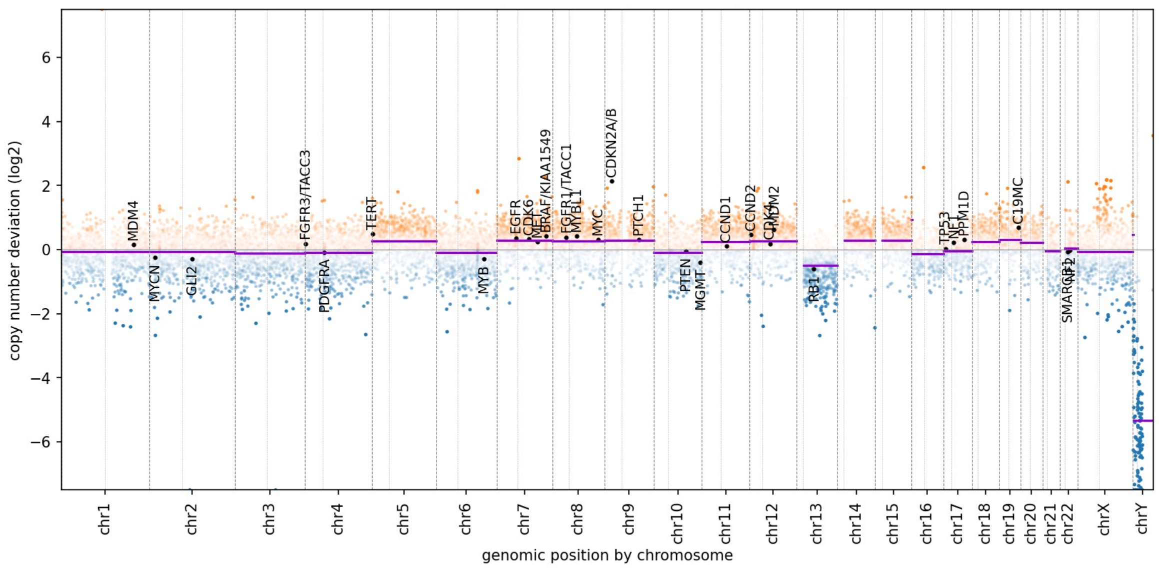

sample #54

- Gains: -
- Losses: 13q, 14q, 21q, 22q
- Focal CNVs: -

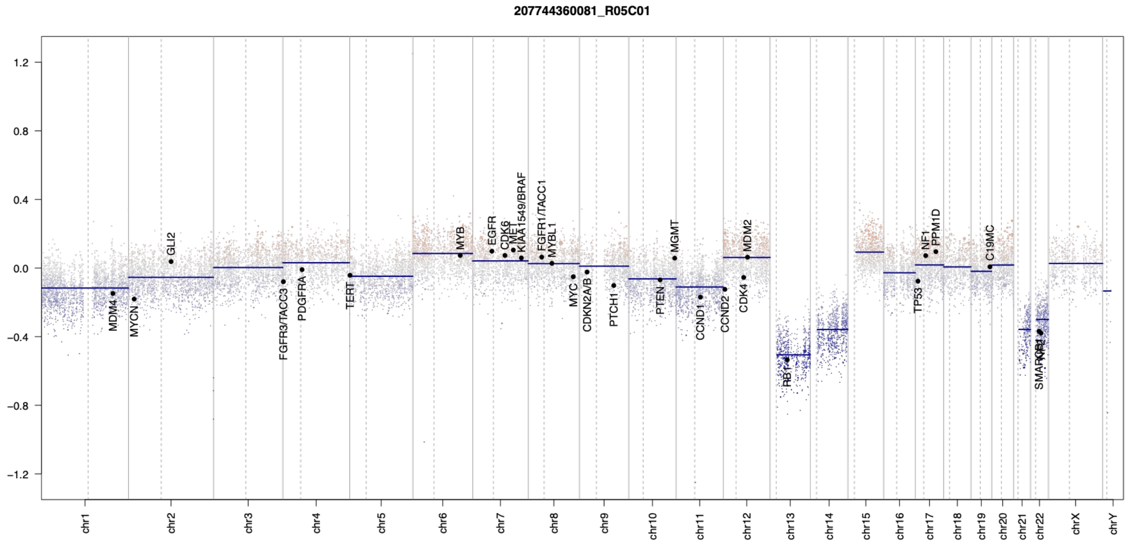

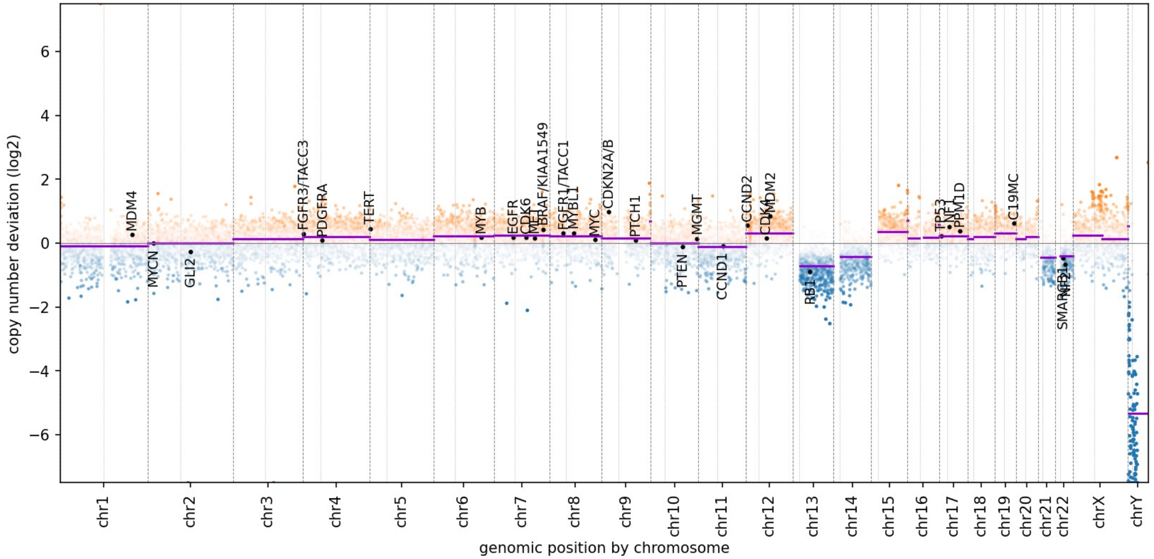

sample #55

- Gains: 18p, 18q
- Losses: 10p, 10q
- Focal CNVs: Amp *MYCN*

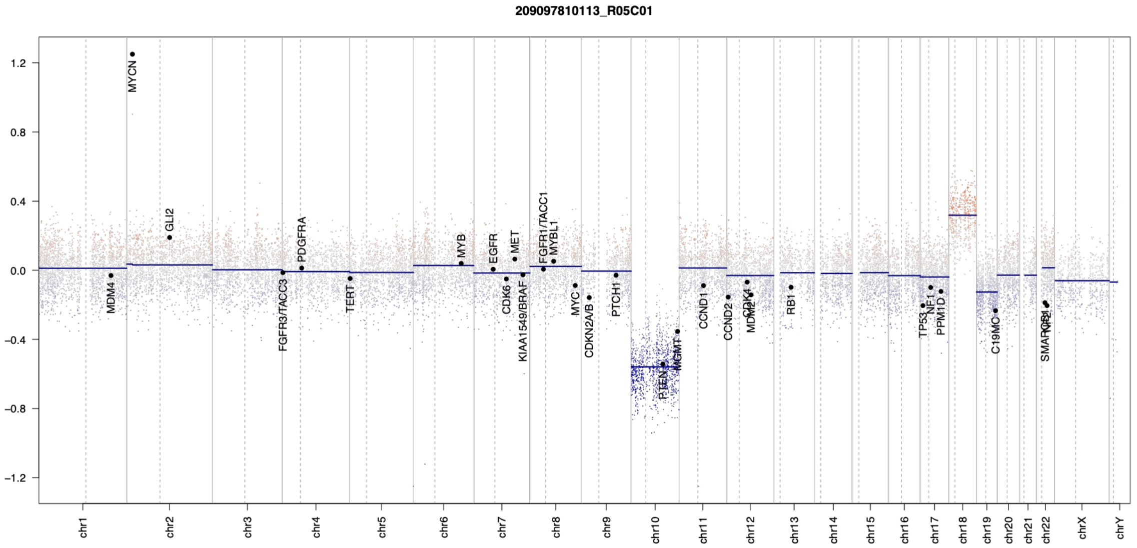

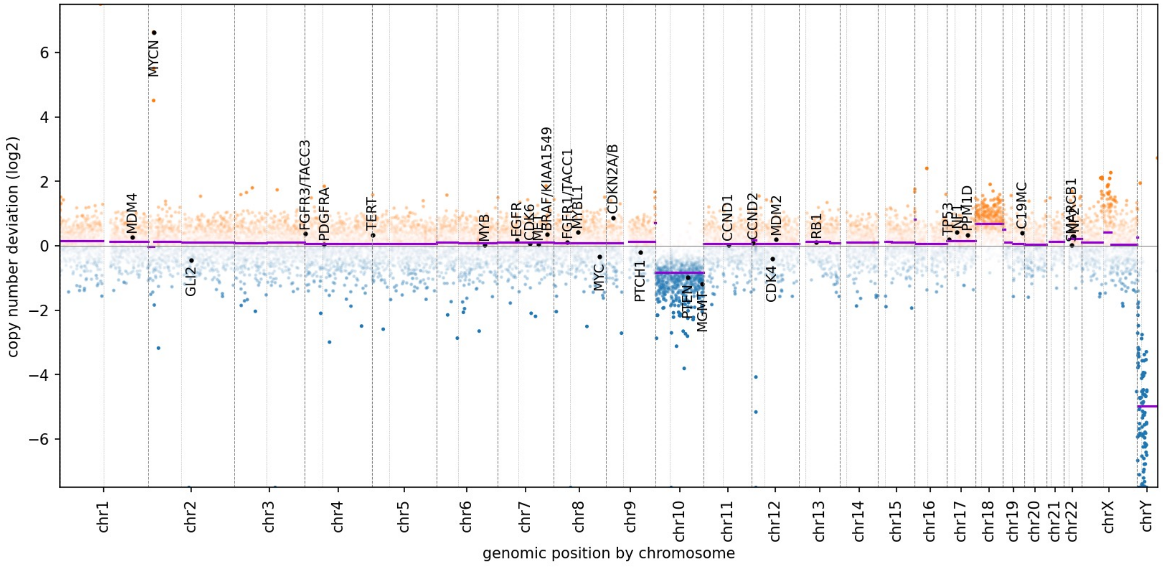

sample #56

- Gains: 3p, 3q, 5p, 5q, 7p, 7q, 9p, 9q, 12p, 12q, 13q, 15q, 17p, 17q, 18p, 18q, 19p, 19q, 20p, 20q
- Losses: -
- Focal CNVs: -

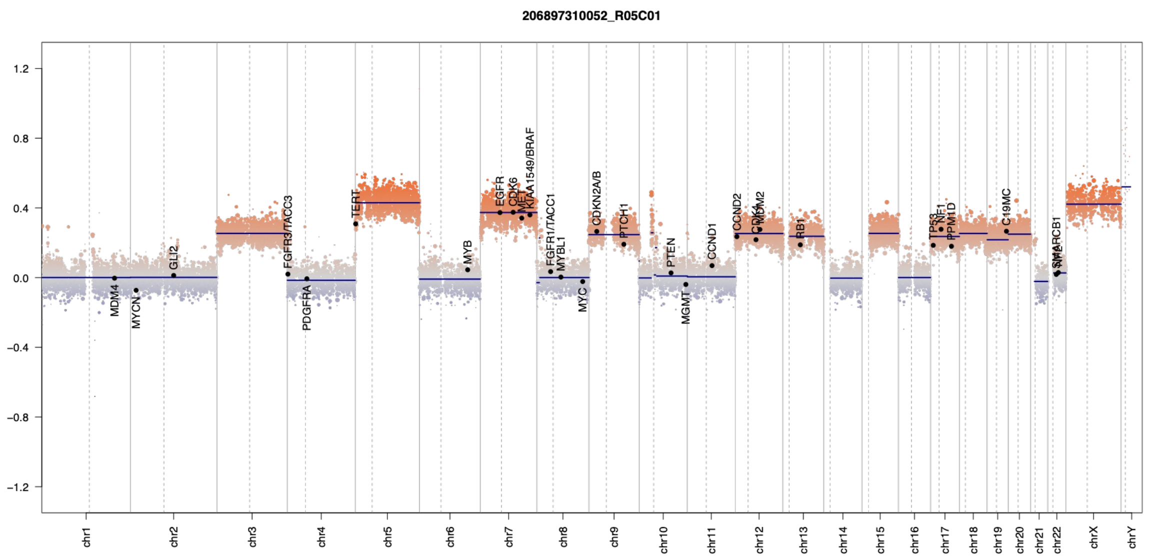

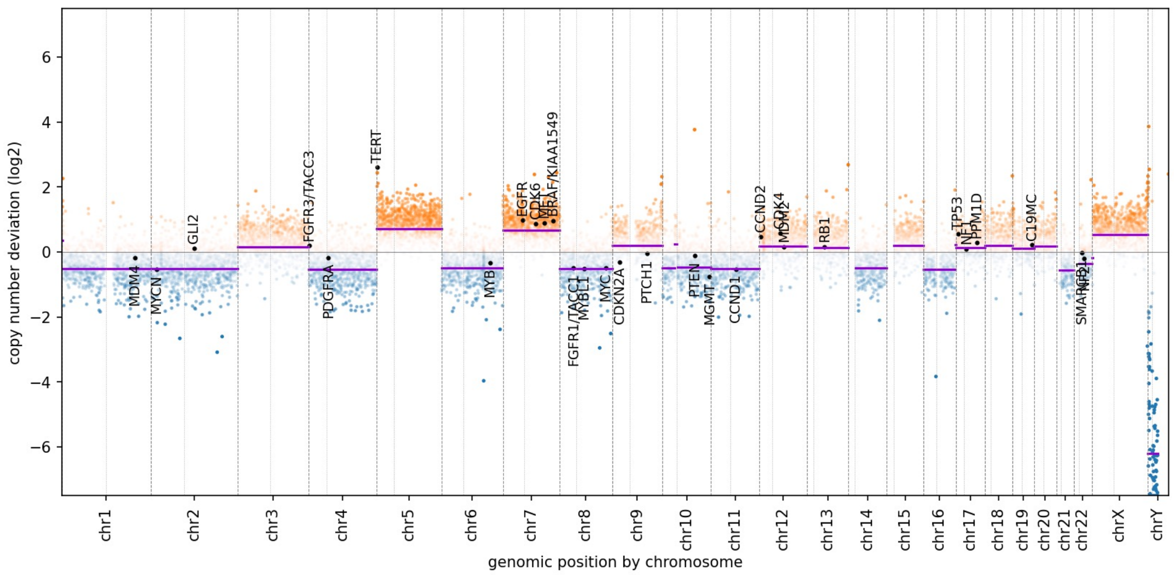

sample #57

- Gains: 7p, 7q, 8p, 8q, 12p, 12q, 14q, 20p, 20q, 21q
- Losses: 10p, 10q, 22q
- Focal CNVs: -

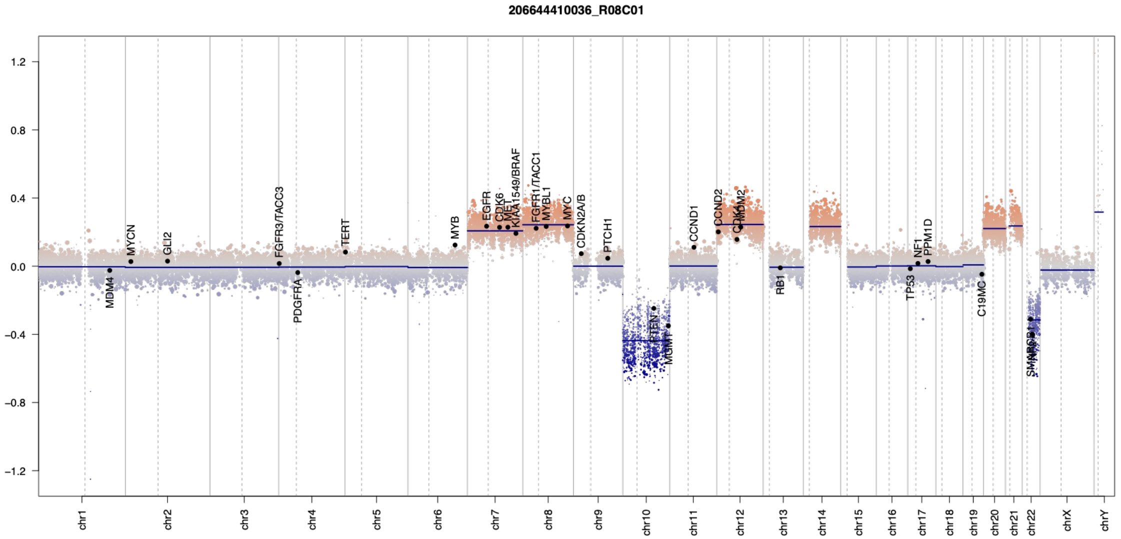

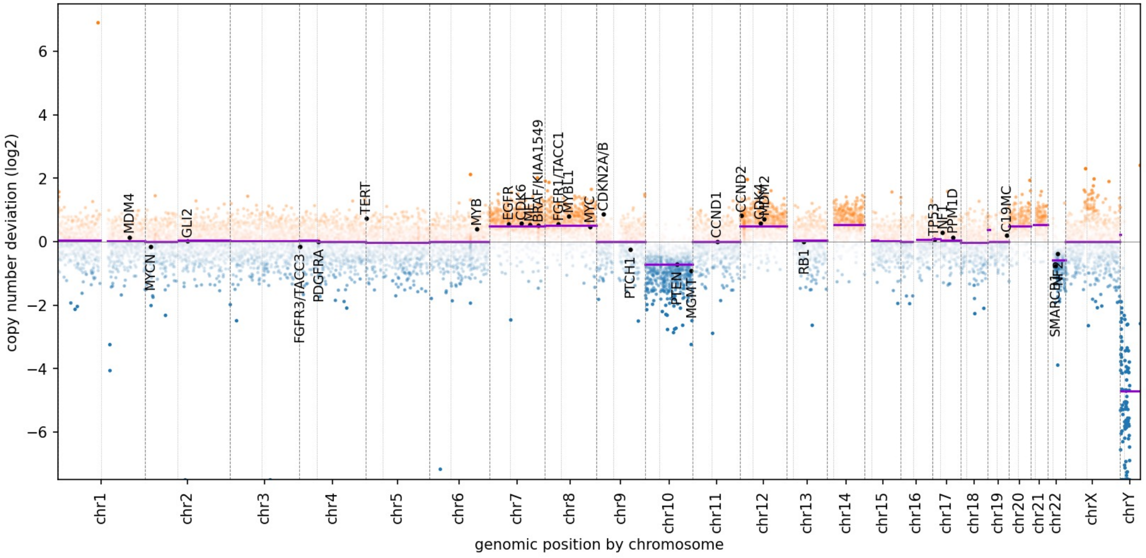

sample #58

- Gains: 5p, 5q, 6p, 6q, 7p, 7q, 9p, 9q, 12p, 12q, 15q, 17p, 17q, 20p, 20q
- Losses: 21q
- Focal CNVs: -

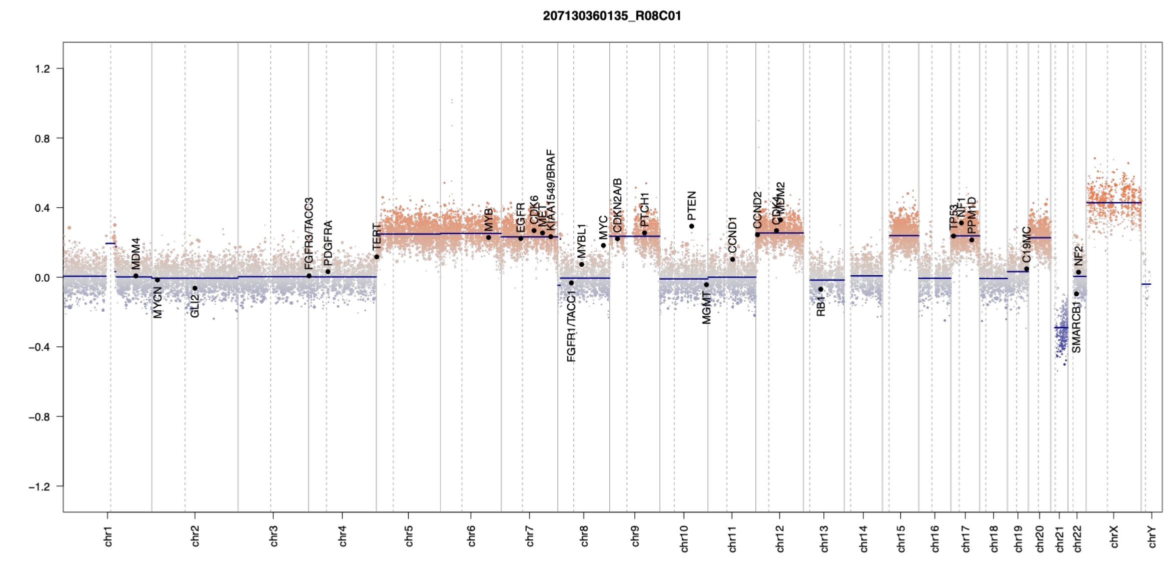

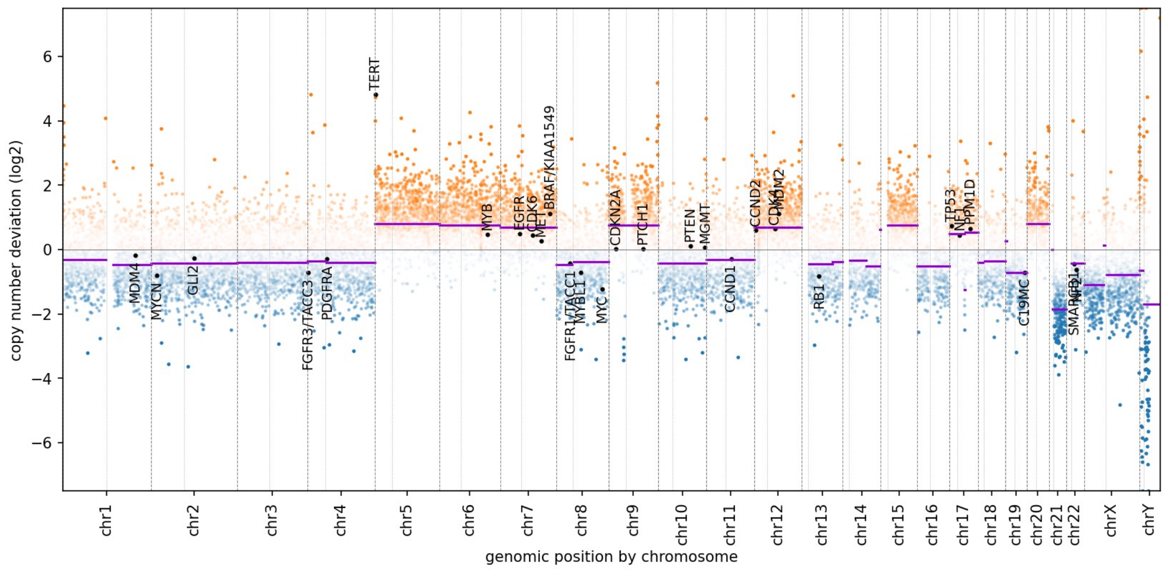

sample #59

- Gains: 1p, 1q, 2p, 2q, 4p, 4q, 8q, 10p, 10q, 13q, 14q, 20p, 20q, 21q
- Losses: -
- Focal CNVs: -

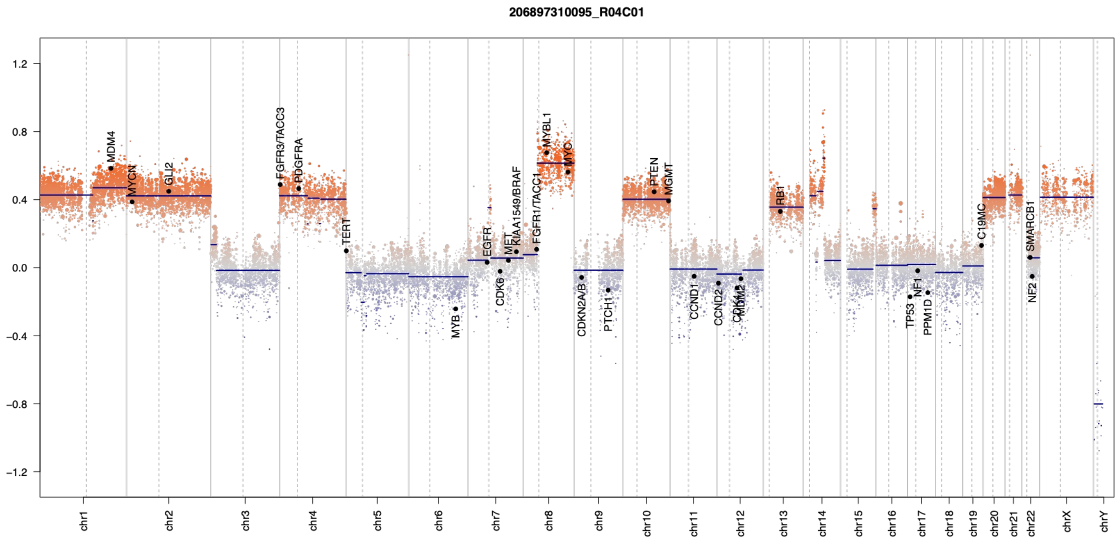

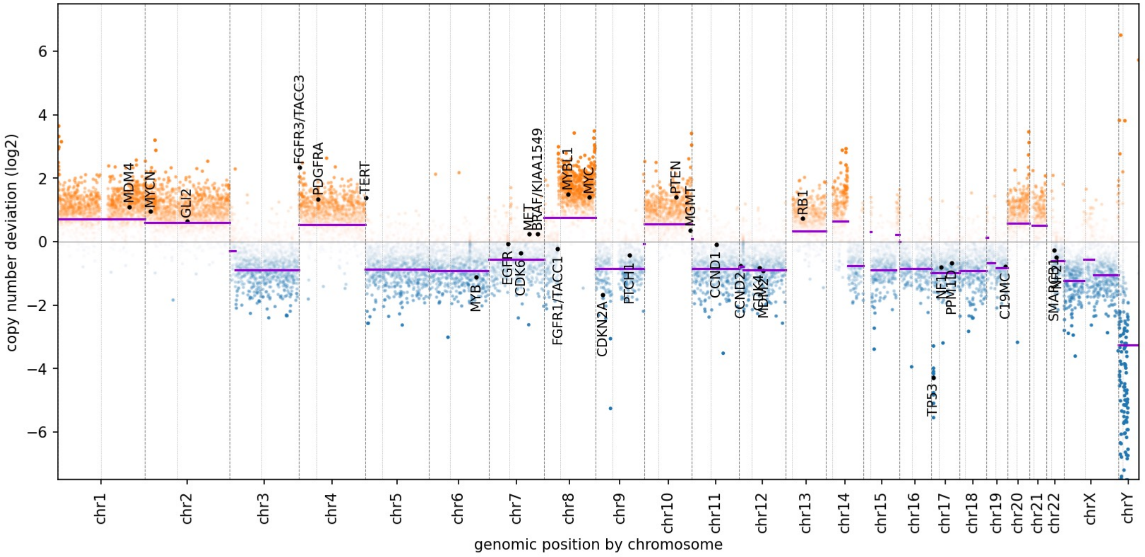

sample #60

- Gains: -
- Losses: 3p, 3q, 5p, 5q, 6p, 6q, 7p, 7q, 8p, 8q, 9p, 9q, 11p, 11q, 13q, 17p, 18p, 18q, 22q
- Focal CNVs: -

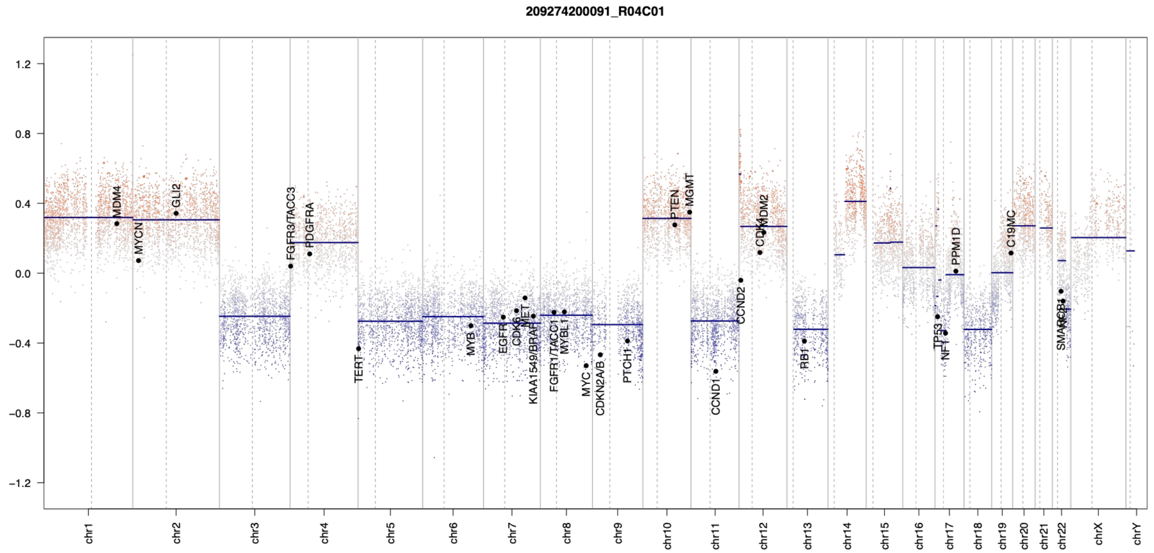

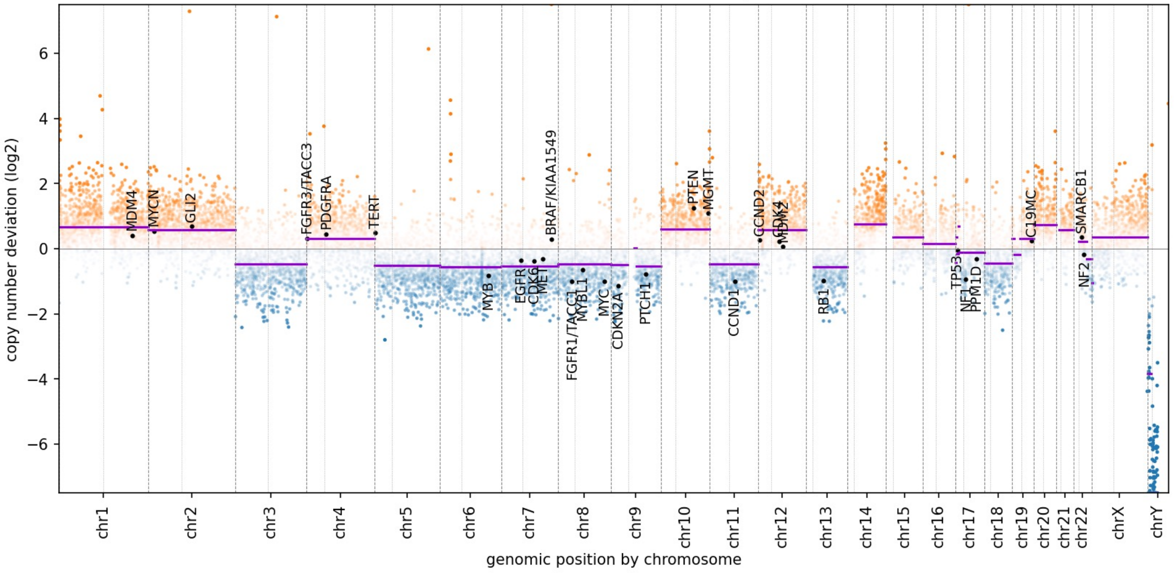

Supplement: Supplementary file 3 — Figure S3: Comparative visualisation of genome‐wide copy‐number profiles derived from DNA methylation arrays and next‐generation sequencing for all 60 analysed cases. [file NAN-52-e70070-s001.pdf]
